# Carbohydrate Physicochemical Properties: The Innate Hydrogen Bond Donating Capacities of α‐Glucoside and α‐Galactoside Alcohol Groups

**DOI:** 10.1002/anie.3574068

**Published:** 2026-06-16

**Authors:** Mrinal Naskar, Zhong Wang, Krunal Patel, Branko De Baets, Anaïs Goupille, Davy Sinnaeve, Eric Renault, Jean‐Yves Le Questel, Bruno Linclau

**Affiliations:** ^1^ Department of Organic and Macromolecular Chemistry Campus Sterre Ghent University Ghent Belgium; ^2^ School of Chemistry and Chemical Engineering University of Southampton Southampton UK; ^3^ CEISAM UMR CNRS 6230 CNRS Nantes Université Nantes France; ^4^ CNRS, Univ. Lille Institut Pasteur de Lille, UMR 9031 – Integrative Structural Biology Lille France

**Keywords:** conformational analysis, cooperative effect, hydrogen bonding, IR spectroscopy, organofluorine

## Abstract

Despite the significance of hydrogen bonding in protein‐carbohydrate interactions, carbohydrate conformation, and crystallinity (solubility), relative hydrogen bond donating capacities (HBDC) of individual alcohol groups of a given sugar are poorly characterised. Here the first systematic determination of the HBDC of individual sugar alcohol groups has been achieved, which were ranked in a HB‐scale (p*K*_AHY_‐scale) that is relevant for medicinal chemistry purposes. HB determination was achieved using an IR‐based protocol with methyl α‐glucoside‐ and α‐galactoside‐based model compounds that exclude any contributions from HB cooperativity effects. A wide variation in HBDC was found, especially for galactose, with a strong stereochemical dependence not only of the alcohol group itself, but also at adjacent and even remote positions. The glucose 4‐OH and, notably, the galactose 6‐OH groups were the strongest donors, whereas the glucose 2‐OH and, notably, the galactose 4‐OH groups were the weakest donors. Interestingly, the galactose 6‐OH is the only group with a stronger HBDC than cyclohexanol. These differences could be qualitatively rationalised by a combined IR, NMR, and computational analysis, pointing to counteracting influences from inductive and the often multiple possible intramolecular hydrogen‐bonding effects. The difference between the factors that determine carbohydrate HB donating capacities and Brønsted acidities is discussed.

## Graphical abstract

Experimental determination of the innate hydrogen bond donating capacities of the glucose and galactose non‐anomeric alcohols revealed significant variations depending on position and relative configuration, which were quantified on the p*K*_AHY_ scale, relevant for MedChem purposes, hence providing another tool toward rational glycomimetic development.

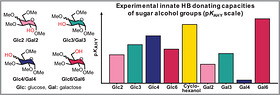

## 1 Introduction

Hydrogen bonding (H‐bonding) is one of the most fundamental non‐covalent interactions in chemistry [1, 2, 3]. It is defined as an attractive interaction between a molecular fragment Y–H, in which Y is more electronegative than H, and an atom Z, in which there is evidence of bond formation [4]. It is a complex, multifaceted interaction involving many energetic components, notably, but not limited to, electrostatic, charge‐transfer, and van der Waals components [3, 5, 6]. The variation in the importance of these leads to a wide variety of hydrogen‐bond (HB) energetics (∼1–40 kJ mol^−1^) [7]. The unique properties of HBs—such as their directional nature, reversibility, cooperativity, moderate strength, and ability to form three‐dimensional networks—are essential for the selective nature of processes ranging from molecular recognition in biological systems to the determination of macromolecular stability, catalytic activity, and material properties. Knowledge of both strong and weak HBs is required for deciphering biological function [7]. In addition, H‐bonding also affects other physicochemical properties, notably solubility and lipophilicity [8, 9, 10, 11].

Carbohydrates are decorated with alcohol groups, which are good hydrogen bond donors and acceptors, resulting in structures that are highly susceptible to forming extensive and cooperative HB networks [12, 13, 14]. Protein‐carbohydrate interactions, which are critical for cellular signalling, immune responses and the formation of extracellular matrices, are to a large extent determined by H‐bonding [15, 16, 17]. Synergistic effects between H‐bonding and hydrophobic/CH–π interactions can lead to high affinities, and it has been demonstrated that the orientation of CH–π stackings is controlled by proximal H‐bonding arrangements [18, 19, 20]. The significant intramolecular hydrogen‐bond (IMHB) propensity of carbohydrates, and, in the case of oligosaccharides, inter‐residue hydrogen bonding, plays a key role in determining mono‐ and oligosaccharide conformation [21, 22, 23, 24]. Additional intermolecular H‐bonding determines to a high extent the crystallinity and solubility of polysaccharides and carbohydrate‐based materials [25, 26]. In addition, differences in reactivities of individual alcohol groups in carbohydrates can often be explained via HB effects [27].

The IMHB patterns of monosaccharides have been extensively studied over the past decades by IR‐analysis in different surroundings, including the gas phase, using the shift of the vibrational frequency of the Y–H stretching modes (Δν_OH_) to assign different IMHB motifs. These O–H absorption band shifts originate from the covalent contribution to HB energetics, consisting of a charge transfer from the acceptor (atom Z) HOMO to the σ*_Y–H_ antibonding orbital [5, 6]. While Δν_OH_ responds to the covalent component of H‐bonding, it can be assumed that the other energetic contributions are very similar given only HBs between alcohol groups are compared.

**FIGURE 1:**
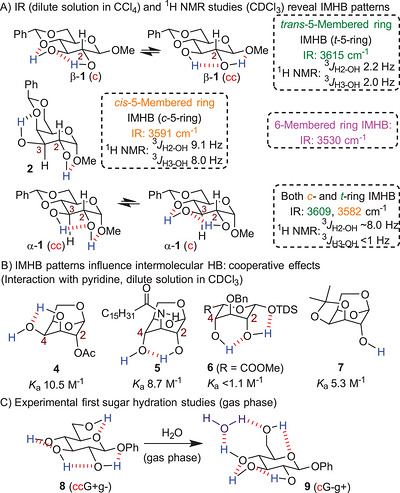
Examples of experimental carbohydrate hydrogen bonding studies (cc = counter‐clockwise, c = clockwise IMHB).

A set of representative examples is shown in Figure 1A. A dilute solution of the β‐glucoside derivative β‐**1** in CCl_4_ displays a single absorption band at 3615 cm^−1^, which results from IMHB between *trans*‐vicinal alcohol groups (*t*‐5‐ring) [28]. The α‐galactosyl derivative **2** displays a single band at a lower wavenumber, 3591 cm^−1^ [29]. This was attributed to the presence of a stronger IMHB between *cis*‐vicinal alcohol groups (*c*‐5‐ring). In contrast, α‐**1** shows two separate bands, the magnitudes of which suggest a counter‐clockwise (cc) IMHB network involving both a *c*‐ and *t*‐5‐ring IMHB [28, 30]. The difference in 5‐ring IMHB strength is attributed to the corresponding alcohol distances and bond angles [30], consistent with the trend in ring strain of *cis‐* vs. *trans*‐hydrindanes [31].

These IMHB assignments are consistent with ^1^H NMR analysis (CDCl_3_) of the vicinal coupling constants between the carbinol and alcohol hydrogen atoms (^3^ *J*_H,OH_). The resulting information about the dihedral angle reveals the position of the alcohol hydrogen, with ^3^ *J*_H,OH_‐values of around 2 Hz or lower indicating a *gauche* dihedral angle, and ^3^ *J*_H,OH_‐values of around 9 Hz or higher indicating an *antiperiplanar* dihedral angle [30]. A freely rotating alcohol group typically displays an intermediate ^3^ *J*_H,OH_ value. This is nicely illustrated by compounds **1** and **2** (Figure 1A).

Hence, general wavenumber ranges for the different types of IMHB have been proposed: absorption bands around 3600–3605 cm^−1^ correspond to a *trans‐*diequatorial‐substituted donor‐acceptor pair, bands around 3580–3585 cm^−1^ to a *cis*‐substituted pair [32, 33, 34], and for a 6‐membered IMHB a (broad) band around 3530 cm^−1^ is observed [30, 34].

Cooperative effects describe the change of HB properties of a given functional group when that group is also hydrogen bonded to another functional group. An alcohol group acting as an HB acceptor will have an enhanced HB donating capacity (HBDC), and an alcohol group acting as a donor will be a stronger acceptor [35]. Seminal experimental illustrations of the influence of HB networks were provided by the Vicent group [36]. Compounds **4** and **5** (Figure 1B) were shown to display enhanced HB donating capacity compared to the mono‐alcohol **7**, as measured by the association constant with pyridine in CDCl_3_, which was explained by a positive cooperative effect. In contrast, the diol **6** displayed a negligible association constant, which was related to its IMHB involvement. However, the geometric changes required for cooperativity are limited by the rigidity of most of the alcohol groups on the tetrahydropyran ring system [37]. In addition, the first IMHB was found to have a larger effect than subsequent interactions [37, 38].

Much research has been devoted to the understanding of the HB interactions of sugars with water (explicit carbohydrate hydration). Seminal experimental data came from the work of Simons and Davis, for example, showing that single hydration, such as that of the glucose derivative **8** (Figure 1C), can lead to conformational changes, as illustrated by the structure of **9** [39, 40].

Alongside experimental spectroscopic techniques used to characterise hydrogen‐bonding interactions in carbohydrates across different physical states, from the gas phase to solution, theoretical approaches have been extensively employed to gain molecular‐ and electronic‐level insight into these systems [41]. Despite these numerous studies, a quantitative and site‐specific description of the hydrogen‐bond donor ability of individual hydroxyl groups in carbohydrates in solution remains elusive. Indeed, despite the extensive research into carbohydrate H‐bonding, there is little quantitative experimental data available regarding relative HB properties of hydroxyl groups on carbohydrates. In contrast, nucleophilicity tables of carbohydrate alcohols (for glycosylation) [42], as well as Brønsted acidity tables of carbohydrate alcohols [43] and their amino sugar analogues [44], have been reported. This is a clear knowledge gap toward the understanding of carbohydrate physicochemical properties.

While the use of carbohydrates in drug discovery has traditionally been limited by their high hydrophilicity [45], the development of glycomimetics has led to successful therapeutics [46, 47, 48, 49]. It can be posited that the renewed recognition [50, 51] that drugs should exhibit maximal hydrophilicity within the limits imposed by membrane permeability [52] will further stimulate glycomimetic drug discovery. Given the significance of HB donors in drug discovery [53], mapping the intrinsic HB donating capacity of carbohydrate hydroxyl groups on a quantitative HB donor scale that is relevant to medicinal chemistry and understanding the factors that influence their HB donating capacity will greatly support rational glycomimetic drug design and, more generally, the design of sugar‐based probes for glycobiology research.

Here we report systematic experimental measurements of the intrinsic hydrogen bond donating capacities of individual hydroxyl groups in α‐glucoside‐ and α‐galactoside derivatives. Using the p*K*_AHY_ hydrogen bond donating capacity scale, we present the first quantitative ranking of sugar hydroxyl groups according to HBDC. Our findings demonstrate a significant dependence on hydroxyl position and stereochemistry, resulting in a broad range of HBDC values. This positional and stereochemical dependence could be qualitatively rationalised by detailed IR and NMR spectroscopic studies, supported by DFT calculations. Furthermore, we show that the factors governing HB donating capacities in sugar alcohols differ markedly from those influencing their Brønsted acidities.

## 2 Results and Discussion

### 2.1 The HB Determination Methodology

The selection of an HBDC measurement method is based on the understanding that H‐bonding can be described as a Lewis acid‐base interaction: the HB donor acts as a Lewis acid by accepting electron density, while the HB acceptor functions as a Lewis base by donating electron density [55]. Following the IUPAC definition for Lewis acidity/basicity, HBDC is then defined as the thermodynamic tendency of a substrate to act as a HB donor [56, 57], and the term HB acidity is also applicable. Hence, a scale based on a thermodynamic equilibrium is applied.

**FIGURE 2:**
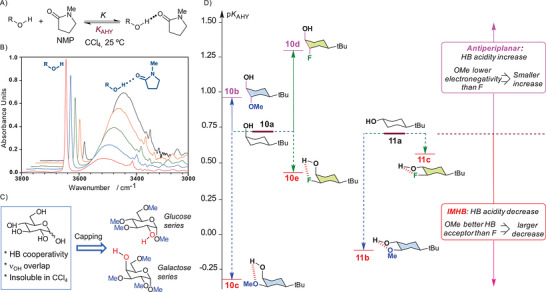
(A, B) The p*K*_AHY_ scale and IR‐based measurement. Incremental addition of N‐methylpyrrolidinone (∼5.0−10.0 × 10^−2^ M) leads to an increase in absorbance of the complexed ν_OH_ band (red → black), with a concomitant decrease in the free ν_OH_ band (red → black). (C) Strategy for sugar alcohol measurement. (D) Model systems featuring vicinal methoxylation and comparison with reported [54] vicinal fluorination.

The p*K*_AHY_ scale is a measure of HB donating capacity, defined (Figure 2A) as the inverse equilibrium constant for the complexation between the substrate and a standard acceptor, *N*‐methyl pyrrolidinone (NMP), in an apolar solvent, carbon tetrachloride (CCl_4_) [58]. The scale is designed so that increasing HB acidity corresponds to higher p*K*_AHY_‐values. Measurements are based on the decrease in absorbance of the ν_OH_ stretching band upon NMP addition (Figure 1B), which allows concentration determination. We have shown that the p*K*_AHY_ value can be predicted by the Kenny parameter (*V*_α_(*r*) value) [54, 58], which is defined as the molecular electrostatic potential calculated at a distance *r* from the donor hydrogen atom along the HB axis [59]. We have also shown that conformational *V*_α_(*r*) values can be used to evaluate the HB donating capacity of individual conformers. In addition, the *V*_α_(*r*) values correlate with intramolecular hydrogen bonding: the stronger the IMHB, the lower the *V*_α_(*r*) value [60].

At this stage, the aim was to specifically target the intrinsic HB acidity of individual sugar alcohol groups, avoiding perturbations from cooperativity effects, thus enabling a precise evaluation of the influence of stereochemistry and position. Measurements of individual alcohol HB acidities were achieved by capping the remaining hydroxyl groups as methyl ethers (Figure 2C). This capping also provided significant practical benefits, such as eliminating otherwise unavoidable ν_OH_ stretching band overlaps and ensuring sufficient solubility in CCl_4_, which is essential to prevent interference from intermolecular associations. In all cases, the absence of intermolecular association was verified for the concentrations used (0.01–0.06 M).

Initially, 4‐*t*‐butyl cyclohexanol models were employed to evaluate how the HBDC of isolated alcohols is affected by vicinal methoxylation in function of relative configuration (Figure 2D). As expected, when antiperiplanar (**10b**), a higher HBDC was measured due to the OMe electronegativity. In contrast, with vicinal gauche substitution (**10c**, **11b**), a much lower HBDC capacity was measured, with p*K*_AHY_‐values even lower than zero. This is attributed to intramolecular H‐bonding with OMe competing with intermolecular H‐bonding with NMP. The comparison with the corresponding fluorohydrin derivatives [54] is instructive: with antiperiplanar disposition (**10d**), the larger fluorine electronegativity leads to a higher HBDC increase, but for *gauche*‐fluorohydrins (**10e**, **11c**), a much lower decrease was observed, due to the worse H‐bond acceptor capacity of fluorine compared to oxygen [61, 62]. The larger decrease for **10e** and **10c** compared to **11c** and **11b** is consistent with the stronger IMHB for *cis*‐vicinal substitution compared to *trans‐*1,2‐diequatorial substitution (see above, Figure 1A), as evident from the respective absorption band redshifts (Supporting Information, Section 1.3).

### 2.2 HB Acidity of the α‐Glucoside OH Groups

**FIGURE 3:**
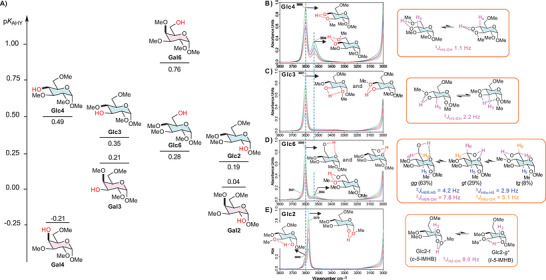
(A) The HB donating capacity of glucose and galactose alcohol groups; (B–E) IR spectra of the glucose derivatives (O–H stretching region, increasing dilution 0.06–0.01 M in CCl_4_) with the corresponding relevant ^1^H NMR data.

The p*K*_AHY_‐values of the glucose alcohol groups are shown in Figure 3A. These are clearly much higher than those of **10c** and **11b**, albeit not as high as the values for cyclohexanols **10a** and **11a**. The following HBDC trend is observed: 4‐OH > 3‐OH > 6‐OH > 2‐OH.

The high pK_AHY_‐value for the 4‐OH group is attributed to inductive effects. It has two vicinal electronegative substituents, one of which (the ring oxygen) is antiperiplanar. The resulting HBDC‐enhancing effect is nevertheless to a certain degree compensated by competition from a *trans‐*5‐membered ring IMHB (*t‐*5‐IMHB) with 3‐OMe and a 6‐membered ring IMHB (6‐IMHB) with 6‐OMe, as indicated by the two OH‐absorption bands in the IR spectrum of **Glc4** (Figure 3B). The 6‐IMHB is stronger, hence its lower wavenumber, but the ring geometry requires two parallel C–O bonds, which in CCl_4_ results in a destabilising dipole‐repulsion contribution [63], which would be responsible for its lower population.

The 3‐OH (**Glc3**), the next best donor, is flanked by two electronegative vicinal‐*gauche* methoxy groups, which are also possible HB acceptors to form weak *t‐5‐*IMHBs (Figure 3C). The IMHB patterns of **Glc4** and **Glc3** require a H, OH *gauche* dihedral angle, which is consistent with the observed ^3^ *J*_H, OH_ values of 1.1 and 2.2 Hz, respectively (measured in CDCl_3_).

The 6‐OH group (**Glc6**) is adjacent only to one electronegative substituent and hence experiences less influence from the cumulative electron‐withdrawing effect of the other sugar oxygen atoms. The IR spectrum (Figure 3D) indicates minimal competition from a 6‐ring IMHB. This observation aligns with the low population of the *tg*‐conformer, as derived from analysis of a combination of the ^3^ *J*_H5‐H6_ coupling constants (in CDCl_3_) using the equations established by Crich et al. [64], in combination with the ^3^ *J*_H6‐OH_ coupling constants (see Supporting Information, Section 2). This analysis also indicated that there is a 2:1 ratio of *gg‐* and *gt*‐conformers, which is consistent with the larger ^3^ *J*_H, OH_ value of the *pro‐R* H6 compared to the *pro‐S* proton. These trends are also in line with the DFT computed relative energies (the *gt* and *tg* conformers are calculated to be +7.6 and +7.8 kJ/mol, respectively, less stable than the *gg* conformer, see Supporting Information, Section 3). In this scenario, both *cis*‐ and *trans*‐5‐IMHBs are present, despite a single sharp absorption band is seen, which we assume results from the ability of the conformationally flexible primary alcohol to adopt a more optimal geometry for IMHB, resulting in a stronger IMHB.

Finally, the glucose 2‐OH group is the least strong HB‐donor, which is surprising, as the 2‐OH group is also antiperiplanar with the ring oxygen and moreover adjacent to the more electron‐withdrawing C1‐acetal group. It seems that there is a larger reduction in HB acidity due to a strong *cis*‐5‐ring IMHB (*c*‐5‐ring IMHB) with the axial anomeric OMe group, as revealed by the IR spectrum of **Glc2** (Figure 3E), which shows an absorption band at a lower wavenumber compared to all other 5‐IMHB rings, and which strongly outcompetes the *t‐*5‐IMHB to the 3‐OMe group. This situation is also consistent with the observed 8.0 Hz vicinal coupling constant between H2 and the alcohol hydrogen, which indicates a predominantly antiperiplanar disposition (**Glc2‐*t*,** compare with **2** in Figure 1). This is consistent with the lower DFT‐calculated energies of **Glc2‐*t*** compared to **Glc2‐*g^+^*** (> 5 kJ/mol), and the lower *V*_α_(*r*)‐value of the former (see Supporting Information) [60]. This situation was not expected given the worse HB accepting capacity of an anomeric acetal oxygen atom.

### 2.3 HB Acidity of the α‐Galactoside OH Groups

**FIGURE 4:**
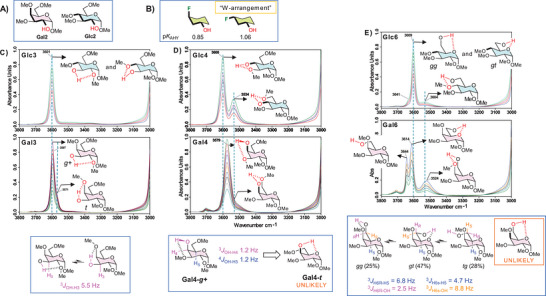
(A–E) Comparison of the Glc and Gal IR spectra (O–H stretching region, increasing dilution 0.06–0.01 M in CCl_4_).

The HB acidity trends and variation among galactose alcohols is very different (Figure 4), notably at the 4‐ and 6‐positions: while OH‐4 is the strongest HB donor in the glucose series, it is the weakest donor in the galactose series, and the galactose 6‐OH is the strongest HB donor overall, despite being a primary alcohol.

The reduced HB acidity of **Gal2** compared to that in **Glc2**, despite its strong OH2 *c*‐5‐ring IMHB is slightly less abundant (^3^ *J*_H2,OH_ = 7.1 vs. 8.0 Hz for **Glc‐2**), is explained by the reduced electronegativity of the axial 4‐OMe group, compared to the equatorial group in glucose. Such electronegativity differences were first noted by Bols et al. in their studies of stereoelectronic effects on sugar glycosylation and Brønsted acidity [65]. Our group has subsequently demonstrated the impact of axial and equatorial fluorine substitution on the HB acidity of equatorial cyclohexanols (Figure 4B): the 1,3‐fluorohydrin **12** with the “W‐arrangement” is the strongest HB donor, with a HB acidity difference of the same magnitude as between **Glc2**, which also features a “W‐arrangement”, and **Gal2** [60].

The HB acidity differences between the other Glc‐Gal matched pairs were rationalised via comparison between their IR spectra (Figure 4C–E). The weaker HBDC of **Gal3** compared to **Glc3** is explained by the possibility of a *c*‐5‐IMHB between the Gal 3‐OH and 4‐OMe groups. However, given the explanation for the **Glc2** HBDC (see above), the observed decrease is comparatively minor. Indeed, the **Gal3** IR spectrum (Figure 4C) indicates that the *c*‐5‐IMHB (observed as a shoulder at 3571 cm^−1^) is not very abundant. This surprisingly low‐abundance of **Gal3‐*t*** is consistent with the observed ^3^ *J*_H3,OH_‐value (5.5 Hz), which is much lower than the ∼9 Hz expected in the case of a *cis*‐5‐IMHB arrangement. It is also consistent with the lower relative DFT‐calculated energy of **Gal3‐*g^+^*** compared to that of **Gal3‐*t*** (Δ*G* = +1.6 kJ/mol, see Supporting Information). Analysis of the conformational *V*_α_(r) values of the alcohol groups (see Supporting Information) shows that nevertheless the *V*_α_(r) value associated with the conformer with the *cis*‐5‐IMHB to 4‐OMe is smaller than that associated with the *tran*s‐5‐IMHB. This means that the latter is a weaker IMHB, and thus IMHB strength cannot be the reason for the high abundance of this conformer. However, the higher abundance of the conformer with the weaker 5‐IMHB is consistent with a smaller‐than‐expected decrease in p*K*_AHY_ value. The larger dipole moment of the **Gal3‐*t*** conformer (2.98 D) compared to the most stable **Gal3‐*g^+^*** (2.06 D) might further contribute to its destabilisation in CCl_4_.

The significantly weaker HBDC of the galactose 4‐OH group may be surprising given the much smaller proportion of 6‐ring IMHB compared to glucose 4‐OH, as deduced from the relative size of the absorption bands (Figure 4D) and analysis of the ^3^ *J*_H5‐H6_‐values (see Supporting Information). However, a key factor is the much‐reduced influence of the inductive effect, as the Gal 4‐OH group does not have an antiperiplanar disposition to the ring oxygen, nor does it have a “W‐arrangement” with the 2‐OMe group, in contrast to the Glc 4‐OH group. In addition, the Gal 4‐OH group is also able to establish a very strong *c*‐5‐IMHB, as evidenced by the lower wavenumber of the corresponding OH‐absorption band and its low *V*_α_(r) value. These observations are fully consistent with ^1^H NMR analysis, which indicates that the *c*‐5‐ring IMHB to 3‐OMe (**Gal4‐*g^+^***) significantly prevails over the possible *c*‐5‐ring IMHB to the ring oxygen (**Gal4‐*t*)**, as evidenced by the very low observed ^3^ *J*_OH‐H4_‐value (1.2 Hz). Furthermore, the high population of the **Gal4‐*g^+^*** conformation is nicely revealed by a ^4^ *J* ‘W‐coupling’ of the alcohol proton with H5 (also 1.2 Hz) [30, 66]. This is also in full agreement with the DFT‐calculated relative energy of **Gal4‐*t*** being 9.6 kJ mol^−1^ higher compared to that of **Gal4‐*g^+^*** (see Supporting Information). The conformer with the 6‐IMHB is even more destabilised (by 13.0 kJmol^−1^). Hence, the combination of a much‐reduced inductive effect with a strong IMHB results in the very low HBDC of **Gal4**.

The Gal 6‐OH group is a much better HB donor compared to the Glc 6‐OH, despite the IR spectrum revealing a much larger proportion of 6‐IMHB (Figure 4E). The comparatively weaker *c*‐5‐IMHB to O5 is not expected to be important, as this motif, which requires a *gg*‐conformation, is also subject to dipole repulsion, leaving the weak *t*‐5‐IMHB (*gt*‐conformation) as the only competing IMHB. However, the IR spectrum of **Gal6** reveals the presence of a free hydroxyl group (OH absorption band at 3644 cm^−1^). Analysis of the ^3^ *J*_H5‐H6_‐values (^1^H NMR spectrum in dilute CCl_4_ solution) using the Crich equations [64] in combination with the ^3^ *J*_H,OH_‐values, indeed indicates a large *tg*‐population (28%), for which there is no IMHB possible. The deduced populations of the exocyclic hydroxymethyl group are qualitatively consistent with the IR spectrum. The DFT calculation data fully correspond with this analysis, with the *gt*‐conformer being the most stable, followed by the *tg‐* and *gg*‐conformers, which are respectively 5.2 and 5.7 kJ/mol higher than the most stable *gt* conformer (see Supporting Information). Hence, the presence of a free OH group is proposed to be responsible for the enhanced OH acidity. Not only is there no competition from IMHB, but this OH group is also subject to a strong inductive effect as its *tg*‐conformation makes it antiperiplanar with O5, resulting in a large *V*_α_(r) value which, being a free alcohol, correlates with HB acidity [54, 58]. The same analysis in CDCl_3_ leads to very similar conformational populations (see Supporting Information).

Our findings regarding the Glc 4‐OH and Gal 6‐OH being the strongest HB donors is in line with the observation that in singly hydrated complexes, water accepts 4‐OH in Glc and 6‐OH in Gal [39].

### 2.4 Comparison With Brønsted Acidities

**FIGURE 5:**
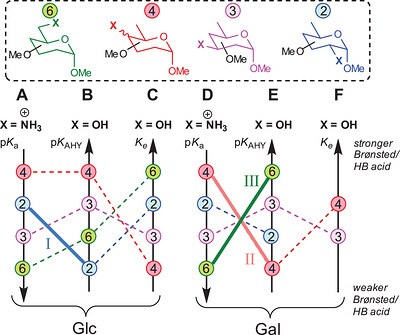
Comparison of the p*K*_AHY_‐values (B, E) with the Pedersen/Bols p*K*_a_‐values of per‐*O*‐methylated aminosugars (A, D) [44] and the Thiem *K*_e_‐values (equilibrium constants of sugar alcohols with NaO*^i^* Pr in *^i^* PrOH (C, F) [43]. Data shown not to scale.

The availability of p*K*_a_‐data at the individual glucose and galactose positions invites a comparison with the obtained HB acidities (Figure 5). In a study aimed at the quantification of the electronic effects of carbohydrate alcohol groups, Pedersen and Bols have reported the Brønsted acidities of per‐*O*‐methylated gluco‐ and galactosammonium derivatives (Figure 5A,D) [44]. In both cases, the 4‐ammonium derivatives are the strongest acids, followed by the 2‐ammonium derivatives. This is consistent with an inductive effect from the ring oxygen. Next are the 3‐ammonium and the 6‐ammonium derivatives, with the latter having only one electronegative substituent. Interestingly, the Thiem *K*_e_‐values (Figure 5C,F), which are equilibrium constants of sugar alcohols with NaO*^i^* Pr in *^i^* PrOH, do show a different ranking (compare Figure 5A with 5C), which must originate from different factors stabilising sodium alcoholate salts in *^i^* PrOH compared to ammonium groups in water.

The HBDC ranking for the glucose derivatives is the same as the Bols glucosammonium ranking (compare Figure 5A,B), except for **GlcN2**, which has a comparatively larger Brønsted acidity than the HB acidity of **Glc2**. For the galactose derivatives (Figure 5D,E), the order of HBDC is opposite to that of Brønsted acidity. The HBDC rankings do not correspond with Thiem's rankings (Figure 5B,C,E,F).

It is well‐known that Brønsted and HB acidities do not always correlate, as the factors that stabilise charged species in aqueous medium are not the same as the factors that influence the HB equilibrium. Inductive effects play an important and parallel role in promoting both Brønsted and HB acidities, but in the case of sugars, intramolecular hydrogen bonding has a much larger effect. This would explain why **Glc2** is a comparatively worse HB donor than **GlcN2** is a Bronsted acid (strong *cis*‐5‐ring IMHB with anomeric OMe), and **Gal4** (strong IMHB to 3‐OMe) a worse HB donor than **GalN4**)(see lines I and II in Figure 5). **GalN6** is the weakest acid as it only has one electron vicinal withdrawing group, but **Gal6** is the best HB donor due to the significant population of *tg*‐conformer (line III). In addition (not shown), the much greater difference in p*K*_AHY_ between **Glc4** and **Gal4** compared to the p*K*_a(H)_ difference between **GlcN4** and **GalN4**, is explained by the involvement of Gal4‐OH in *cis‐*5‐ring IMHB with 3‐OMe.

## 3 Conclusion

A capping approach allowed the measurement of the HB donating capacity of individual methyl α‐glucoside and α‐galactoside hydroxyl groups and enabled their ranking on the p*K*_AHY_‐hydrogen bond scale, which, to the best of our knowledge, is the first such ranking for sugar alcohols. The differences in HB donating capacities between sugar alcohols could be rationalised by a combination of inductive effects, intramolecular hydrogen bonding and exocyclic hydroxymethyl conformation, and showed measurable differences between the glucose and, notably, the galactose alcohol groups. Key aspects include:

‐ The Glc 4‐OH group is antiperiplanar to the ring oxygen, significantly increasing its HB acidity.
‐ The presence of IMHB leads to a decrease in HB acidity. *Cis*‐5‐ring “hydrindane‐like” IMHB are typically stronger than their trans analogues, and IMHB to the anomeric oxygens are weaker. Notable examples involve **Gal4**‐OH (*cis*‐5‐ring IMHB with 3‐OMe), and also **Glc2** and **Gal2** (*cis*‐5‐ring IMHB with anomeric OMe).
‐ IMHB involving Gal6‐OH tends to be stronger compared to Glc6‐OH.
‐ The presence of a “W‐arrangement” as in 1,3‐diequatorial 3‐methoxy alcohols, exerts a stronger inductive effect compared to a situation where one of the groups is axial (**Glc2** vs. **Gal2**), leading to a measurable difference in HB acidities.
‐ The presence of free alcohol groups lead to a HB acidity increase, a notable example is **Gal6**.
‐ Except for **Gal6**, the alcohol groups of the sugar derivatives measured here have a lower HB acidity than those of cyclohexanol (axial or equatorial OH), but all Glc and Gal sugar alcohols display higher HB acidity compared to the 2‐methoxy cyclohexanols.
‐ The H‐bond acidities of the sugar alcohols do not correlate well with their Bronsted acidities. Inductive effects play an important and parallel role in promoting both Brønsted and HB acidities, but we suggest that for the latter, intramolecular hydrogen bonding has a much larger effect.

It is hoped that this knowledge of innate sugar alcohol HB donating capacities will allow an improved interpretation of protein‐carbohydrate interactions and will allow improved rational design of carbohydrate‐based probes and glycomimetics. Studies on modified carbohydrates are in progress and will be reported in due course.

## Author Contributions

**Mrinal Naskar**: writing – review and editing, data curation, methodology, investigation, formal analysis. **Zhong Wang**: investigation, formal analysis. **Krunal Patel**: investigation. **Branko De Baets**: investigation. **Anaïs Goupille**: investigation, formal analysis. **Davy Sinnaeve**: investigation. **Eric Renault**: investigation, methodology, writing – review and editing, formal analysis, supervision, data curation. **Jean‐Yves Le Questel**: investigation, methodology, writing – review and editing, formal analysis, supervision, data curation, project administration, funding acquisition. **Bruno Linclau**: conceptualization, funding acquisition, writing – original draft, writing – review and editing, formal analysis, supervision, project administration, data curation, methodology.

## Conflicts of Interest

The authors declare no conflicts of interest.

## Supporting information



The authors have cited additional references within the Supporting Information [67, 68, 69, 70, 71, 72, 73, 74, 75, 76, 77, 78, 79, 80, 81, 82, 83, 84, 85].**Supporting File**: anie73124‐sup‐0001‐SuppMat.pdf.

## Data Availability

The data that supports the findings of this study are available in the Supporting Information of this article.

## References

[1] T. Steiner , “The Hydrogen Bond in the Solid State,” Angewandte Chemie International Edition 41 (2002): 48–76, 10.1002/1521-3773(20020104)41:1<48::AID-ANIE48>3.0.CO;2-U. 12491444

[2] S. J. Grabowski , “What Is the Covalency of Hydrogen Bonding?” Chemical Reviews 111 (2011): 2597–2625, 10.1021/cr800346f. 21322583

[3] D. Herschlag and M. M. Pinney , “Hydrogen Bonds: Simple After All?” Biochemistry 57 (2018): 3338–3352, 10.1021/acs.biochem.8b00217. 29678112

[4] E. Arunan , G. R. Desiraju , R. A. Klein , et al., “Defining the Hydrogen Bond: An Account (IUPAC Technical Report),” Pure and Applied Chemistry 83 (2011): 1619–1636, 10.1351/PAC-REP-10-01-01.

[5] S. C. C. van der Lubbe and C. F. Guerra , “The Nature of Hydrogen Bonds: A Delineation of the Role of Different Energy Components on Hydrogen Bond Strengths and Lengths,” Chemistry ‐ An Asian Journal 14 (2019): 2760–2769. 31241855 10.1002/asia.201900717 PMC6771679

[6] S. Shaik , D. Danovich , and R. N. Zare , “Valence Bond Theory Allows a Generalized Description of Hydrogen Bonding,” Journal of the American Chemical Society 145 (2023): 20132–20140, 10.1021/jacs.3c08196. 37664980 PMC10510329

[7] G. Bulusu and G. R. Desiraju , “Strong and Weak Hydrogen Bonds in Protein–Ligand Recognition,” Indian Institute of Science 100 (2019): 31–41, 10.1007/s41745-019-00141-9.

[8] M. H. Abraham , H. S. Chadha , G. S. Whiting , and R. C. Mitchell , “Hydrogen Bonding. 32. An Analysis of Water‐Octanol and Water‐Alkane Partitioning and the Δlog P Parameter of Seiler,” Journal of Pharmaceutical Sciences 83 (1994): 1085–1100, 10.1002/jps.2600830806. 7983591

[9] O. A. Raevsky , “Physicochemical Descriptors in Property‐Based Drug Design,” Mini ‐ Reviews in Medicinal Chemistry 4 (2004): 1041–1052, 10.2174/1389557043402964. 15579112

[10] B. Kuhn , P. Mohr , and M. Stahl , “Intramolecular Hydrogen Bonding in Medicinal Chemistry,” Journal of Medicinal Chemistry 53 (2010): 2601–2611, 10.1021/jm100087s. 20175530

[11] G. Caron , J. Kihlberg , and G. Ermondi , “Intramolecular Hydrogen Bonding: An Opportunity for Improved Design in Medicinal Chemistry,” Medicinal Research Reviews 39 (2019): 1707–1729, 10.1002/med.21562. 30659634

[12] G. A. Jeffrey and W. Saenger , Experimental Studies of Hydrogen Bonding in Hydrogen Bonding in Biological Structures, (Springer, Berlin, Heidelberg, 1991): 169–219, 10.1007/978-3-642-85135-3.

[13] J. L. Dashnau , K. A. Sharp , and J. M. Vanderkooi , “Carbohydrate Intramolecular Hydrogen Bonding Cooperativity and Its Effect on Water Structure,” Journal of Physical Chemistry B 109 (2005): 24152–24159, 10.1021/jp0543072. 16375407

[14] M. M. Deshmukh , L. J. Bartolotti , and S. R. Gadre , “Intramolecular Hydrogen Bonding and Cooperative Interactions in Carbohydrates via the Molecular Tailoring Approach,” Journal of Physical Chemistry A 112 (2008): 312–321, 10.1021/jp076316b. 18085757

[15] W. I. Weis and K. Drickamer , “Structural Basis of Lectin‐Carbohydrate Recognition,” Annual Review of Biochemistry 65 (1996): 441–473, 10.1146/annurev.bi.65.070196.002301. 8811186

[16] L. Gajdos , M. P. Blakeley , A. Kumar , et al., “Visualization of Hydrogen Atoms in a Perdeuterated Lectin‐Fucose Complex Reveals Key Details of Protein‐Carbohydrate Interactions,” Structure 29 (2021): 1003–1013.e4, 10.1016/j.str.2021.03.003. 33765407

[17] A. M. Ruvinsky , I. Aloni , D. Cappel , et al., “The Role of Bridging Water and Hydrogen Bonding as Key Determinants of Noncovalent Protein–Carbohydrate Recognition,” ChemMedChem 13 (2018): 2684–2693, 10.1002/cmdc.201800437. 30380198

[18] J. Dong and A. P. Davis , “Molecular Recognition Mediated by Hydrogen Bonding in Aqueous Media,” Angewandte Chemie 60 (2021): 8035–8048, 10.1002/anie.202012315. 33075210

[19] A. M. Keys , D. W. Kastner , L. L. Kiessling , and H. J. Kulik , “CH−π Interactions Confer Orientational Flexibility in Protein–carbohydrate Binding Sites,” Journal of Biological Chemistry 301 (2025): 110379, 10.1016/j.jbc.2025.110379. 40523618 PMC12309604

[20] A. M. Keys , D. W. Kastner , L. L. Kiessling , and H. J. Kulik , “The Energetic Landscape of CH–π Interactions in Protein–Carbohydrate Binding,” Chemical Science 16 (2025): 1746–1761, 10.1039/D4SC06246A. 39669175 PMC11632809

[21] C. B. Barnett and K. J. Naidoo , “Stereoelectronic and Solvation Effects Determine Hydroxymethyl Conformational Preferences in Monosaccharides,” Journal of Physical Chemistry B 112 (2008): 15450–15459, 10.1021/jp8067409. 18989909

[22] E. Hatcher , E. Säwén , G. Widmalm , and A. D. MacKerell Jr. , “Conformational Properties of Methyl β‐Maltoside and Methyl α‐ and β‐Cellobioside Disaccharides,” Journal of Physical Chemistry B 115 (2011): 597–608, 10.1021/jp109475p. 21158455 PMC3077104

[23] J. Rönnols , O. Engström , U. Schnupf , E. Säwén , J. W. Brady , and G. Widmalm , “Inter‐Residual Hydrogen Bonding in Carbohydrates Unraveled by NMR Spectroscopy and Molecular Dynamics Simulations,” ChemBioChem 20 (2019): 2519–2528, 10.1002/cbic.201900301. 31066963

[24] N. Yadav , S. Djalali , A. Poveda , et al., “Dissecting the Conformational Stability of a Glycan Hairpin,” Journal of the American Chemical Society 146 (2024): 6369–6376, 10.1021/jacs.4c00423. 38377472 PMC10921397

[25] Y. Yu , T. Tyrikos‐Ergas , Y. Zhu , et al., “Systematic Hydrogen‐Bond Manipulations to Establish Polysaccharide Structure–Property Correlations,” Angewandte Chemie International Edition 58 (2019): 13127–13132, 10.1002/anie.201906577. 31359577 PMC6772130

[26] G. Fittolani , S. Djalali , M. A. Chaube , et al., “Deoxyfluorination Tunes the Aggregation of Cellulose and Chitin Oligosaccharides and Highlights the Role of Specific Hydroxyl Groups in the Crystallization Process,” Organic & Biomolecular Chemistry 20 (2022): 8228–8235, 10.1039/D2OB01601J. 36254595

[27] V. Dimakos and M. S. Taylor , “Site‐Selective Functionalization of Hydroxyl Groups in Carbohydrate Derivatives,” Chemical Reviews 118 (2018): 11457–11517, 10.1021/acs.chemrev.8b00442. 30507165

[28] H. Hönig and H. Weidmann , “Zur Zuordnung Intramolekularer Wasserstoffbrücken in Hexopyranosen,” Carbohydrate Research 73 (1979): 260–266.

[29] H. Spedding , “705. Intramolecular Hydrogen Bonding in Methyl 4,6‐O‐benzylidene‐D‐Aldohexosides,” Journal of the Chemical Society (Resumed) 1961 (1961): 3617, 10.1039/jr9610003617.

[30] P. R. Muddasani , E. Bozó , B. Bernet , and A. Vasella , “Glycosylidene Carbenes. Part 14. Glycosidation of Partially Protected Galactopyranose‐, Glucopyranose‐, and Mannopyranose‐Derived Vicinal Diols,” Helvetica Chimica Acta 77 (1994): 257–290, 10.1002/hlca.19940770128.

[31] N. L. Allinger and J. L. Coke , “The Relative Stabilities of Cis and Trans Isomers. VII. The Hydrindanes^1,2^ ,” Journal of the American Chemical Society 82 (1960): 2553–2556, 10.1021/ja01495a039.

[32] A. J. Michell and H. G. Higgins , “Conformation and Intramolecular Hydrogen Bonding in Glucose and Xylose Derivatives,” Tetrahedron 21 (1965): 1109–1120, 10.1016/0040-4020(65)80053-9.

[33] J. Bartsch and V. Prey , “Intramolekulare Wasserstoffbrücken in Substituierten Gluco‐ Bzw. Fructopyranosen und ‐Furanosen,” Justus Liebigs Annalen der Chemie 717 (1968): 198–204, 10.1002/jlac.19687170121.

[34] A. J. Michell and H. G. Higgins , “The Absence of Free Hydroxyl Groups in Cellulose,” Cellulose 6 (1999): 89–91, 10.1023/A:1009258732505.

[35] N. Dominelli‐Whiteley , J. J. Brown , K. B. Muchowska , et al., “Strong Short‐Range Cooperativity in Hydrogen‐Bond Chains,” Angewandte Chemie International Edition 56 (2017): 7658–7662, 10.1002/anie.201703757. 28493462 PMC5488241

[36] M. L. de la Paz , G. Ellis , M. Perez , J. Perkins , J. Jimenez‐Barbero , and C. Vicent , “Carbohydrate Hydrogen‐Bonding Cooperativity− Intramolecular Hydrogen Bonds and Their Cooperative Effect on Intermolecular Processes− Binding to a Hydrogen‐Bond Acceptor Molecule,” European Journal of Organic Chemistry 2002 (2002): 840–855.

[37] J. S. Lomas and L. Joubert , “On the Importance of Intramolecular Hydrogen Bond Cooperativity in D ‐Glucose—An NMR and QTAIM Approach,” Magnetic Resonance in Chemistry 55 (2017): 893–901, 10.1002/mrc.4599. 28432857

[38] L. Trevisan , A. D. Bond , and C. A. Hunter , “Quantitative Measurement of Cooperativity in H‐Bonded Networks,” Journal of the American Chemical Society 144 (2022): 19499–19507, 10.1021/jacs.2c08120. 36223562 PMC9619404

[39] P. Çarçabal , R. A. Jockusch , I. Hünig , et al., “Hydrogen Bonding and Cooperativity in Isolated and Hydrated Sugars: Mannose, Galactose, Glucose, and Lactose,” Journal of the American Chemical Society 127 (2005): 11414–11425, 10.1021/ja0518575. 16089470

[40] J. P. Simons , B. G. Davis , E. J. Cocinero , D. P. Gamblin , and E. C. Stanca‐Kaposta , “Conformational Change and Selectivity in Explicitly Hydrated Carbohydrates,” Tetrahedron‐Asymmetry 20 (2009): 718–722, 10.1016/j.tetasy.2009.02.032.

[41] N. Varga , M. Smiesko , X. Jiang , et al., “Strengthening an Intramolecular Non‐Classical Hydrogen Bond to Get in Shape for Binding,” Angewandte Chemie *International Edition* 63 (2024): e202406024. 39072885 10.1002/anie.202406024

[42] C. W. Chang , M. H. Lin , C. K. Chan , et al., “Automated Quantification of Hydroxyl Reactivities: Prediction of Glycosylation Reactions,” Angewandte Chemie *International Edition* 60 (2021): 12413–12423, 10.1002/anie.202013909. 33634934

[43] M. Matwiejuk and J. Thiem , “Hydroxy Group Acidities of Partially Protected Glycopyranosides,” European Journal of Organic Chemistry 2012 (2012): 2180–2187, 10.1002/ejoc.201101708.

[44] C. M. Pedersen , J. Olsen , A. B. Brka , and M. Bols , “Quantifying the Electronic Effects of Carbohydrate Hydroxy Groups by Using Aminosugar Models,” Chemistry – A European Journal 17 (2011): 7080–7086, 10.1002/chem.201100020. 21542038

[45] R. Hevey , “Strategies for the Development of Glycomimetic Drug Candidates,” Pharmaceuticals 12 (2019): 55, 10.3390/ph12020055. 30978966 PMC6631974

[46] X. Cao , X. Du , H. Jiao , et al., “Carbohydrate‐Based Drugs Launched During 2000− 2021,” Acta Pharmaceutica Sinica B 12 (2022): 3783–3821. 36213536 10.1016/j.apsb.2022.05.020 PMC9532563

[47] S. Leusmann , P. Ménová , E. Shanin , A. Titz , and C. Rademacher , “Glycomimetics for the Inhibition and Modulation of Lectins,” Chemical Society Reviews 52 (2023): 3663–3740, 10.1039/D2CS00954D. 37232696 PMC10243309

[48] A. Tamburrini , C. Colombo , and A. Bernardi , “Design and Synthesis of Glycomimetics: Recent Advances,” Medicinal Research Reviews 40 (2020): 495–531, 10.1002/med.21625. 31361344

[49] L. Yuan , Y. Hua , and X. Wang , “Recent Progress of Glycomimetics in Drug Development,” Organic & Biomolecular Chemistry 23 (2025): 7671–7680, 10.1039/D5OB00850F. 40685809

[50] R. J. Young , “Today's Drug Discovery and the Shadow of the Rule of 5,” Expert Opinion on Drug Discovery 18 (2023): 965–972, 10.1080/17460441.2023.2228199. 37378429

[51] J. S. Scott and M. J. Waring , “Practical Application of Ligand Efficiency Metrics in Lead Optimisation,” Bioorganic & Medicinal Chemistry 26 (2018): 3006–3015, 10.1016/j.bmc.2018.04.004. 29655612

[52] C. Hansch , J. P. Björkroth , and A. Leo , “Hydrophobicity and Central Nervous System Agents: On the Principle of Minimal Hydrophobicity in Drug Design,” Journal of Pharmaceutical Sciences 76 (1987): 663–687, 10.1002/jps.2600760902. 11002801

[53] P. W. Kenny , “Hydrogen‐Bond Donors in Drug Design,” Journal of Medicinal Chemistry 65 (2022): 14261–14275, 10.1021/acs.jmedchem.2c01147. 36282210

[54] J. Graton , Z. Wang , A.‐M. Brossard , D. Gonçalves Monteiro , J.‐Y. Le Questel , and B. Linclau , “An Unexpected and Significantly Lower Hydrogen‐Bond‐Donating Capacity of Fluorohydrins Compared to Nonfluorinated Alcohols,” Angewandte Chemie International Edition 51 (2012): 6176–6180, 10.1002/anie.201202059. 22577052 PMC3601419

[55] S. J. Grabowski , “Hydrogen Bond and Other Lewis Acid–Lewis Base Interactions as Preliminary Stages of Chemical Reactions,” Molecules 25 (2020): 4668. 33066201 10.3390/molecules25204668 PMC7587390

[56] P. Muller , “Glossary of Terms Used in Physical Organic Chemistry (IUPAC Recommendations 1994),” Pure and Applied Chemistry 66 (1994): 1077–1184, 10.1351/pac199466051077.

[57] B. Linclau , J. Graton , and J.‐Y. Le Questel , “8 ‐ Influence of Fluorination on Alcohol Hydrogen‐Bond Donating Properties,” in Fluorine in Life Sciences: Pharmaceuticals, Medicinal Diagnostics, and Agrochemicals, edited by G. Haufe and F. R. Leroux (Academic Press, 2019), 301–324.

[58] J. Graton , F. Besseau , A.‐M. Brossard , E. Charpentier , A. Deroche , and J.‐Y. Le Questel , “Hydrogen‐Bond Acidity of OH Groups in Various Molecular Environments (Phenols, Alcohols, Steroid Derivatives, and Amino Acids Structures): Experimental Measurements and Density Functional Theory Calculations,” Journal of Physical Chemistry A 117 (2013): 13184–13193, 10.1021/jp410027h. 24274054

[59] P. W. Kenny , “Hydrogen Bonding, Electrostatic Potential, and Molecular Design,” Journal of Chemical Information and Modeling 49 (2009): 1234–1244, 10.1021/ci9000234. 19382744

[60] Z. Wang , G. Compain , M. Naskar , et al., “The Conformational Electrostatic Potential as Predictor for the Influence of Fluorination on Alcohol Hydrogen Bonding: Identification of a CF_2_ H‐Mediated Cooperative Effect,” ChemistryEurope 4 (2026): e202500203, 10.1002/ceur.202500203.

[61] J. A. K. Howard , V. J. Hoy , D. O'Hagan , and G. T. Smith , “How Good Is Fluorine as a Hydrogen Bond Acceptor?,” Tetrahedron 52 (1996): 12613–12622, 10.1016/0040-4020(96)00749-1.

[62] C. Dalvit and A. Vulpetti , “Intermolecular and Intramolecular Hydrogen Bonds Involving Fluorine Atoms: Implications for Recognition, Selectivity, and Chemical Properties,” Chemistry and Medicinal Chemistry 7 (2012): 262–272, 10.1002/cmdc.201100483. 22262517

[63] J. Gonzalez‐Outeiriño , K. N. Kirschner , S. Thobhani , and R. J. Woods , “Reconciling Solvent Effects on Rotamer Populations in Carbohydrates – A Joint MD and NMR Analysis,” Canadian Journal of Chemistry 84 (2006): 569–579, 10.1139/v06-036. 25544777 PMC4276422

[64] H. Amarasekara , S. Dharuman , T. Kato , and D. Crich , “Synthesis of Conformationally‐Locked Cis—and Trans ‐Bicyclo[4.4.0] Mono‐, Di‐, and Trioxadecane Modifications of Galacto‐ and Glucopyranose; Experimental Limiting^3^ _H,H_ Coupling Constants for the Estimation of Carbohydrate Side Chain Populations and beyond,” Journal of Organic Chemistry 83 (2018): 881–897, 10.1021/acs.joc.7b02891. 29241001 PMC5775050

[65] M. Bols , X. F. Liang , and H. H. Jensen , “Equatorial Contra Axial Polar Substituents. The Relation of a Chemical Reaction to Stereochemical Substituent Constants,” Journal of Organic Chemistry 67 (2002): 8970–8974, 10.1021/jo0205356. 12467416

[66] E. A. Larsson , J. Ulicny , A. Laaksonen , and G. Widmalm , “Analysis of NMR J Couplings in Partially Protected Galactopyranosides,” Organic Letters 4 (2002): 1831–1834, 10.1021/ol0200347. 12027625

[67] C. Laurence , K. A. Brameld , J. Graton , J. Y. Le Questel , and E. Renault , “The p _BHX_ Database: Toward a Better Understanding of Hydrogen‐Bond Basicity for Medicinal Chemists,” Journal of Medicinal Chemistry 52 (2009): 4073–4086, 10.1021/jm801331y. 19537797

[68] S. A. Barker , J. S. Brimacombe , A. B. Foster , D. H. Whiffen , and G. Zweifel , “Aspects of Stereochemistry—II,” Tetrahedron 7 (1959): 10–18, 10.1016/0040-4020(59)80046-6.

[69] S. J. Angyal and R. M. Hoskinson , “578. Cyclitols. Part XIII. The Conformation of Cyclic Diketals: The Skew Conformation,” Journal of the Chemical Society (Resumed) 1962 (1962): 2991, 10.1039/jr9620002991.

[70] A. R. H. Cole , G. T. A. Müller , D. W. Thornton , and R. L. S. Willix , “240. Infrared Spectra of Natural Products. Part IX. Frequencies and Intensities of Hydroxyl Absorption Bands in Triterpenoids and Similar Compounds,” Journal of the Chemical Society (Resumed) (1959): 1218–1222, 10.1039/JR9590001218.

[71] L. P. Kuhn , “The Hydrogen Bond. II. The Intramolecular Bond in Cyclic 1,2‐Diols,” Journal of the American Chemical Society 76 (1954): 4323–4326, 10.1021/ja01646a023.

[72] L. P. Kuhn , “The Hydrogen Bond. I. Intra‐ and Intermolecular Hydrogen Bonds in Alcohols,” Journal of the American Chemical Society 74 (1952): 2492–2499, 10.1021/ja01130a013.

[73] M. J. Frisch , G. W. Trucks , H. B. Schlegel , et al., Gaussian, Inc., Wallingford CT, (2016).

[74] M. Bursch , J. M. Mewes , A. Hansen , and S. Grimme , “Best‐Practice DFT Protocols for Basic Molecular Computational Chemistry**,” Angewandte Chemie *International Edition* 61 (2022): e202205735, 10.1002/anie.202205735. 36103607 PMC9826355

[75] A. V. Marenich , C. J. Cramer , and D. G. Truhlar , “Universal Solvation Model Based on Solute Electron Density and on a Continuum Model of the Solvent Defined by the Bulk Dielectric Constant and Atomic Surface Tensions,” Journal of Physical Chemistry B 113 (2009): 6378–6396, 10.1021/jp810292n. 19366259

[76] R. E. Rosenberg , R. L. Abel , M. D. Drake , et al., “An Examination of Hyperconjugative and Electrostatic Effects in the Hydride Reductions of 2‐Substituted‐4‐ tert ‐butylcyclohexanones,” Journal of Organic Chemistry 66 (2001): 1694–1700, 10.1021/jo0011787. 11262115

[77] M. Matwiejuk and J. Thiem , “Defining Oxyanion Reactivities in Base‐promoted Glycosylations,” Chemical Communications 47 (2011): 8379, 10.1039/c1cc11690h. 21701750

[78] V. Cachatra , A. Almeida , J. Sardinha , et al., “Wittig Reaction: Domino Olefination and Stereoselectivity DFT Study. Synthesis of the Miharamycins′ Bicyclic Sugar Moiety,” Organic Letters 17 (2015): 5622–5625, 10.1021/acs.orglett.5b02849. 26551053

[79] R. Kaifu , L. C. Plantefaber , and I. J. Goldstein , “2‐Substituted Methyl α‐d‐Galactopyranosides: Synthesis and Binding Affinity for the A and B Subunits of the Griffonia Simplicifolia I Isolectins,” Carbohydrate Research 140 (1985): 37–49, 10.1016/0008-6215(85)85047-3. 4053097

[80] P. L. Barili , G. Berti , D. Bertozzi , et al., “Meta‐chloroperbenzoic Acid as a Selective Reagent for the Removal of O‐Propenyl Groups. Its Use in the Synthesis of Some D‐galactopyranoside and 4‐Deoxy‐l‐Threo‐4‐Hexenopyranoside Derivatives,” Tetrahedron 46 (1990): 5365–5376, 10.1016/S0040-4020(01)87843-1.

[81] J. C. Lewis , S. Bastian , C. S. Bennett , et al., “Chemoenzymatic Elaboration of Monosaccharides Using Engineered Cytochrome P450_BM3_ Demethylases,” Proceedings of the National Academy of Sciences 106 (2009): 16550–16555, 10.1073/pnas.0908954106. PMC2757845 19805336

[82] M. Moumé‐Pymbock , T. Furukawa , S. Mondal , and D. Crich , “Probing the Influence of a 4,6‐ O ‐Acetal on the Reactivity of Galactopyranosyl Donors: Verification of the Disarming Influence of the Trans–Gauche Conformation of C5–C6 Bonds,” Journal of the American Chemical Society 135 (2013): 14249–14255, 10.1021/ja405588x. 23984633 PMC3814037

[83] D. Sinnaeve , M. Foroozandeh , M. Nilsson , and G. A. Morris , “A General Method for Extracting Individual Coupling Constants From Crowded^1^ H NMR Spectra,” Angewandte Chemie International Edition 55 (2016): 1090–1093, 10.1002/anie.201508691. 26636773 PMC4736434

[84] D. Sinnaeve , J. Ilgen , M. E. Di Pietro , et al., “Probing Long‐Range Anisotropic Interactions: A General and Sign‐Sensitive Strategy to Measure^1^ H–^1^ H Residual Dipolar Couplings as a Key Advance for Organic Structure Determination,” Angewandte Chemie International Edition 59 (2020): 5316–5320, 10.1002/anie.201915278. 31945235

[85] F. X. Cantrelle , E. Boll , and D. Sinnaeve , “Making^1^ H–^1^ H Couplings More Accessible and Accurate With Selective 2DJ NMR Experiments Aided by^13^ C Satellites,” Analytical Chemistry 96 (2024): 7056–7064, 10.1021/acs.analchem.4c00315. 38666447

