# Supplementary material for: Carbohydrate Physicochemical Properties: The Innate Hydrogen Bond Donating Capacities of α‐Glucoside and α‐Galactoside Alcohol Groups
Source: Angew Chem Int Ed Engl. 2026 Jun 16;65(34):e3574068. doi: 10.1002/anie.3574068 (PMC13480839; doi:10.1002/anie.3574068)
Supplement: Supplementary file 1 [file ANIE-65-e3574068-s001.pdf]

SUPPORTING INFORMATION

Carbohydrate physicochemical properties: the innate hydrogen bond donating capacities of α-glucoside and α-galactoside alcohol groups

Mrinal Naskar,[a] Zhong Wang,[b] Krunal Patel,[b] Branko De Baets,[a] Anaïs Goupille,[c] Davy Sinnaeve,[d] Eric Renault,[c] Jean-Yves Le Questel,[c]* and Bruno Linclau*[a,b],

[a] Prof Dr B. Linclau, Mrinal Naskar, Branko De Baets
Department of Organic and Macromolecular Chemistry, Ghent University, Campus Sterre, S4, Krijgslaan 281, B-9000 Ghent, Belgium
E-mail: Bruno.linclau@ugent.be

[b] Prof Dr B. Linclau, Dr Zhong Wang, Krunal Patel
School of Chemistry and Chemical Engineering, University of Southampton, Highfield, Southampton SO171BJ, UK

[c] Prof Dr Jean-Yves Le Questel, Dr Eric Renault, Anaïs Goupille
CEISAM UMR CNRS 6230, Nantes Université, CNRS, CEISAM UMR 6230, F-44000 Nantes, France
E-mail: jean-yves.le-questel@univ-nantes.fr

[d] Dr Davy Sinnaeve,
CNRS, Univ. Lille, Institut Pasteur de Lille, UMR 9031 — Integrative Structural Biology, F-59000, Lille, France.

Table of contents

Contents

1 HB acidity measurement 4
1.1 FT-IR method 4
1.1.1 HB acidity/donating capacity scale pK_{AHY} 4
1.1.2 General Experimental Procedure 5
1.1.3 Sample Preparation 5
1.2 IR experimental data (Table S1) 7
1.3 IR Spectra 8
1.3.1 Comparison IR spectra 8
1.3.2 Model cyclohexanol derivatives 8
1.3.3 Glucose derivatives 9
1.3.4 Galactose derivatives 9
1.3.5 Individual IR spectra (Table S2) 10
2 Determination of the exocyclic hydroxylmethyl group conformational populations 14
2.1 Glc6 ($CDCl_3$) 14
2.2 Gal6 (CCl_4) 15
2.3 Gal6 ($CDCl_3$) 16
2.4 Gal4 ($CDCl_3$) 16
2.5 Gal3 ($CDCl_3$) 17
3 DFT calculations 18
3.1 Table S3. 3D-structures of the different major conformers of Glc2 in CCl_4. 19
3.2 Table S4. 3D-structures of the different major conformers of Gal3 in CCl_4. 20
3.3 Table S5. 3D-structures of the different major conformers of Gal4 in CCl_4. 21

3.4 Table S6. 3D-structures of the different major conformers of Gal6 in CCl_4. ... 22

3.5 Table S7. 3D-structures of the different major conformers of Glc6 in CCl_4. ... 23

3.6 Structural coordinates, total energies and dipole moment for Glc 2, Gal3, Gal4, Gal6 and Glc6. 24

3.6.1 Glc2_3: Glc2-(*g*+)-(5-*c*-IMHB)[a,b] ... 24

3.6.2 Glc2_1: Glc2-(*t*)-(5-*c*-IMHB)[a,b] ... 24

3.6.3 Glc2_2: Glc2-(*g*+)-(5-*t*-IMHB)[a,b] ... 25

3.6.4 Glc2_4: Glc2-(*t*)-(5-*t*-IMHB)[a,b] ... 25

3.6.5 Gal3_1: Gal3-(*g*+)-(5-*t*-IMHB)[a,b] ... 26

3.6.6 Gal3_2: Gal3-(*t*)-(5-*c*-IMHB)[a,b] ... 26

3.6.7 Gal4_1: Gal4-(*g*+)-(5-*c*-IMHB)-(*gt*)[a,b,c] ... 27

3.6.8 Gal4_2: Gal4-(*t*)-(5-*c*-IMHB)-(*gt*)[a,b,c] ... 27

3.6.9 Gal4_3: Gal4-(*g*-)-(6-IMHB)-(*gg*)[a,c] ... 28

3.6.10 Gal6_1: Gal6-(*g*-)-(5-IMHB)-(*gt*)[c,d] ... 29

3.6.11 Gal6_2: Gal6-(*g*+)-free-(*tg*)[c,d] ... 29

3.6.12 Gal6_3: Gal6-(*g*-)-(6-ring)-(*gg*)[c,d] ... 30

3.6.13 Gal6_4: Gal6-(*g*-)-free-(*tg*)[c,d] ... 30

3.6.14 Glc6_1: Glc6-(*g*+)-(5-IMHB)-(*gg*)[c,d] ... 31

3.6.15 Glc6_2: Glc6-(*g*+)-free-(*gt*)[c,d] ... 31

3.6.16 Glc6_3: Glc6-(*g*+)-(6-IMHB)-(*tg*)[c,d] ... 32

4 Synthesis ... 33

4.1 General Methods. ... 33

4.2 Synthesis of final cyclohexanol compounds ... 33

4.2.1 *Cis*-4-*tert*-butyl-*trans*-2-methoxy cyclohexanol (**10b**) ... 33

4.2.2 *Cis*-4-*tert*-butyl-*cis*-2-methoxy cyclohexanol (**10c**) ... 33

4.2.3 *Trans*-4-*tert*-butyl-*trans*-2-methoxy cyclohexanol (**11b**) ... 34

4.3 Synthesis of final carbohydrate compounds ... 34

4.3.1 Synthesis of methyl 3,4,6-tri-*O*-methyl-α-D-glucopyranoside (**Glc2**) ... 34

4.3.2 Synthesis of methyl 2,4,6-tri-*O*-methyl-α-D-glucopyranoside (**Glc3**) ... 34

4.3.3 Synthesis of methyl 2,3,6-tri-*O*-methyl-α-D- glucopyranoside (**Glc4**) ... 35

4.3.4 Synthesis of methyl-2,3,4-tri-*O*-methyl-α-D-glucopyranosid (**Glc6**) ... 35

4.3.5 Synthesis of methyl 3,4,6-tri-*O*-methyl-α-D-galactopyranoside (**Gal2**) ... 36

4.3.6 Synthesis of methyl 2,4,6-tri-*O*-methyl-α-D-galactopyranoside (**Gal3**) ... 37

4.3.7 Synthesis of methyl 2,3,6-tri-*O*-methyl-α-D-galactopyranoside (**Gal4**) ... 37

4.3.8 Synthesis of methyl 2,3,4-tri-*O*-methyl-α-D-galactopyranoside (**Gal6**) ... 38

5 Copies of spectra ... 39

5.1 General ... 39

5.2 *Cis*-4-*tert*-butyl-*trans*-2-methoxy cyclohexanol (10b) ... 41

5.3 *Cis*-4-*tert*-butyl-*cis*-2-methoxy cyclohexanol (10c) 42

5.4 *Trans*-4-*tert*-butyl-*trans*-2-methoxy cyclohexanol (11b) 44

5.5 Methyl 3,4,6-tri-*O*-methyl-α-D-glucopyranoside (Glc2), in $CDCl_3$. 45

5.6 Methyl 2,4,6-tri-*O*-methyl-α-D-glucopyranoside (Glc3), in $CDCl_3$. 47

5.7 Methyl 2,3,6-tri-*O*-methyl-α-D-glucopyranoside (Glc4), in $CDCl_3$. 49

5.8 Methyl 2,3,4-tri-*O*-methyl-α-D-glucopyranoside (Glc6), in $CDCl_3$ 51

5.9 Methyl 3,4,6-tri-*O*-methyl-α-D-galactopyranoside (Gal2), in C_6D_6 52

5.10 Methyl 3,4,6-tri-*O*-methyl-α-D-galactopyranoside (Gal2), in $CDCl_3$ 54

5.11 Methyl 2,4,6-tri-*O*-methyl-α-D-galactopyranoside (Gal3), in $CDCl_3$ 55

5.12 Methyl 2,3,6-tri-*O*-methyl-α-D-galactopyranoside (Gal4), in $CDCl_3$ 57

5.13 Methyl 2,3,4-tri-*O*-methyl-α-D-galactopyranoside (Gal6), in $CDCl_3$ 58

5.14 Methyl 2,3,4-tri-*O*-methyl-α-D-galactopyranoside (Gal6), in CCl_4 60

1 HB acidity measurement

1.1 FT-IR method

1.1.1 HB acidity/donating capacity scale pK_{AHY}

To isolate the formation of discrete 1:1 HBD•••NMP complexes, dilute solutions of the hydroxylic hydrogen-bond donors (HBDs) and the reference acceptor, *N*-methyl-2-pyrrolidone (NMP), were prepared in spectroscopic-grade CCl_4. This high-dilution regime was strictly maintained to suppress higher-order 1:*n* aggregates and self-association, ensuring that the observed spectral shifts correspond exclusively to the primary heterodimeric interaction. Under these standardised conditions, the thermodynamic hydrogen-bond acidity of organic HBDs is quantified via the pK_{AHY} scale.[1],[2],[3],[4] This scale is defined as the decadic logarithm of the equilibrium constant (K) for the 1:1 complexation at a controlled temperature of 298 K (eq.1–eq.3):

$$HBD + \mathrm{NMP} \leftrightarrow \mathrm{HBD} \cdots \mathrm{NMP}$$

eq.1

$$\frac{\mathrm{K}}{dm^3 \cdot mol^{-1}} = \frac{C_{(HBD \cdots NMP)}}{C_{HBD} \cdot C_{NMP}} = \frac{C_{HBD}^0 - C_{HBD}}{C_{HBD}(C_{NMP}^0 - C_{HBD}^0 + C_{HBD})}$$

eq.2

$$p\mathrm{K}_{AHY} = -\log_{10} \mathrm{K}_{AHY} = +\log_{10} \mathrm{K}$$

eq.3

$$A = \varepsilon_{OH} l C_{HBD}$$

eq.4

On the molar scale, the variables $C_{(HBD \cdots NMP)}$, C_{HBD} and C_{NMP} in eq.2 are the equilibrium concentrations for the complex, the H-bond donor, and the H-bond acceptor NMP, while C_{HBD}^0 and C_{NMP}^0 are the initial concentrations are determined via precise gravimetric preparation to ensure high analytical accuracy. Once thermodynamic equilibrium is established, the H-bond donor concentration, C_{HBD}, is obtained from the Beer-Lambert law (eq.4). This is achieved by monitoring the sharp absorption band of the -OH stretching vibration, typically located between 3600–3620 cm^{-1}. A critical preliminary step involves the determination of the molar absorption coefficient (ε_{OH}) for each HBD. This calibration is essential for two reasons: a/**Direct Quantification:** It enables the translation of absorbance intensity into absolute equilibrium concentrations. b/**Aggregation Control:** It confirms the absence of solute self-association. By validating that the HBD remains in a purely monomeric state at the experimental concentrations, the formation of undesirable 1:n H-bond complexes is effectively precluded.

The stretching vibration ν_{OH}, $\nu_{OH\cdots NMP}$, the half-width $\Delta\nu_{1/2}$, the molar absorption coefficient ε_{OH}, equilibrium constant K, pK_{AHY} and the standard Gibbs free energy change (ΔG_{AHY})were determined for the **10a**[5], **10b**, **10c**, **10d**[5], **10e**[5], **11a**[5], **11b**, **11c**[5], **Glc2**, **Glc3**, **Glc4**, **Glc6**, **Gal2**, **Gal3**, **Gal4**, and **Gal6** and reported in Table S1 below.

At the concentrations (~1.0 to $8.0.10^{-3}$ M) employed in this study for the compounds **10b**, **10c**, **11b**, **Glc2**, **Glc3**, **Glc4**, **Glc6**, **Gal2**, **Gal3**, **Gal4**, and **Gal6**, no self-association was observed for either cyclohexanol or the carbohydrate derivatives. This was confirmed by the absence of a characteristic broad absorption band at approximately 3440 cm^{-1}, which typically indicates the presence of intermolecular hydrogen bonding (HB) in alcohols. Reported in Table S2 below.

1.1.2 General Experimental Procedure

Carbon tetrachloride (CCl_4) of spectroscopic grade was dried for several days over freshly activated 4 Å molecular sieves prior to use. Similarly, *N*-methyl-2-pyrrolidone (NMP) (99.5% purity) was stored over molecular sieves in total darkness for 48 hours to prevent oxidative degradation. The synthesised cyclohexanol and carbohydrate derivatives were thoroughly dried under high vacuum (1-3 mbar). To maintain anhydrous conditions, all chemical handling, solution preparation, and the filling of IR cells were conducted within the dry atmosphere of a glovebox at ambient temperature.

Infrared (IR) spectra were acquired using a Bruker Vertex 70 Fourier-transform spectrometer at a resolution of 1 cm^{-1}. For the hydrogen-bond complexation studies, samples were measured in an Infrasil quartz cell with a path length of S=1 cm. To ensure thermal stability and reproducibility, the cell was thermostated at 25.0±0.2 °C using a Peltier effect regulation system. All measurements were performed using CCl_4 as the solvent.

1.1.3 Sample Preparation

1.1.3.1 Epsilon value measurement (check the possibility of self-association)

To a clean 20 mL glass vial, approximately 100–180 mg of the sample compound was added. The vial was then filled with roughly 10 mL of CCl_4, and the mixture was agitated to ensure the sample was completely dissolved. Gravimetric data were recorded at each stage: the mass of the empty vial, the vial with the sample, and the vial containing both the sample and solvent. A small aliquot of this stock solution was transferred into a 10 mL volumetric flask, which was then diluted to the calibration mark with CCl_4. The weights of the empty volumetric flask, the flask with the added aliquot, and the final filled flask were recorded, respectively, to determine the precise concentration. Subsequently, the mixture was transferred into an Infrasil quartz cell, and the IR spectrum was recorded. Prior to the measurement of the sample, the background IR spectrum of pure CCl_4 was recorded as a reference. This entire procedure was repeated four

additional times to produce a concentration range of 2.00×10^{-3} M to 7.00×10^{-3} M. To ensure statistical reliability and minimise experimental error, the complete process was performed in duplicate, resulting in a total of 10 data sets.

1.1.3.2 Concentration measurement upon 1:1 complexation with NMP:

1.1.3.2.1 Preparation of NMP stock and reference

Approximately 700–900 mg of *N*-methyl-2-pyrrolidone (NMP) was added to a clean 20 mL glass vial. Roughly 10 mL of CCl_4 was added to the vial, and the mixture was agitated to ensure the NMP was completely dissolved. Gravimetric data were recorded at each stage: the empty vial, the vial containing the NMP, and the vial containing the final NMP and CCl_4 solution. A small aliquot of this mixture was transferred into a 10 mL volumetric flask and diluted to the calibration mark with CCl_4. The mass of the empty volumetric flask, the flask with the added mixture, and the final filled flask were recorded, respectively. This solution was then transferred into an Infrasil quartz cell, and the IR spectrum was recorded as a reference.

1.1.3.2.2 Sample–NMP complex preparation

A sample solution was prepared beforehand using a similar gravimetric method. To prepare the complex mixtures, a designated amount of the NMP stock was combined with the sample mixture in a 10 mL volumetric flask. The final volume was adjusted with CCl_4 such that the sample concentration remained constant at approximately 4.00×10^{-3} M. The mass of the flask was recorded at every step of the addition process. Once prepared, the mixture was transferred into an Infrasil quartz cell for IR spectral recording.

1.1.3.2.3 Concentration range and statistical reliability

This procedure was repeated to generate a series of solutions where the NMP concentration ranged from 6.00×10^{-3} M to 10.00×10^{-3} M, while the sample concentration was held constant at 4.00×10^{-3} M. To minimise experimental error and ensure reproducibility, the entire procedure was repeated to obtain a total of four data sets for the sample-NMP complex.

1.2 IR experimental data (Table S1)

Table S1. Spectroscopic and thermodynamic experimental data of **10a**[5], **10b**, **10c**, **10d**[5], **10e**[5], **11a**[5], **11b**, **11c**[5], **Glc2**, **Glc3**, **Glc4**, **Glc6**, **Gal2**, **Gal3**, **Gal4**, and **Gal6**

Samples	ν_{OH} ^{a}	$\nu_{OH...NMP}$ ^{a}	$\Delta\nu_{1/2}$ ^{a}	ε_{OH} ^{b}	*K*	pK_{AHY}	ΔG_{AHY} ^{c}
**10a**	3627	3454	15	79	5.10	0.71	-9.80
**10b**	3629	3437	17	86	9.06	0.96	-5.48
**10c**	3586	3438	40	62	0.50	-0.30	1.71
**10d**	3631	3417	17	99	19.94	1.30	-13.20
**10e**	3613	3419	16	155	2.70	0.43	-8.20
**11a**	3623	3445	19	67	5.06	0.70	-9.80
**11b**	3595		25	84	0.80	-0.09	0.55
**11c**	3616	3419	16	130	3.63	0.56	-9.00
**Glc2**	3579	3411	20	101	1.55	0.19	-1.08
	3600	---	---	---			
**Glc3**	3601	3415	30	78	2.24	0.35	-1.20
**Glc4**	3600	3410	33	60	3.08	0.49	-2.80
	3534	---	50	---			
**Glc6**	3609	3438	23	75	1.91	0.28	-1.60
	3640	---	---	---			
	3536	---	---	---			
**Gal2**	3579	3428	36	76	1.09	0.04	-0.22
	3600	---	54				
**Gal3**	3597	3424	27	80	1.66	0.22	-1.25
	3571	---	---				
**Gal4**	3579	3450	46	59	0.62	-0.21	1.20
**Gal6**	3644	---	56				
	3614	3442	49	47	6.03	0.78	-4.45
	3524	---	75				

[a] cm^{-1}. [b] $dm^{3}\ mol^{-1}\ cm^{-1}$. [c] $\Delta G_{AHY}/kJmol^{-1} = -RTln(10)pK_{AHY} = -5.708pK_{AHY}$ at 298 K. Where ν_{OH}: ν_{OH} *absorption band of the OH;* $\nu_{OH...NMP}$: the $\nu_{OH...NMP}$ intermolecular complex band; $\Delta\nu_{1/2}$: half-width at half-maximum; ε_{OH}: absorption coefficient.

1.3 IR Spectra

1.3.1 Comparison IR spectra

In the spectroscopic analysis of cyclohexanol and sugar derivatives, the characteristic infrared absorption bands of the hydroxyl group provide critical insight into the molecule's conformational profile and the nature of Intramolecular Hydrogen Bonding (IMHB). General wavenumber ranges have been proposed to distinguish between these interactions: the absorption band observed around 3630 cm^{-1} is characteristic of free secondary alcohol.[6],[7],[8],[9] Followed by the absorption bands observed around 3600 cm^{-1}, which typically correspond to a *trans*-diequatorial-substituted donor-acceptor pair, where the geometric orientation results in a relatively weak interaction. In contrast, bands appearing at lower frequencies, often ranging around 3585 cm^{-1}, generally indicate stronger IMHB associated with *cis*-1,2-substituted or axial-equatorial arrangements, as these configurations allow for closer proximity between the hydroxyl proton and the acceptor atom.[6],[10],[11],[12],[13] For a 6-membered IMHB, a (broad) band around 3530 cm^{-1} is observed.[11, 14]

1.3.2 Model cyclohexanol derivatives

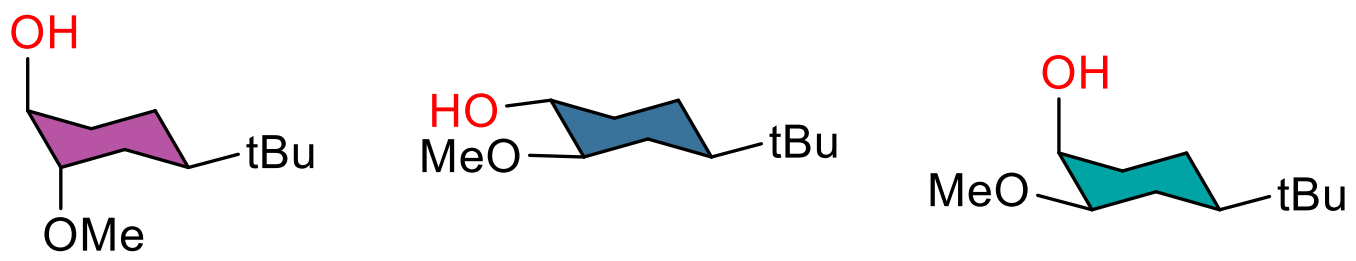

10b **11b** **10C**

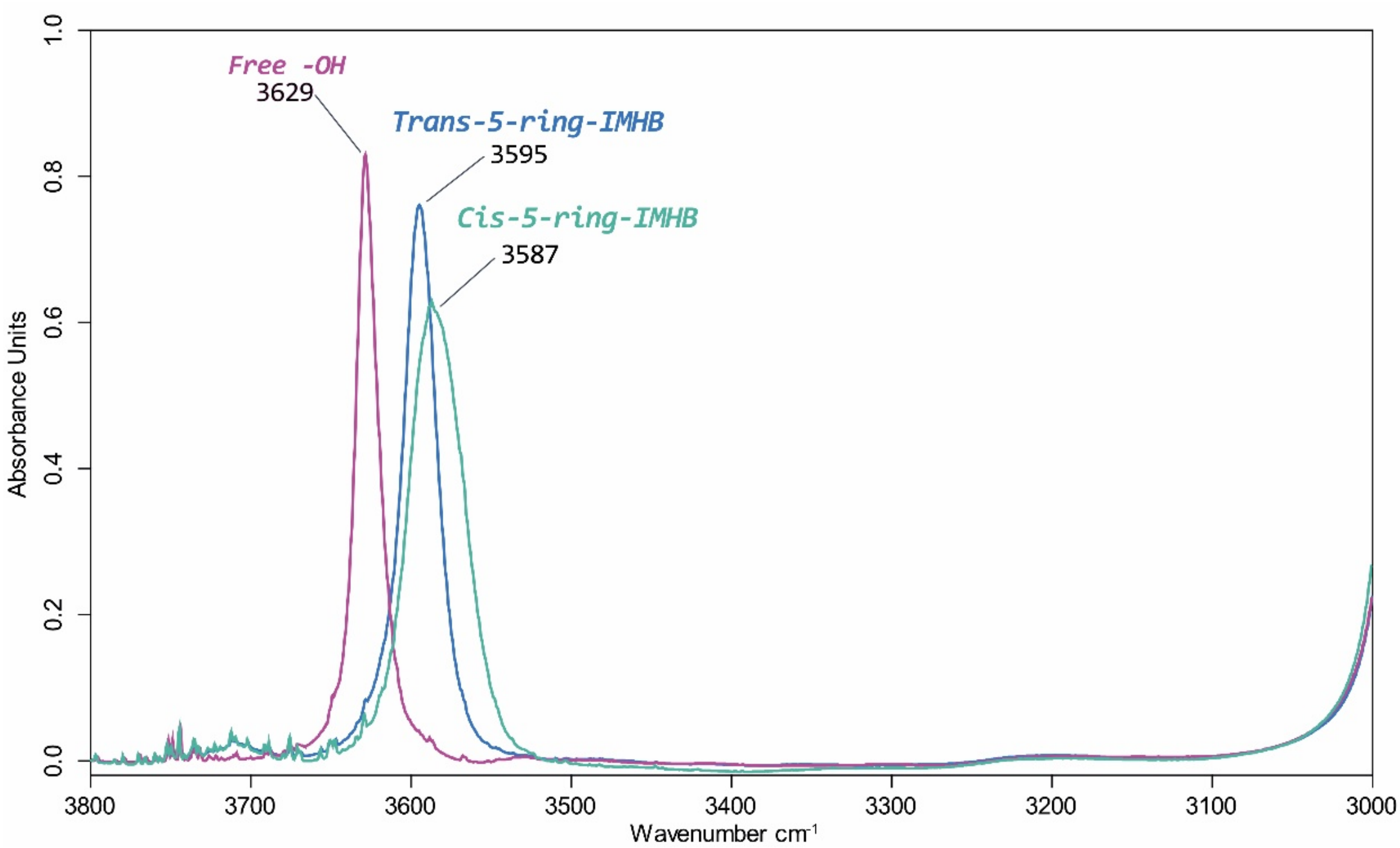

The observed data follow the general wave number range mentioned above.

1.3.3 Glucose derivatives

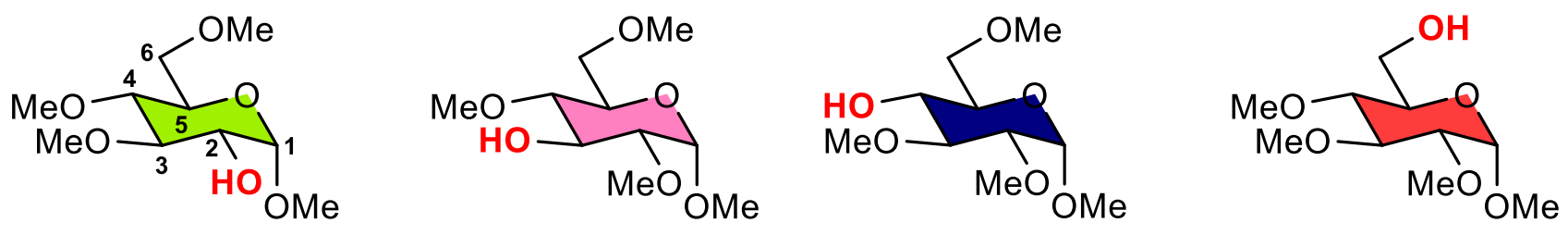

Glc2 **Glc3** **Glc4** **Glc6**

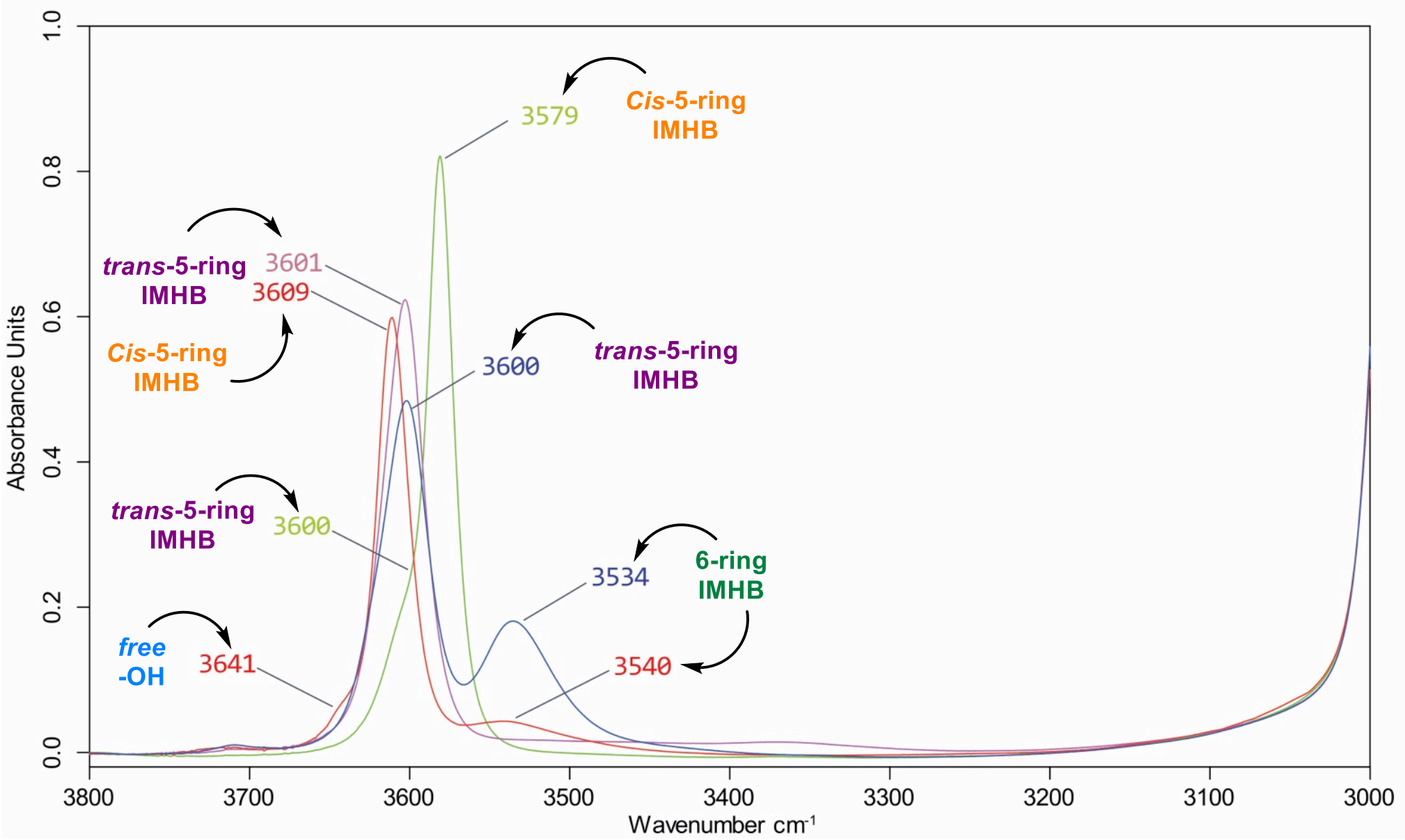

The observed data follow the general wave number range mentioned above.

1.3.4 Galactose derivatives

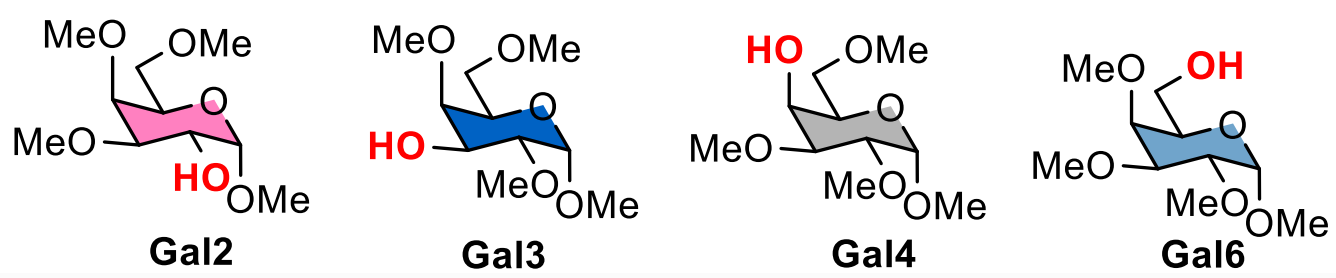

Gal2 **Gal3** **Gal4** **Gal6**

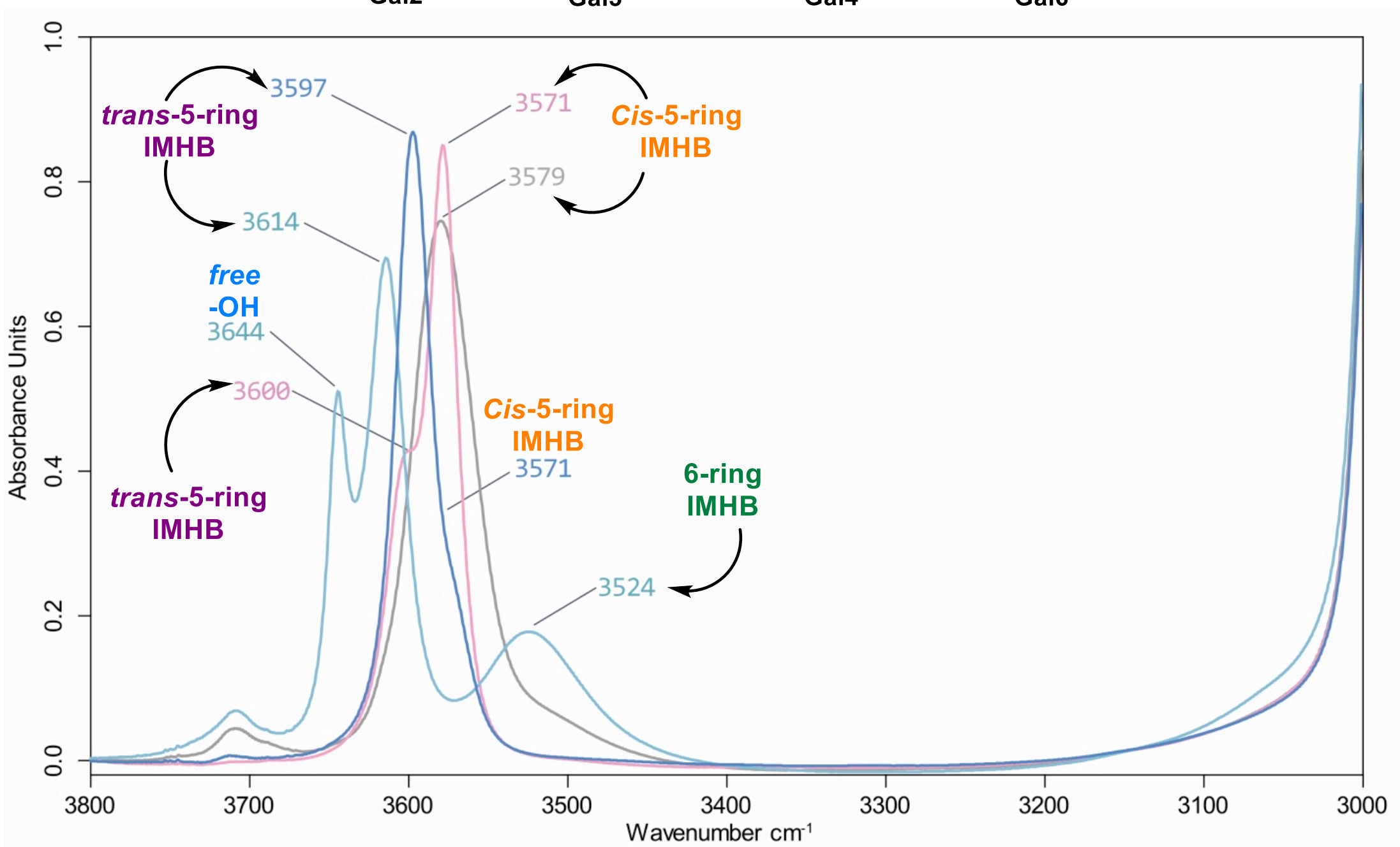

The observed data follow the general wave number range mentioned above.

1.3.5 Individual IR spectra (Table S2)

The effect of a slight concentration variation has been investigated for the 11 compounds from 1.0 to 8.0.10^{-3} M, clearly showing that no low-frequency band (intermolecular HB because of self-association) appears for **10b**, **10c**, **11b**, **Glc2**, **Glc3**, **Glc4**, **Glc6**, **Gal2**, **Gal3**, **Gal4**, and **Gal6,** even at the highest concentration value.

Table S2: Evolution, upon concentration variations, of the infrared spectra of compounds **10b**, **10c**, **11b**, **Glc2**, **Glc3**, **Glc4**, **Glc6**, **Gal2**, **Gal3**, **Gal4**, and **Gal6** in CCl_4 solutions (Side A). Evolution of the infrared spectra of compounds **10b**, **10c**, **11b**, **Glc2**, **Glc3**, **Glc4**, **Glc6**, **Gal2**, **Gal3**, **Gal4**, and **Gal6** in CCl_4 solutions with increasing amounts of the NMP H-bond acceptor. The additional absorption band is around 3400 cm^{-1} (Side B).

(A) Free -OH and IMHB in CCl4	***(B) Complex with NMP in CCl4***
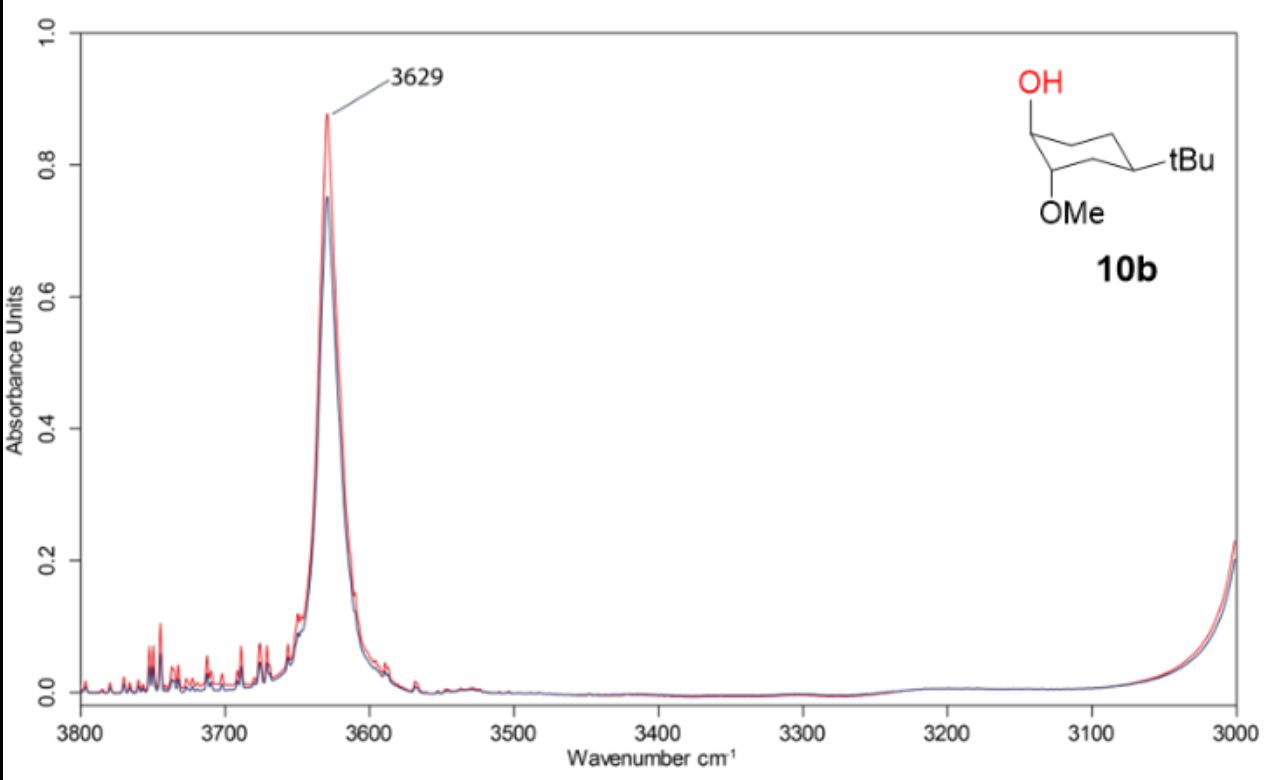	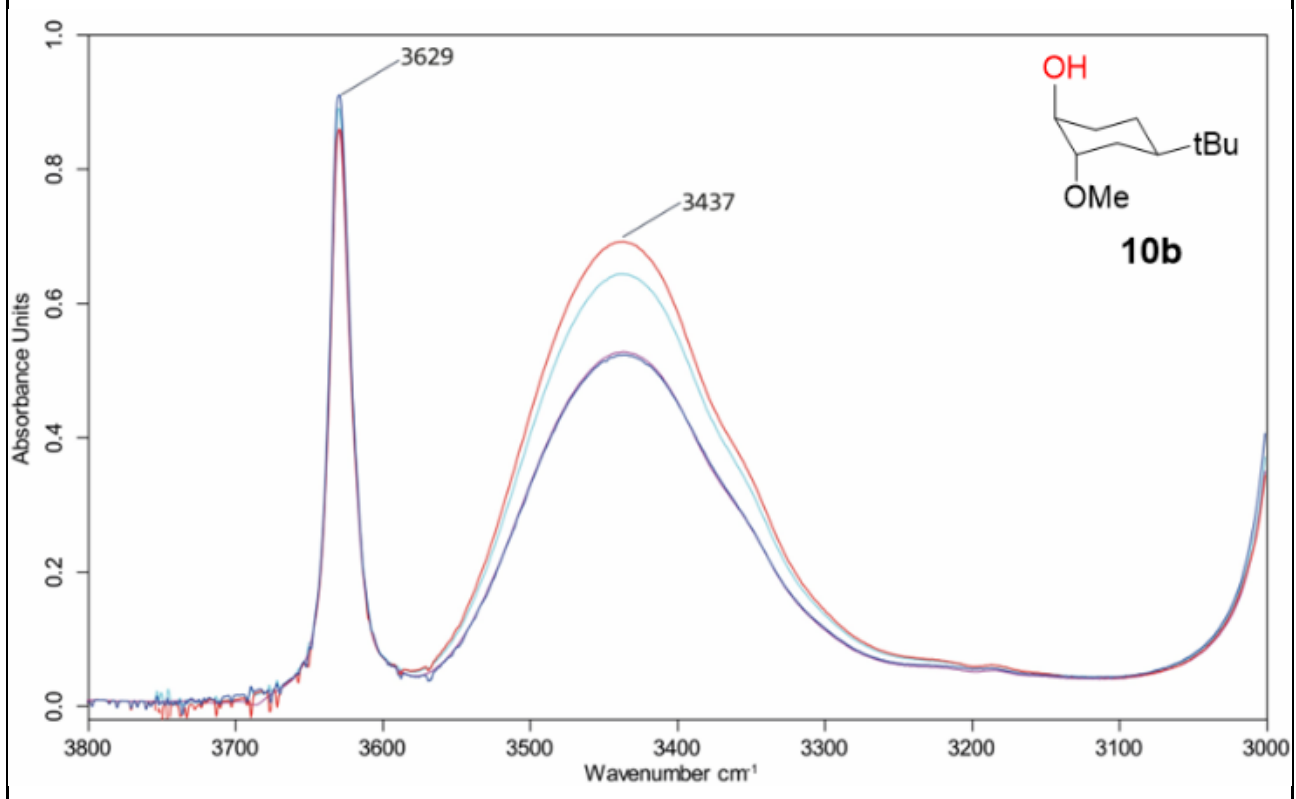
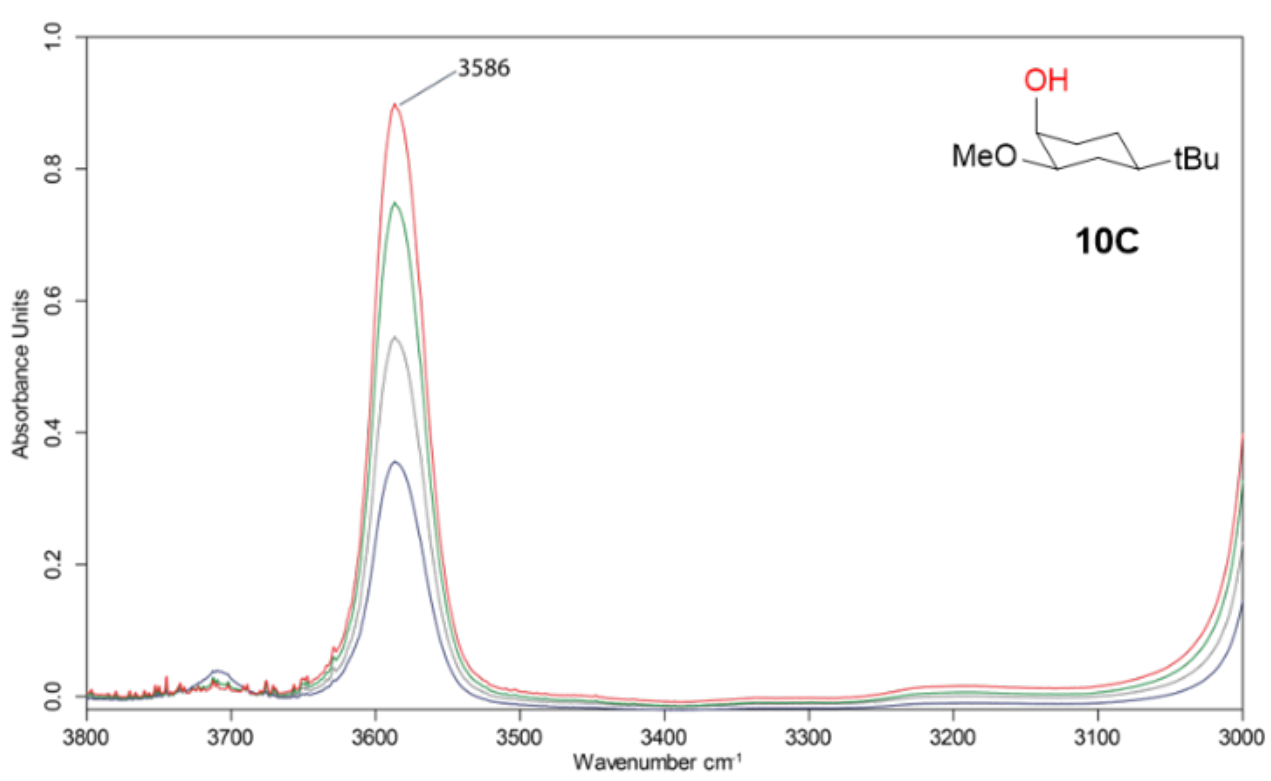	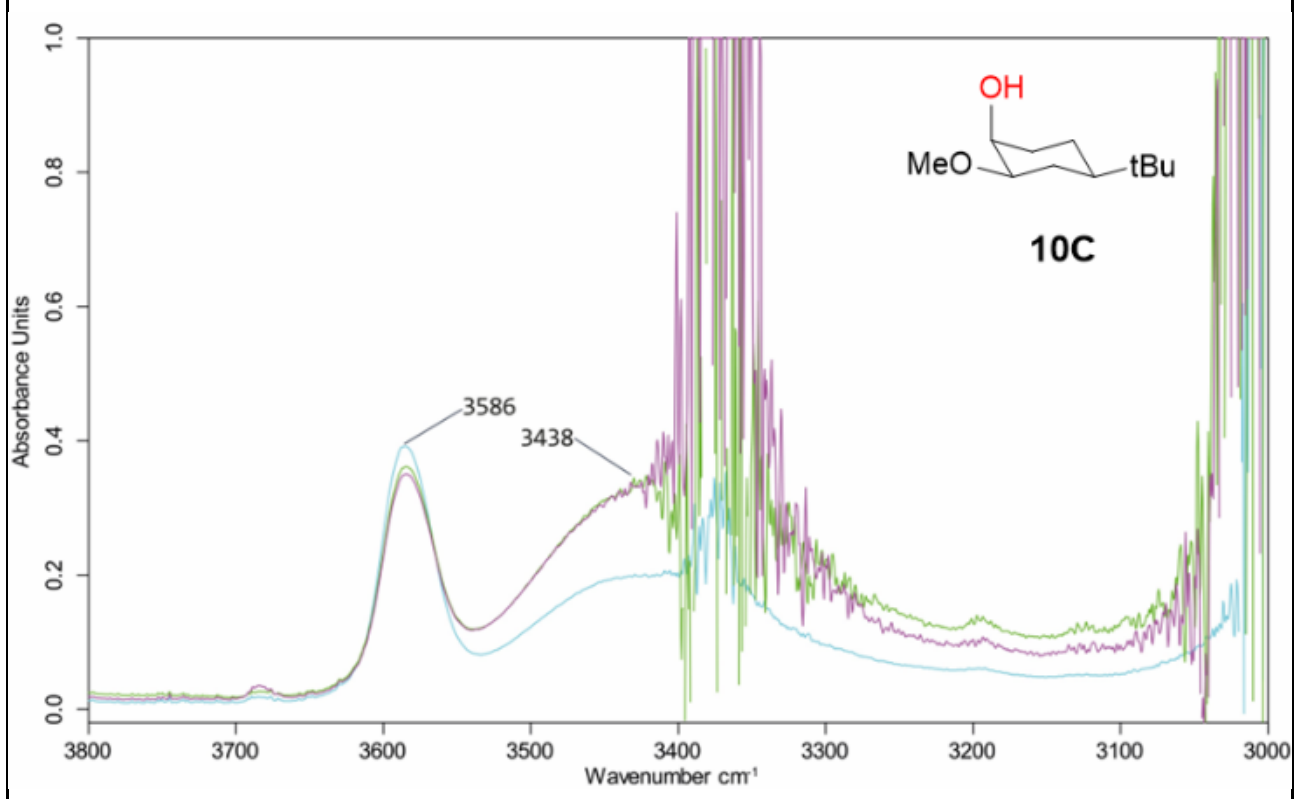

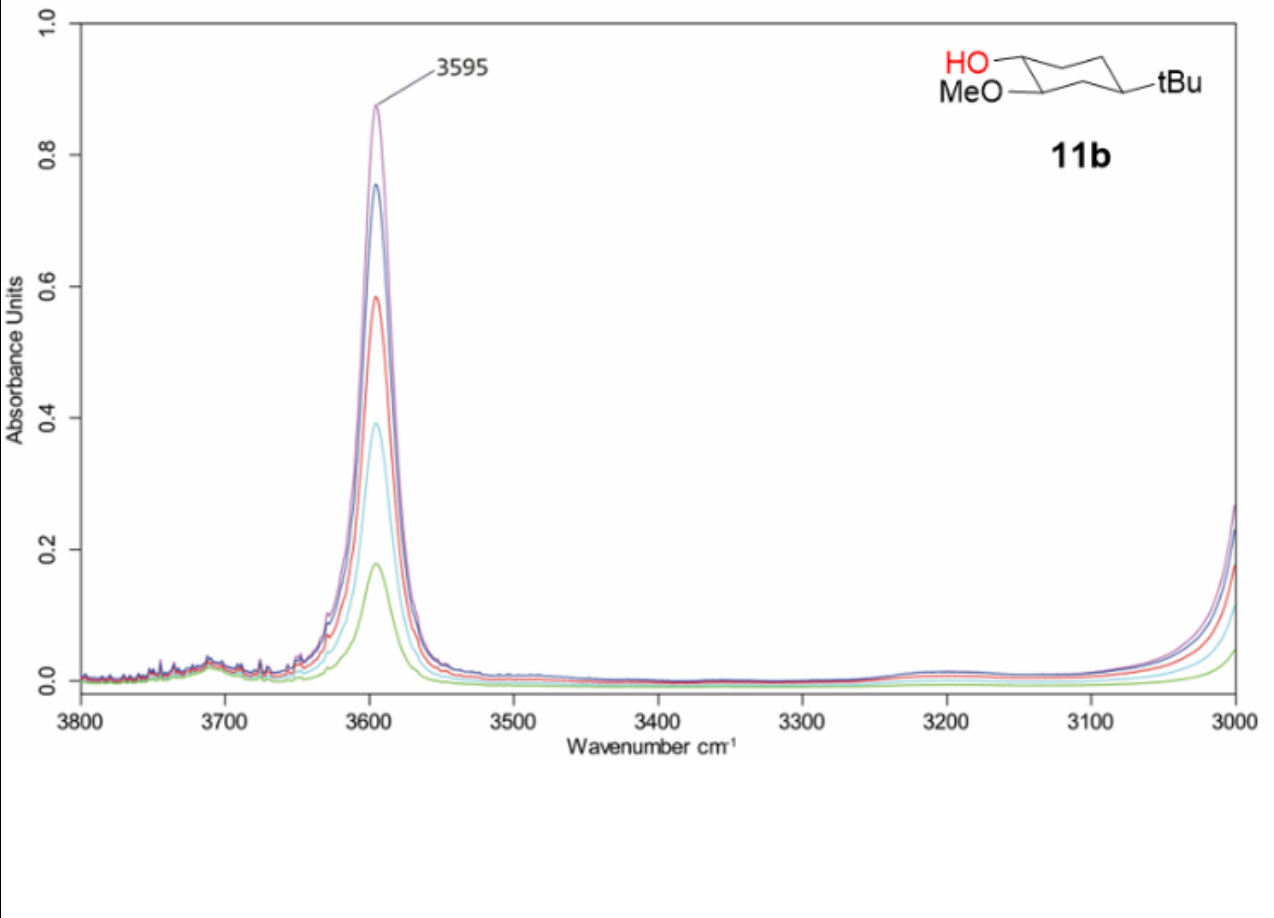
3595
HO
MeO
tBu
11b
Absorbance Units
Wavenumber cm⁻¹

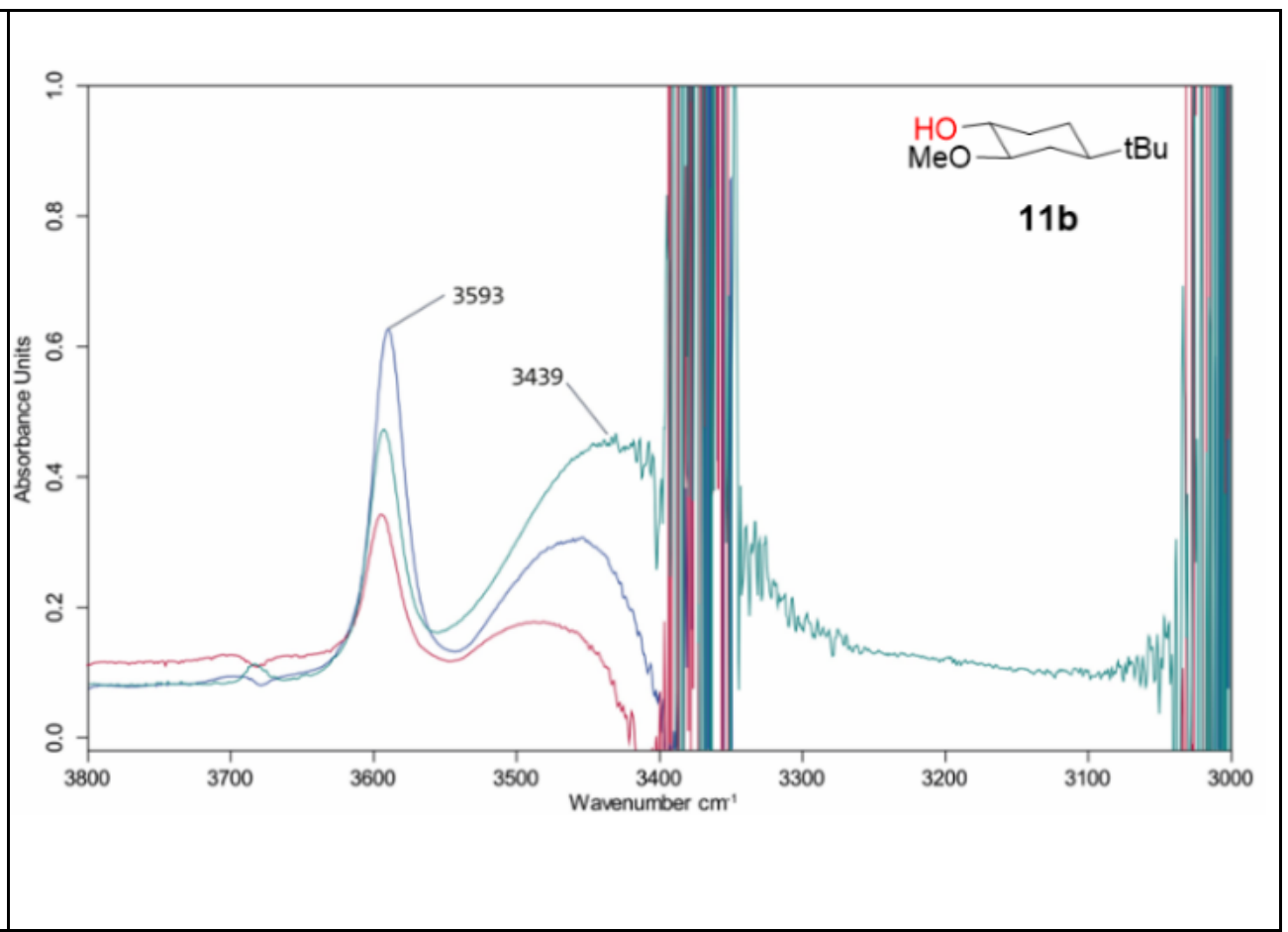
3593
3439
HO
MeO
tBu
11b
Absorbance Units
Wavenumber cm⁻¹

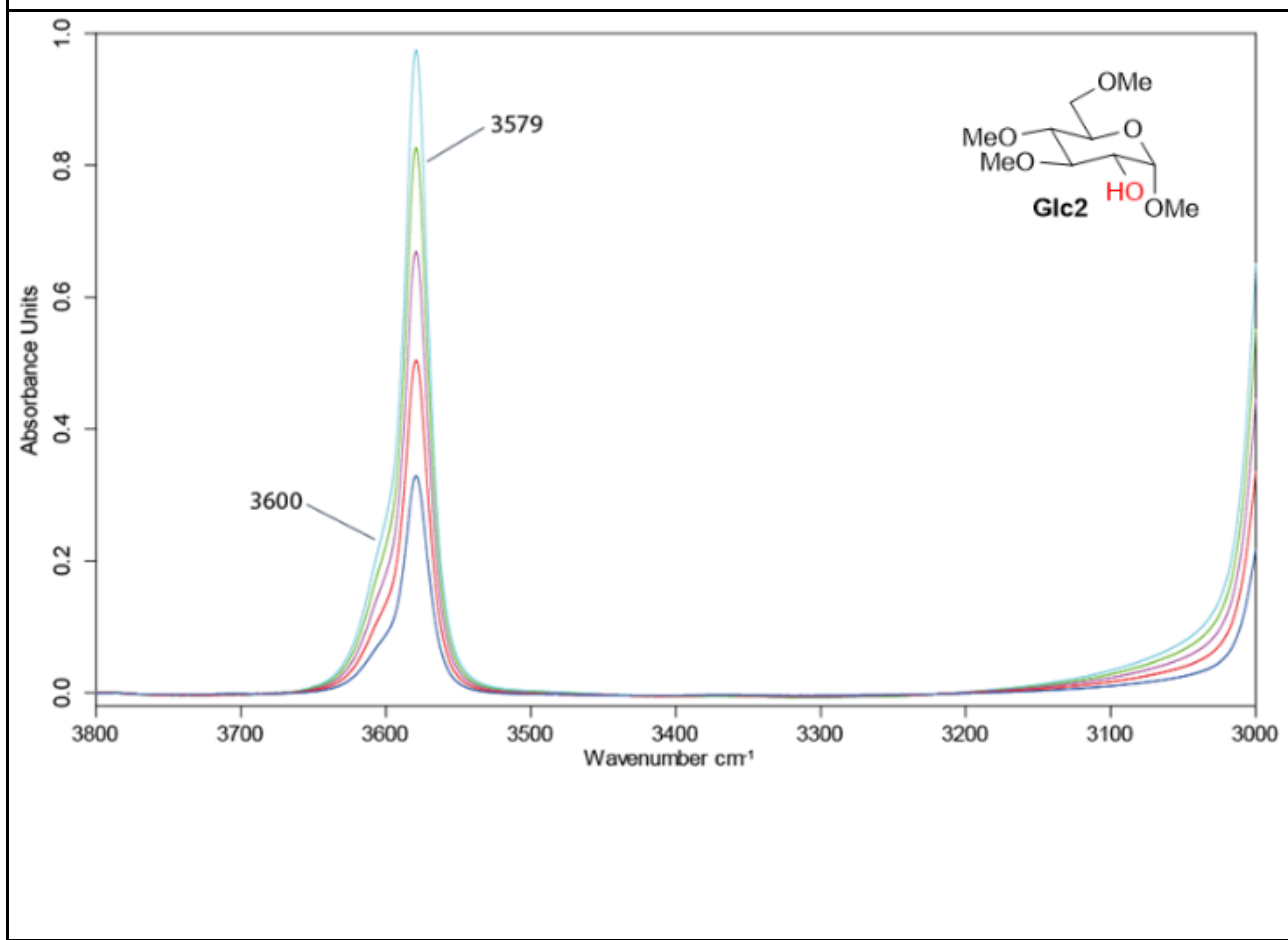
3579
3600
OMe
MeO
MeO
O
HO
OMe
Glc2
Absorbance Units
Wavenumber cm⁻¹

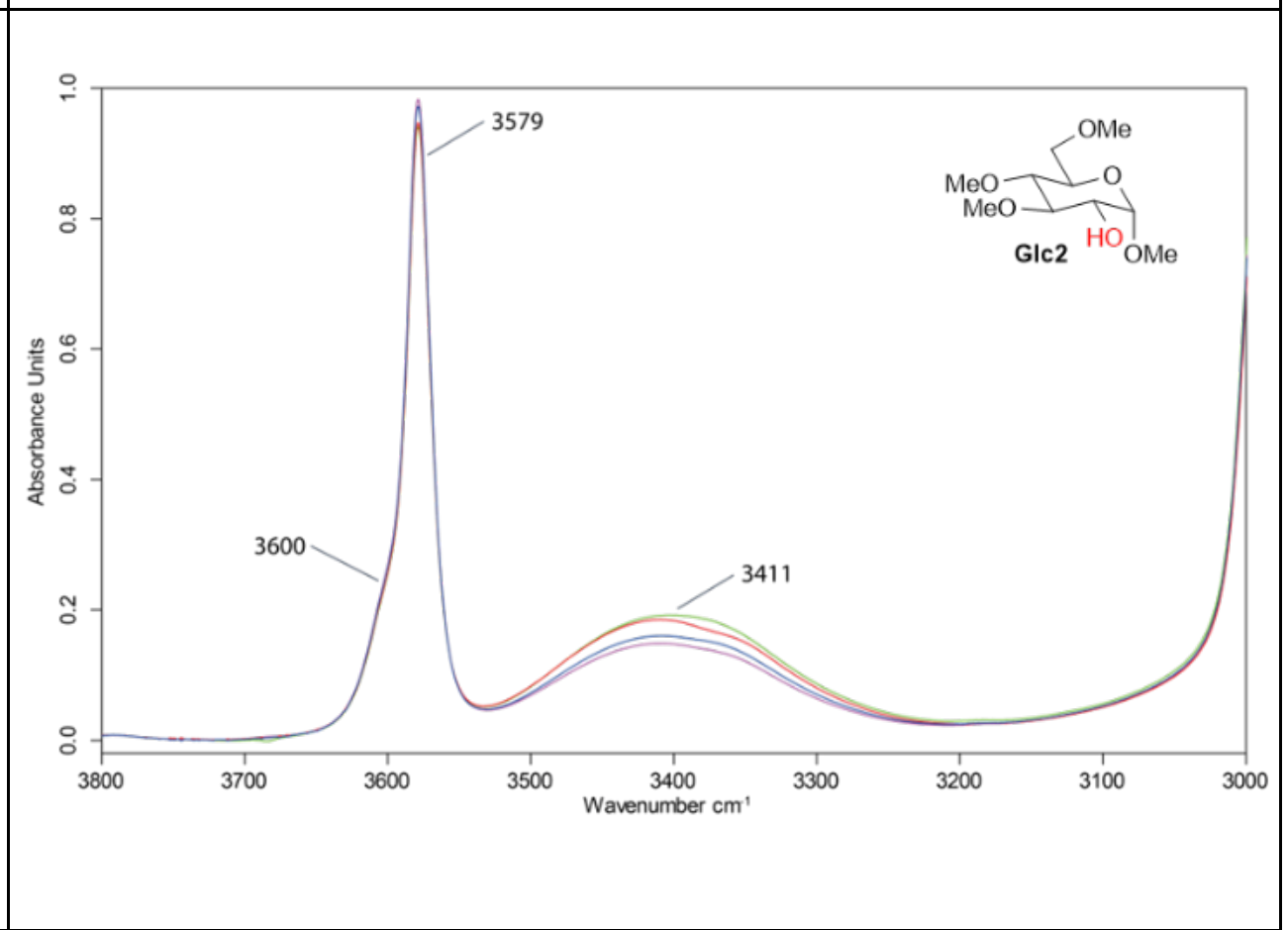
3579
3600
3411
OMe
MeO
MeO
O
HO
OMe
Glc2
Absorbance Units
Wavenumber cm⁻¹

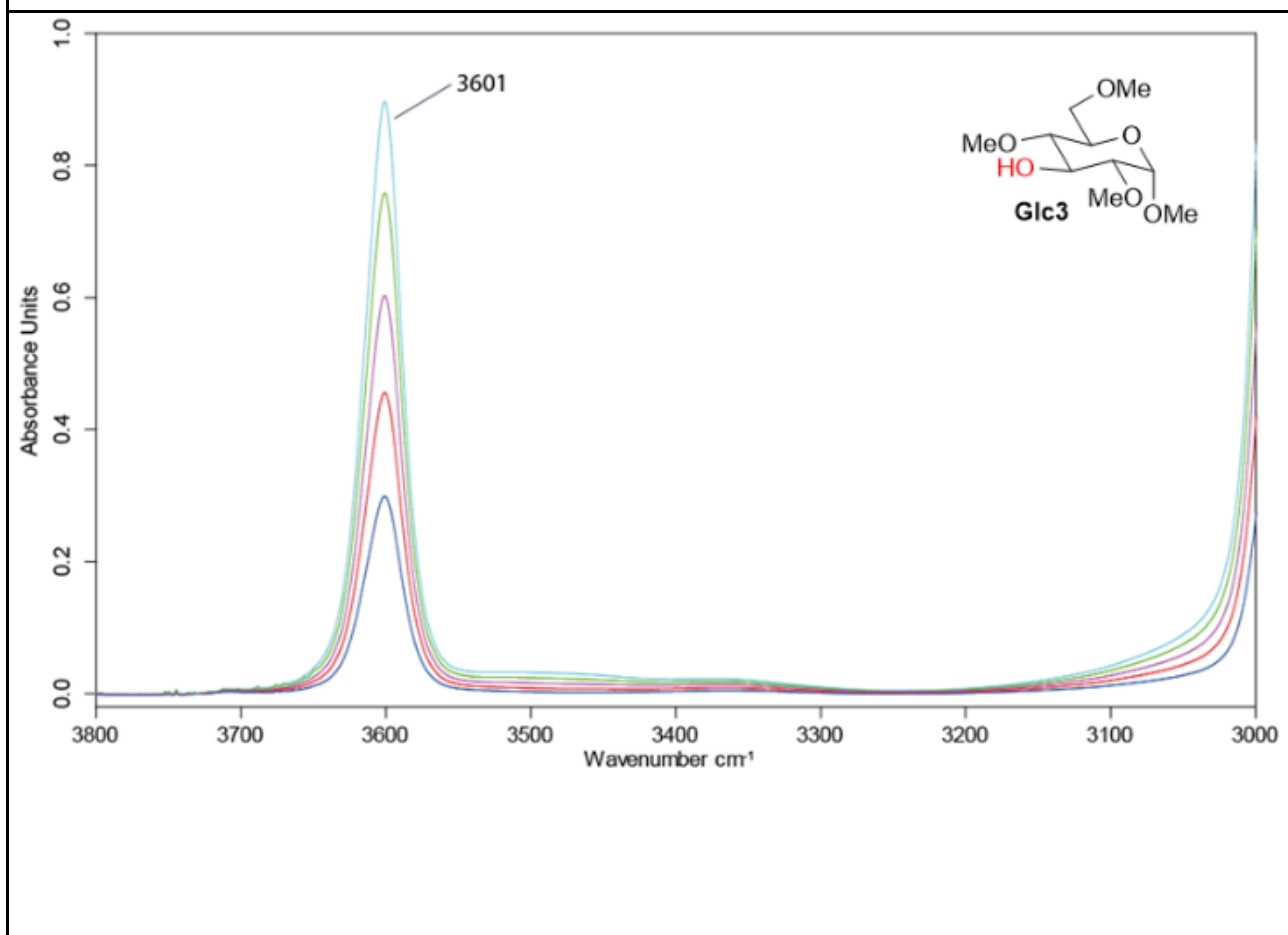
3601
OMe
MeO
HO
O
MeO
OMe
Glc3
Absorbance Units
Wavenumber cm⁻¹

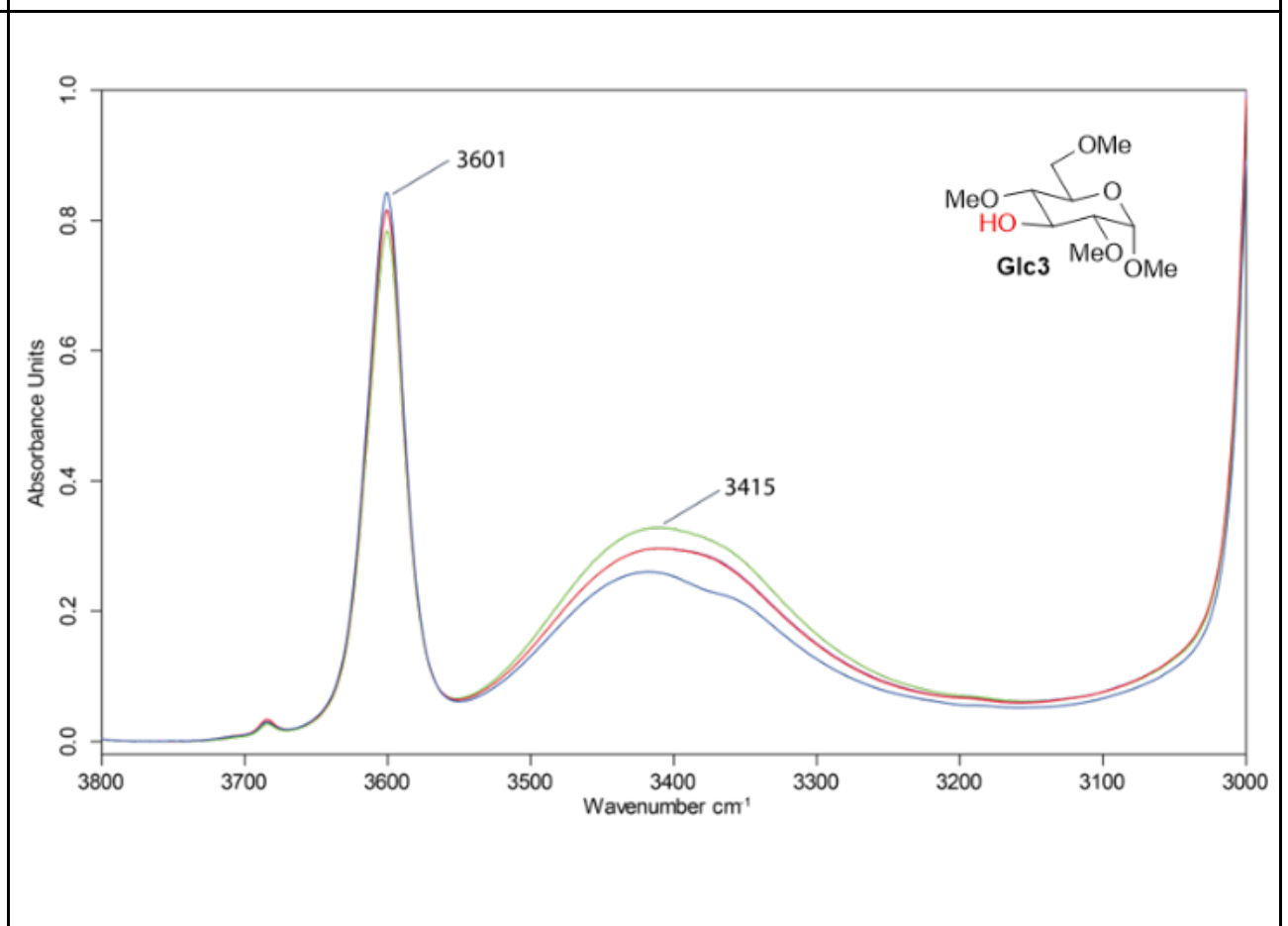
3601
3415
OMe
MeO
HO
O
MeO
OMe
Glc3
Absorbance Units
Wavenumber cm⁻¹

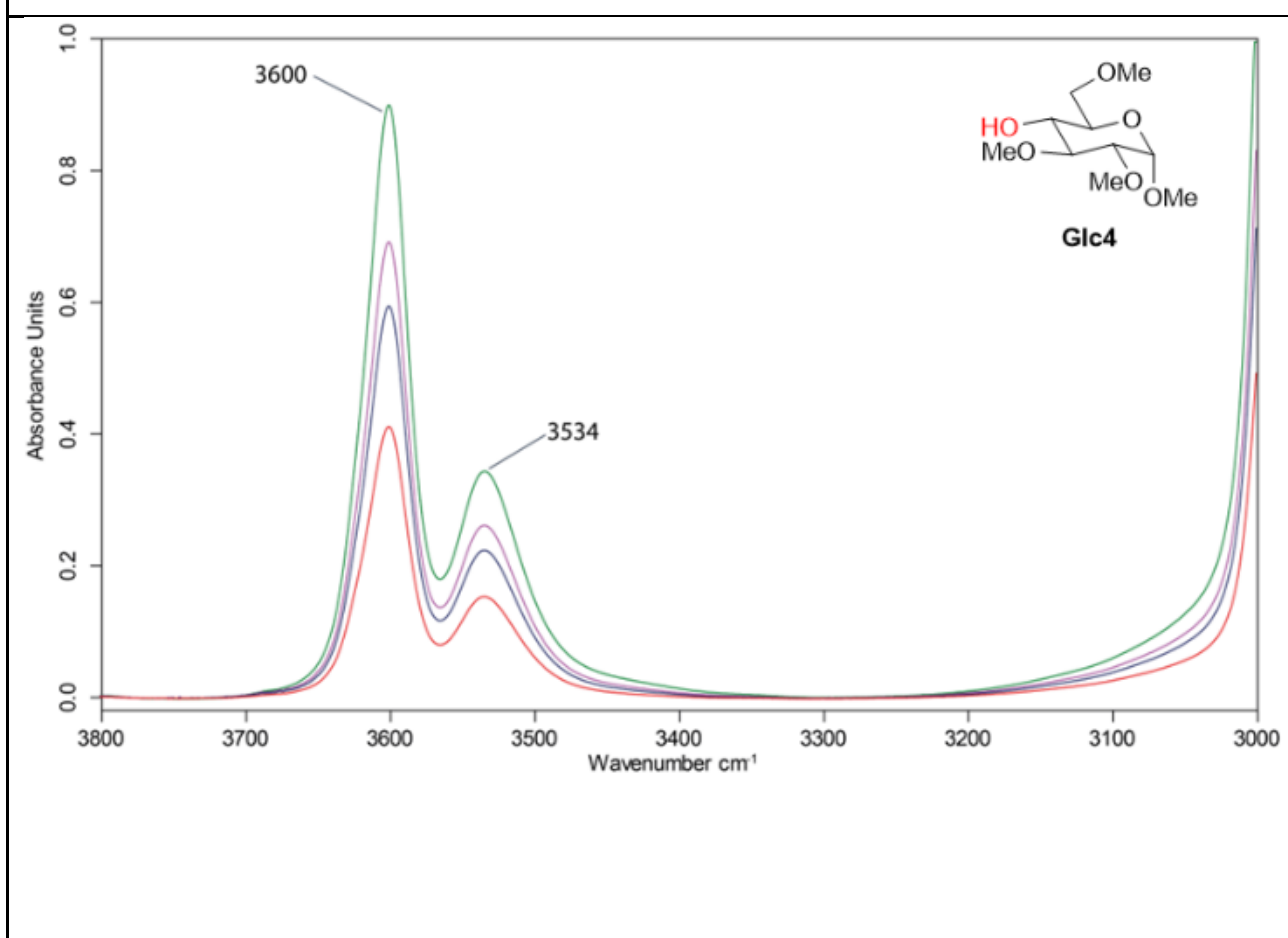
3600
3534
OMe
HO
MeO
O
MeO
OMe
Glc4
Absorbance Units
Wavenumber cm⁻¹

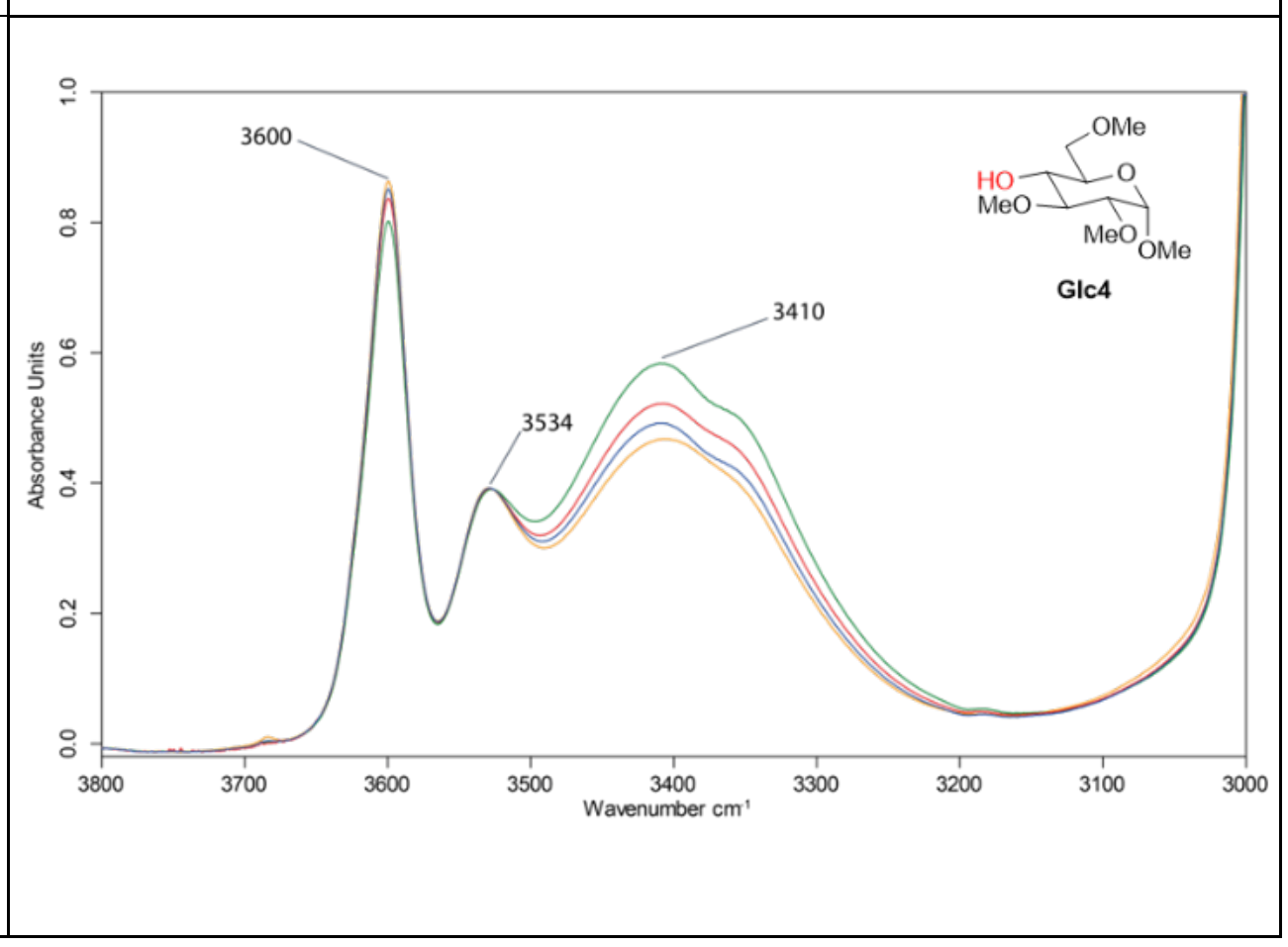
3600
3534
3410
OMe
HO
MeO
O
MeO
OMe
Glc4
Absorbance Units
Wavenumber cm⁻¹

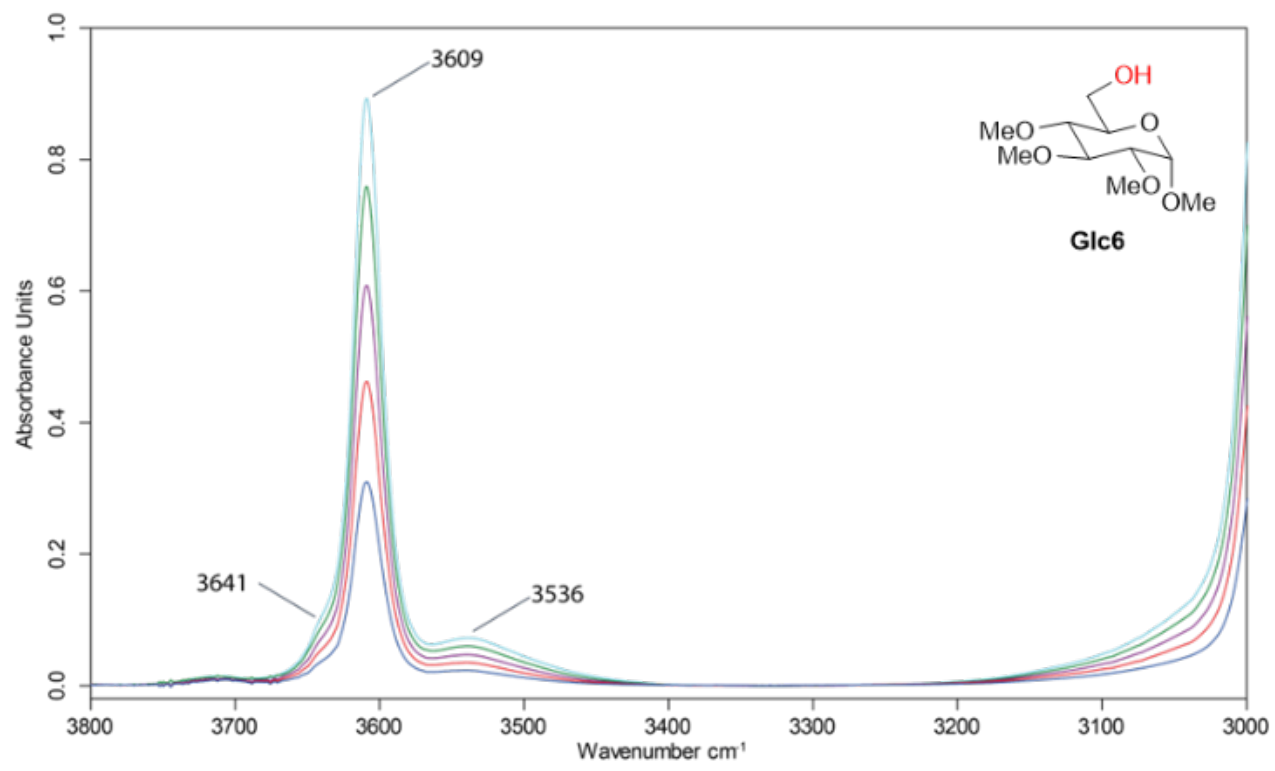
3609
3641
3536
OH
MeO
MeO
O
MeO
OMe
Glc6
Absorbance Units
Wavenumber cm⁻¹

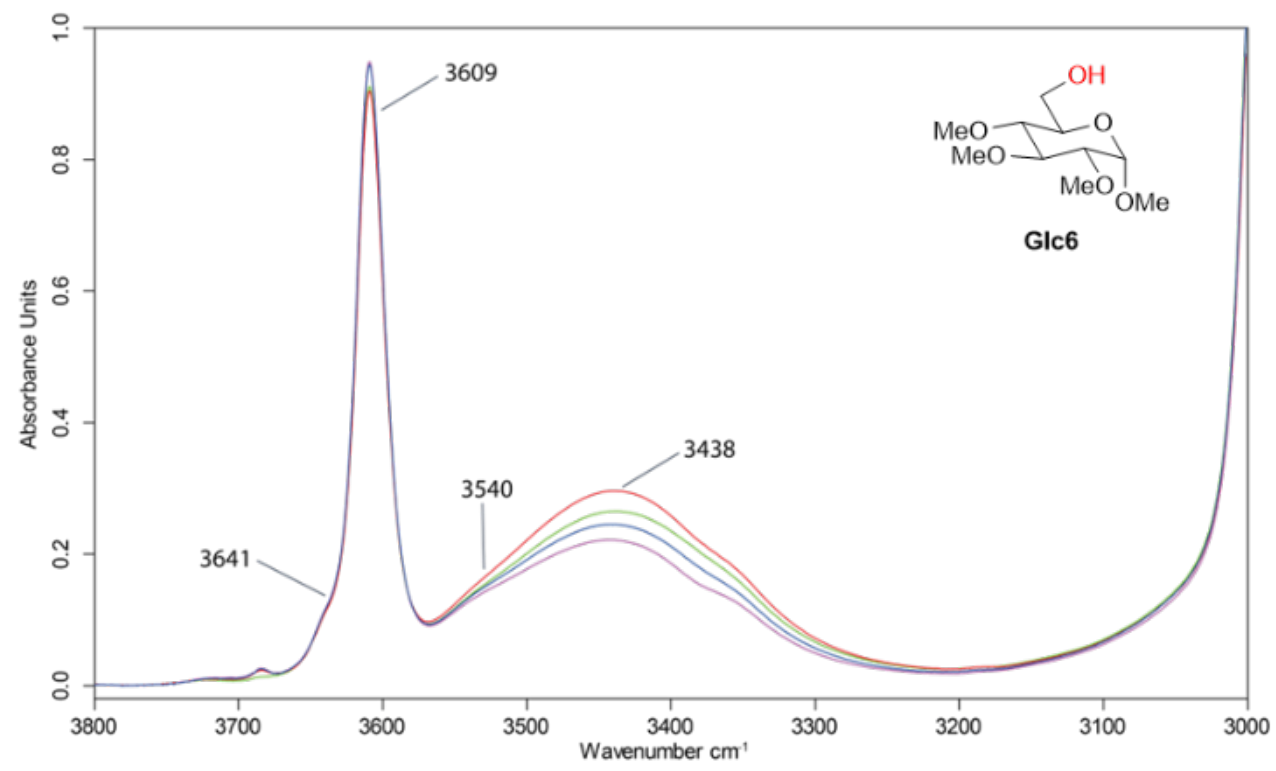
3609
3641
3540
3438
OH
MeO
MeO
O
MeO
OMe
Glc6
Absorbance Units
Wavenumber cm⁻¹

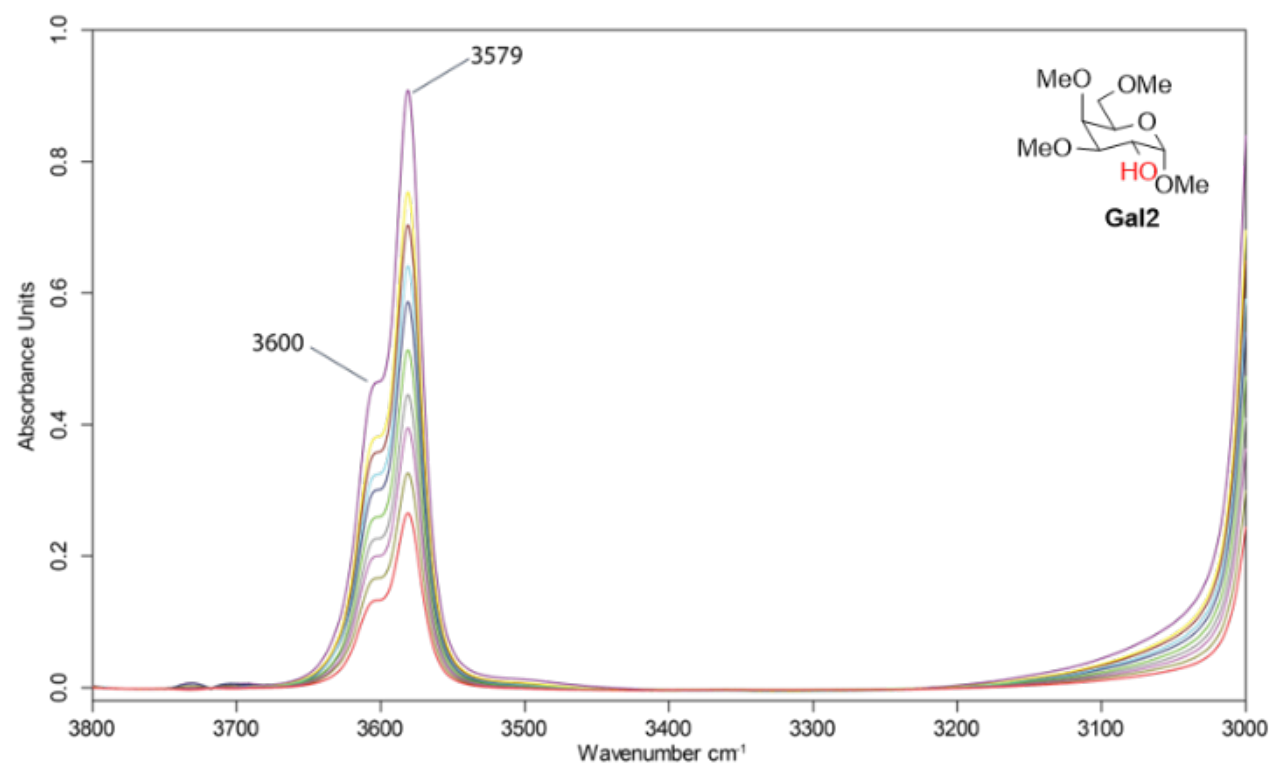
3579
3600
MeO
OMe
O
MeO
HO
OMe
Gal2
Absorbance Units
Wavenumber cm⁻¹

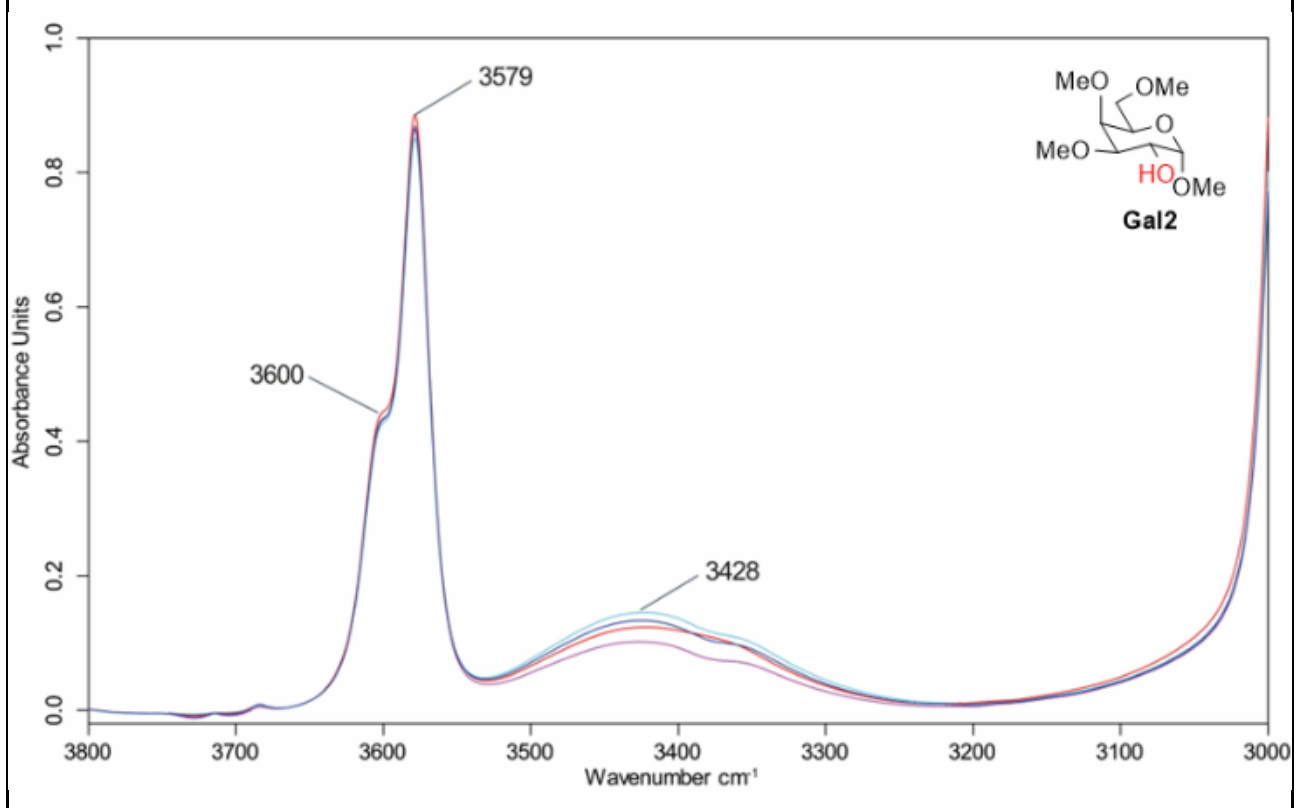
3579
3600
3428
MeO
OMe
O
MeO
HO
OMe
Gal2
Absorbance Units
Wavenumber cm⁻¹

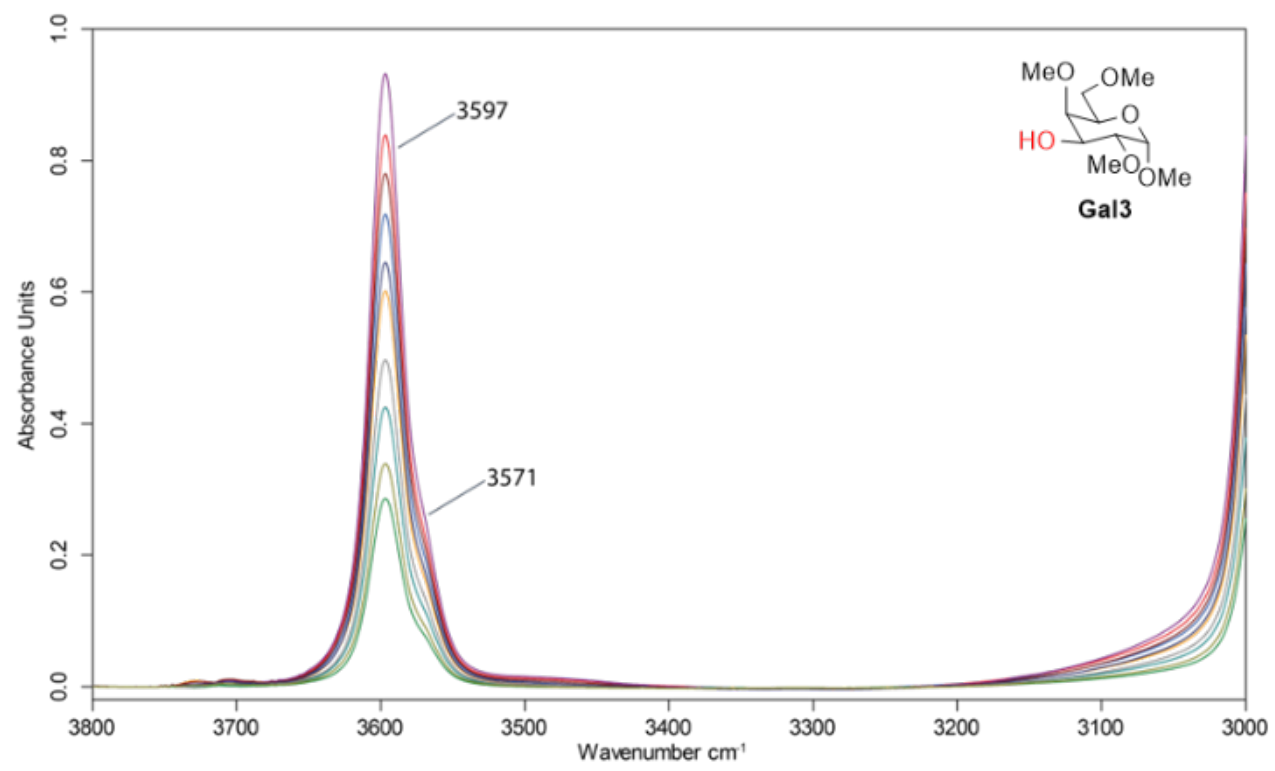
3597
3571
MeO
OMe
O
HO
MeO
OMe
Gal3
Absorbance Units
Wavenumber cm⁻¹

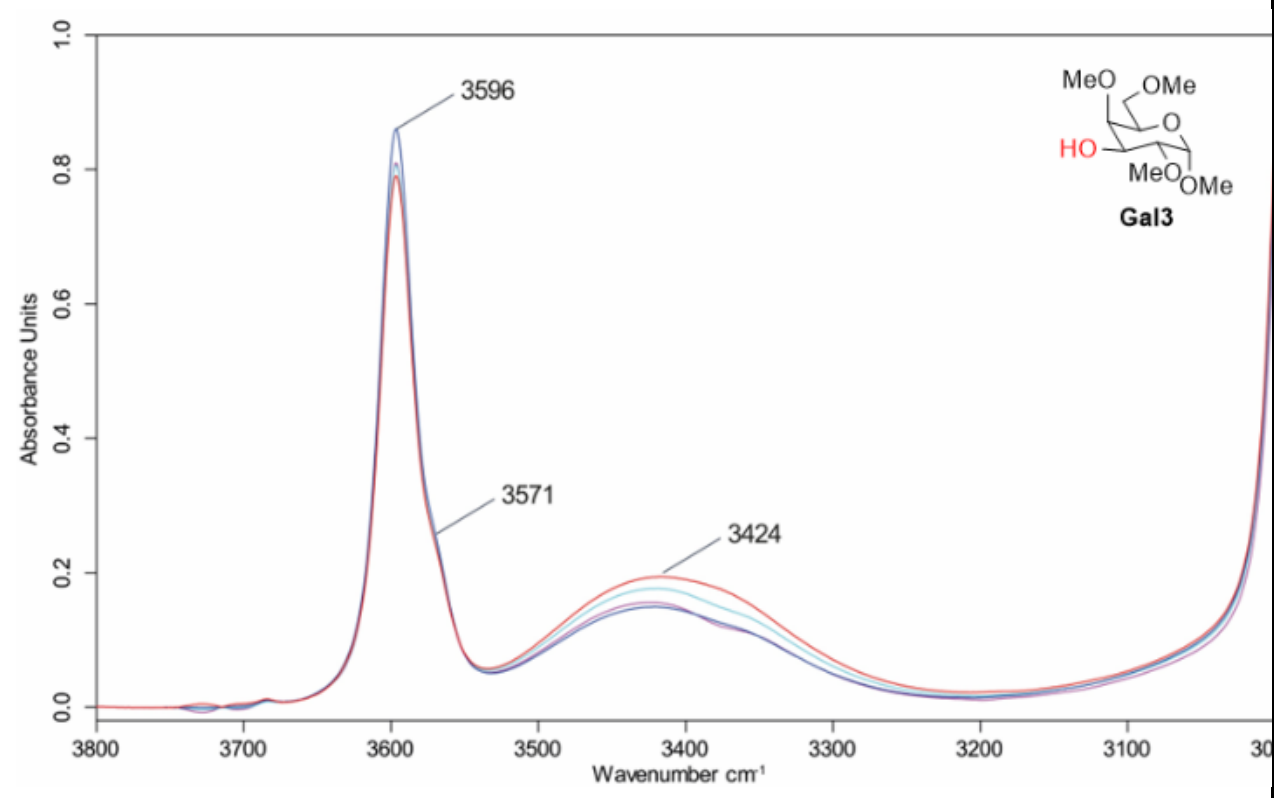
3596
3571
3424
MeO
OMe
O
HO
MeO
OMe
Gal3
Absorbance Units
Wavenumber cm⁻¹

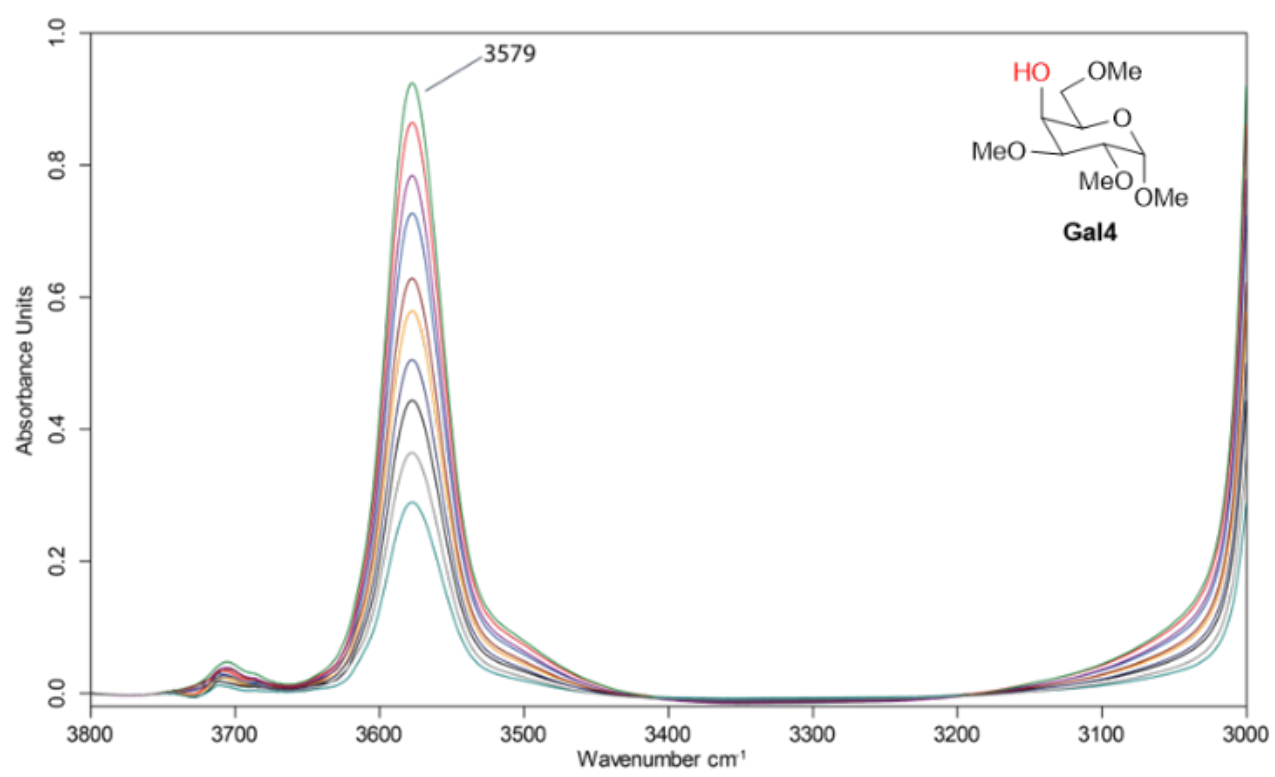
3579
HO
OMe
O
MeO
MeO
OMe
Gal4
Absorbance Units
Wavenumber cm⁻¹

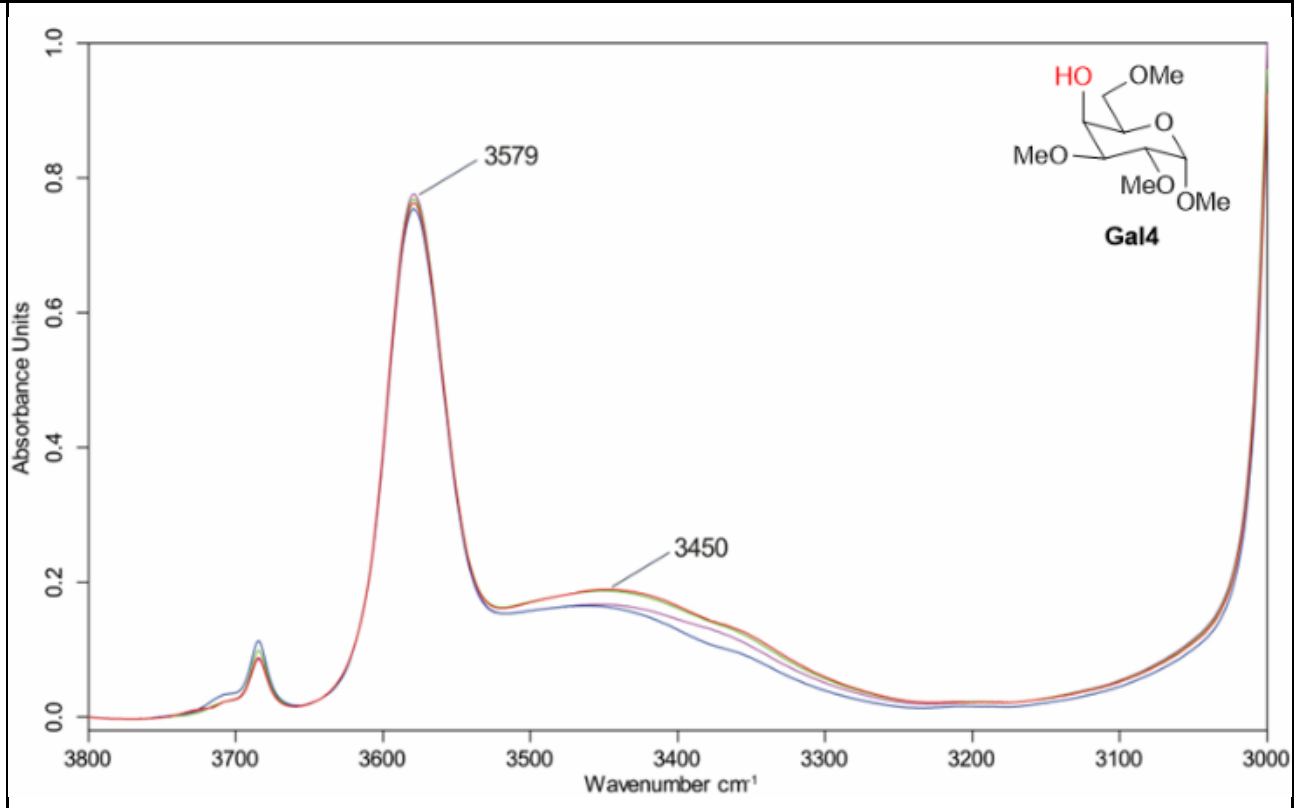
3579
3450
HO
OMe
O
MeO
MeO
OMe
Gal4
Absorbance Units
Wavenumber cm⁻¹

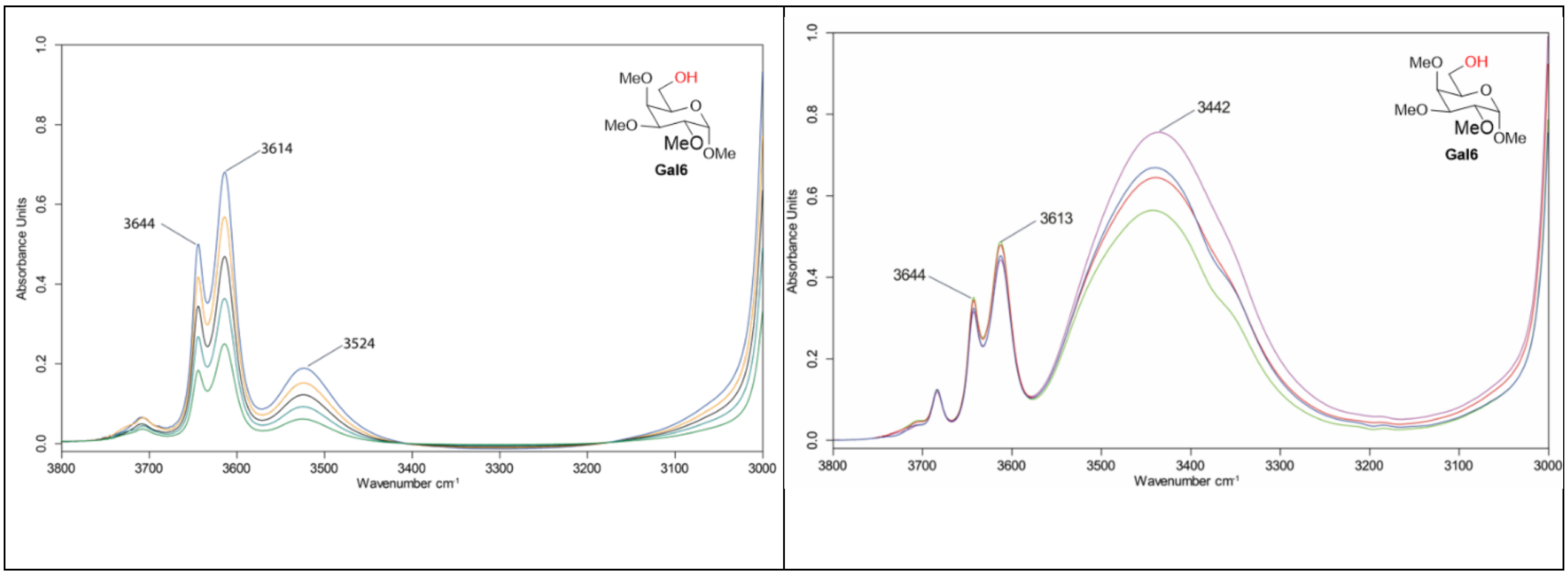
MeO
OH
O
MeO
MeO
OMe
Gal6
3644
3614
3524
Absorbance Units
Wavenumber cm⁻¹
3442
3613
3644
Absorbance Units
Wavenumber cm⁻¹

2 Determination of the exocyclic hydroxylmethyl group conformational populations

We calculate the conformational profile using the limiting *gauche* and *antiperiplanar* *J*-values as determined by Crich et al:[15]

For H6-*R*: $J_{H6R\text{-}H5} = 1.0 \cdot f_{gg} + 11.0 \cdot f_{gt} + 4.8 \cdot f_{tg}$

For H6-*S*: $J_{H6S\text{-}H5} = 2.2 \cdot f_{gg} + 2.5 \cdot f_{gt} + 10.2 \cdot f_{tg}$

With $1 = f_{gg} + f_{gt} + f_{tg}$

2.1 Glc6 ($CDCl_3$)

NMR data:

H6: 3.83 (ddd, *J* = 11.8, 5.1, 2.9 Hz) ppm

H6': 3.75 (ddd, *J* = 11.7, 7.8, 4.2 Hz) ppm

OH: 1.83 (dd, *J* = 7.8, 5.1 Hz) ppm

We don't know which proton is the *pro-R* or *pro-S* one. Hence, there are two possibilities, A and B:

Option A: We take the signal at 3.83 ppm for *pro-R*: $2.9 = 1.0 \cdot f_{gg} + 11.0 \cdot f_{gt} + 4.8 \cdot f_{tg}$

And the signal at 3.75 ppm for *pro-S*: $4.2 = 2.2 \cdot f_{gg} + 2.5 \cdot f_{gt} + 10.2 \cdot f_{tg}$

Which solves as: f_{gg} = 66%; f_{gt} = 10% f_{tg} = 25%:

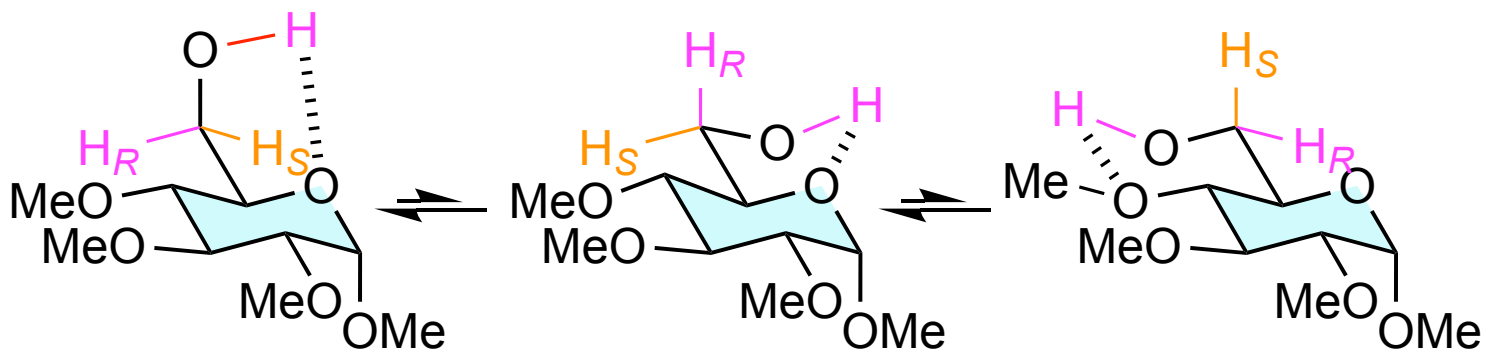

This is unlikely, as the *pro-R* proton, when in the *gg*- and *tg*-conformations, is antiperiplanar to the OH group, but the signal at 3.83 ppm has the smallest $^3J_{H,OH}$-value (5.1 Hz). It is also unlikely that the *tg*-conformation (6-ring IMHB) would be populated to that extent, at least not based on the IR spectrum.

Option B: We take the signal at 3.75 ppm for *pro-R*: $4.2 = 1.0 \cdot f_{gg} + 11.0 \cdot f_{gt} + 4.8 \cdot f_{tg}$

And the signal at 3.83 ppm for *pro-S*: $2.9 = 2.2 \cdot f_{gg} + 2.5 \cdot f_{gt} + 10.2 \cdot f_{tg}$

Which solves as: f_{gg} = 63%; f_{gt} = 29% f_{tg} = 8%:

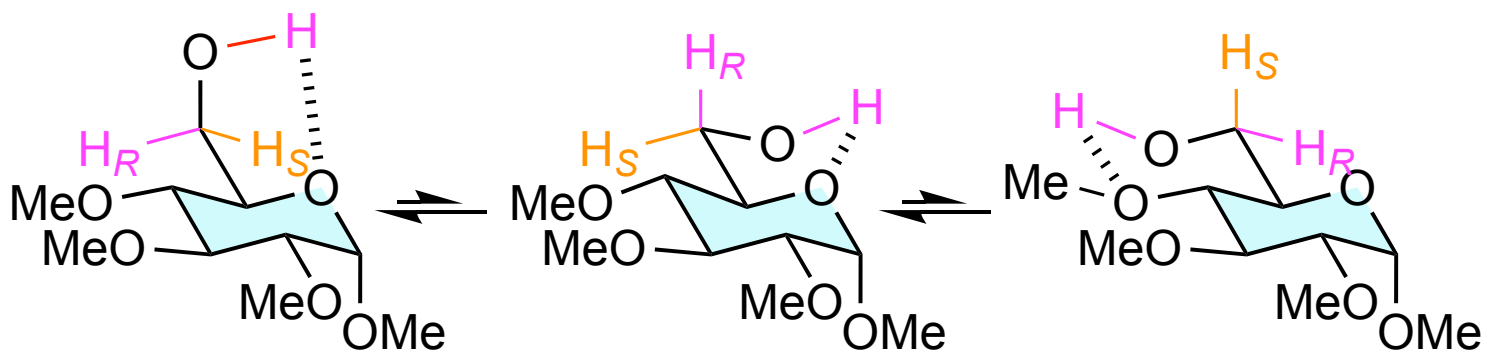

This is a reasonable population distribution, qualitatively consistent with the observed $^3J_{H,OH}$ values, and with the IR spectrum. The small amount of free OH (shoulder at 3640 cm^{-1}) is assumed to also be in the *tg* conformation.

2.2 Gal6 (CCl_4)

NMR data:

H6: 3.91 (ddd, *J* = 10.2, 6.8, 3.0 Hz) ppm

H6': 3.75 (ddd, *J* = 10.6, 4.6, 8.8 Hz) ppm

OH: 1.69 (dd, *J* = 8.9, 3.0 Hz) ppm

We don't know which proton is the *pro-R* or *pro-S* one. Hence, there are two possibilities, A and B:

Option A: We take the signal at 3.91 ppm for *pro-R*: $6.8 = 1.0 \cdot f_{gg} + 11.0 \cdot f_{gt} + 4.8 \cdot f_{tg}$

And the signal at 3.75 ppm for *pro-S*: $4.6 = 2.2 \cdot f_{gg} + 2.5 \cdot f_{gt} + 10.2 \cdot f_{tg}$

Which solves as: f_{gg} = 25%; f_{gt} = 47% f_{tg} = 28%:

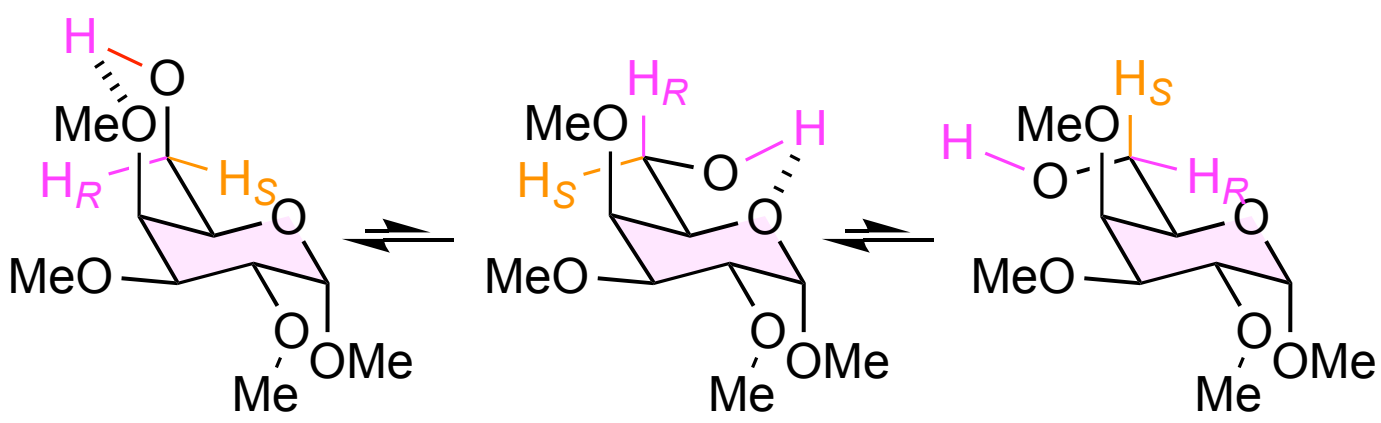

gg (25%) *gt* (47%) *tg* (28%)

This appears consistent with the observed $^3J_{H,OH}$ values, and with the IR spectrum. The *pro-S* proton has a $^3J_{H,OH}$ value of 8.9 Hz with 72% of the population having these protons antiperiplanar, and the *pro-R* proton has a $^3J_{H,OH}$ value of 3.0 Hz with only 28% of the population having these protons antiperiplanar.

Option B: We take the signal at 3.75 ppm for *pro-R*: $4.6 = 1.0 \cdot f_{gg} + 11.0 \cdot f_{gt} + 4.8 \cdot f_{tg}$

And the signal at 3.91 ppm for *pro-S*: $6.8 = 2.2 \cdot f_{gg} + 2.5 \cdot f_{gt} + 10.2 \cdot f_{tg}$

Which solves as: f_{gg} = 29%; f_{gt} = 14% f_{tg} = 57%:

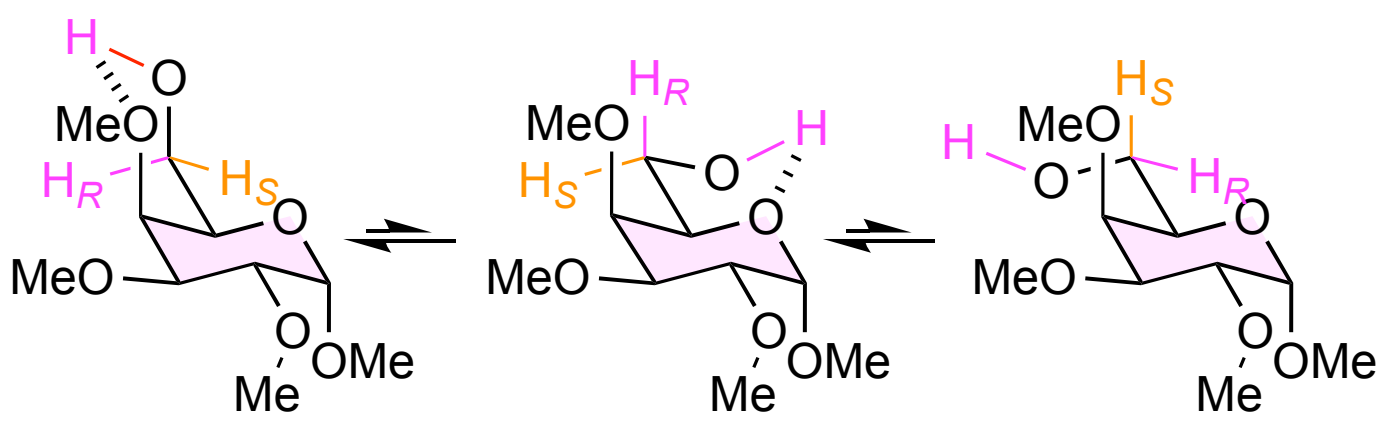

gg (29%) *gt* (14%) *tg* (57%)

This appears less consistent with the observed $^3J_{H,OH}$ values, and not at all with the IR spectrum. It is unlikely that the *pro-R* proton has a $^3J_{H,OH}$ value of 8.9 Hz with only 57% of the population.

2.3 Gal6 ($CDCl_3$)

NMR data:

H6: 3.91 (ddd, J = 10.9, 6.8, 2.5 Hz) ppm

H6': 3.76 (ddd, J = 10.8, 4.7, 8.8 Hz) ppm

OH: 2.09 (dd, J = 8.8, 2.5 Hz) ppm

We don't know which proton is the *pro-R* or *pro-S* one. Hence, there are two possibilities A and B:

Option A: We take the signal at 3.91 ppm for *pro-R*: $6.8 = 1.0 \cdot f_{gg} + 11.0 \cdot f_{gt} + 4.8 \cdot f_{tg}$

And the signal at 3.76 ppm for *pro-S*: $4.7 = 2.2 \cdot f_{gg} + 2.5 \cdot f_{gt} + 10.2 \cdot f_{tg}$

Which solves as: f_{gg} = 24%; f_{gt} = 47% f_{tg} = 29%:

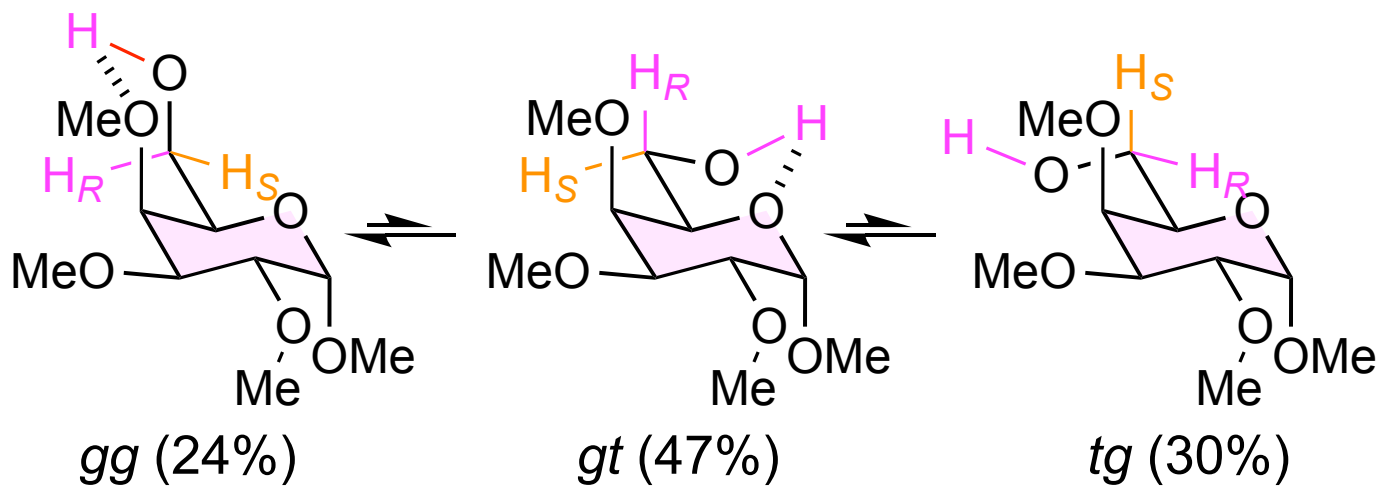

These data are very similar to the data obtained in CCl_4.

Option B: We take the signal at 3.76 ppm for *pro-R*: $4.7 = 1.0 \cdot f_{gg} + 11.0 \cdot f_{gt} + 4.8 \cdot f_{tg}$

And the signal at 3.91 ppm for *pro-S*: $6.8 = 2.2 \cdot f_{gg} + 2.5 \cdot f_{gt} + 10.2 \cdot f_{tg}$

Which solves as: f_{gg} = 28%; f_{gt} = 15% f_{tg} = 57%:

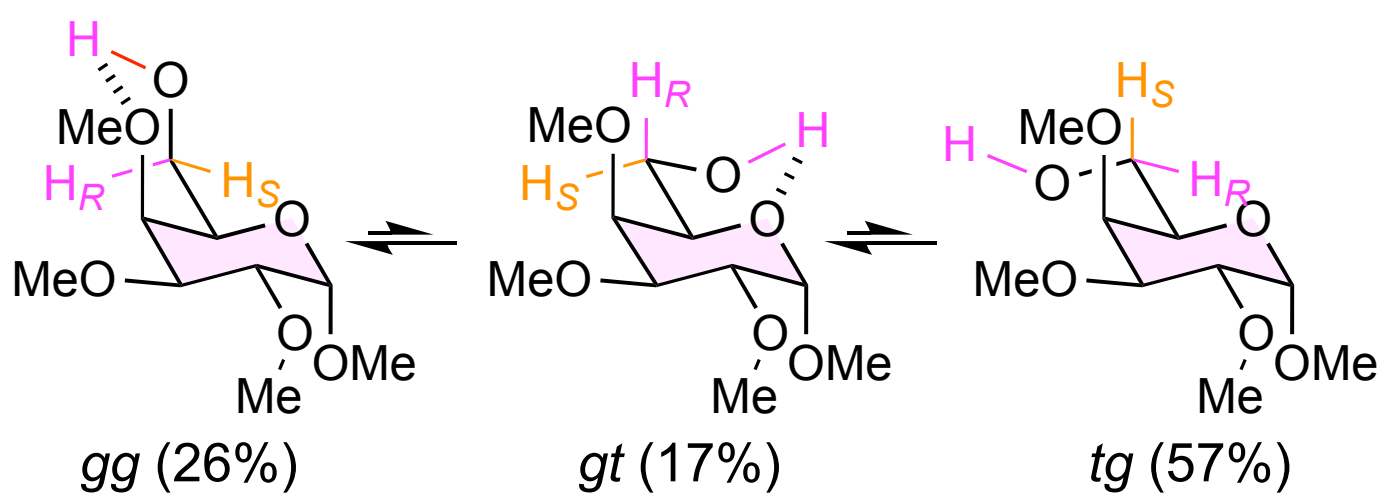

These data are very similar to the data obtained in CCl_4.

2.4 Gal4 ($CDCl_3$)

NMR data:

H6: 3.66 (dd, J = 10.2, 5.2 Hz) ppm

H6': 3.65 (dd, J = 10.1, 6.1Hz) ppm

Option A: We take the signal at 3.66 ppm for *pro-R*: $5.2 = 1.0 \cdot f_{gg} + 11.0 \cdot f_{gt} + 4.8 \cdot f_{tg}$

And the signal at 3.65 ppm for *pro-S*: $6.1 = 2.2 \cdot f_{gg} + 2.5 \cdot f_{gt} + 10.2 \cdot f_{tg}$

Which solves as: f_{gg} = 28%; f_{gt} = 24% f_{tg} = 48%

Option B: We take the signal at 3.65 ppm for *pro-R*: $6.1 = 1.0 \cdot f_{gg} + 11.0 \cdot f_{gt} + 4.8 \cdot f_{tg}$

And the signal at 3.66 ppm for *pro-S*: $5.2 = 2.2 \cdot f_{gg} + 2.5 \cdot f_{gt} + 10.2 \cdot f_{tg}$

Which solves as: f_{gg} = 27%; f_{gt} = 37% f_{tg} = 36%

2.5 Gal3 ($CDCl_3$)

NMR data:

H6: 3.51 (dd, *J* = 9.4, 5.1, 6.3 Hz) ppm

H6’: 3.48 (dd, *J* = 9.6, 7.8, 6.6 Hz) ppm

Option A: We take the signal at 3.51 ppm for *pro-R*: $6.3 = 1.0 \cdot f_{gg} + 11.0 \cdot f_{gt} + 4.8 \cdot f_{tg}$

And the signal at 3.48 ppm for *pro-S*: $6.6= 2.2 \cdot f_{gg} + 2.5 \cdot f_{gt} + 10.2 \cdot f_{tg}$

Which solves as: f_{gg} = 14%; f_{gt} = 32% f_{tg} = 54%

Option A: We take the signal at 3.48 ppm for *pro-R*: $6.6 = 1.0 \cdot f_{gg} + 11.0 \cdot f_{gt} + 4.8 \cdot f_{tg}$

And the signal at 3.51 ppm for *pro-S*: $6.3= 2.2 \cdot f_{gg} + 2.5 \cdot f_{gt} + 10.2 \cdot f_{tg}$

Which solves as: f_{gg} = 13%; f_{gt} = 37% f_{tg} = 50%.

3 DFT calculations

All DFT calculations were carried out using the Gaussian 16 program,[16] applying default procedures, integration grids, algorithms and parameters. We have used the wB97X-D//def2-TZVPD level of theory, recommended for such structural investigations in the case of molecular flexible systems.[17] For each monosaccharide derivative investigated, we selected as starting geometry the one identified by Widmalm and coworkers[18] for comparable protected monosaccharides. From this starting conformation, Larsson and col. considered specific OH rotamers and the theoretical results (B3LYP/6-311G**//B3LYP/6-31G* level of theory) were supported by experimental NMR parameters (spin-spin coupling constants). In the present study, we followed the same methodology by computing the vibrational spectrum for each relevant optimized OH rotamer structure in order to check that a true minimum was found and to calculate the Gibbs free energy G of the corresponding conformer. Solvent effects (CCl_4 herein) were included using an implicit solvent model, namely SMD.[19] The relative populations p_i (Eq. [1]) of the various energy minima taken into account were hence evaluated through a Boltzmann distribution from the corresponding computed Gibbs free energy differences (DG_i).

$$p_i = \frac{e^{-\Delta G_i/RT}}{\sum_{i=1}^{n} e^{-\Delta G_i/RT}} \qquad [1]$$

To help rationalising the predicted trends and the experimental (IR and NMR) results, the molecular dipole moment of the various conformers considered was also reported and analysed. In addition, the $V_\alpha(r)$ values of the free alcohols were calculated. This value, also called the Kenny parameter,[20] is a predictor of the HB donating capacity (pK_{AHY} scale).[5],[2] The $V_\alpha(r)$ value is calculated for every conformer, and is the weighted sum of the conformational values. The larger the $V_\alpha(r)$ value, the larger HB donating capacity. We have shown that conformational $V_\alpha(r)$ values can be used to evaluate the HB donating capacity of individual conformers. Of relevance here is that the Kenny parameter also correlates with intramolecular hydrogen bonding: the stronger the IMHB, the lower the $V_\alpha(r)$ value.[4]

The free energies, dipole moments and $V_\alpha(r)$ values of selected conformers for a number of sugar derivatives are discussed below.

3.1 Table S3. 3D-structures of the different major conformers of Glc2 in CCl_4.

Glc2

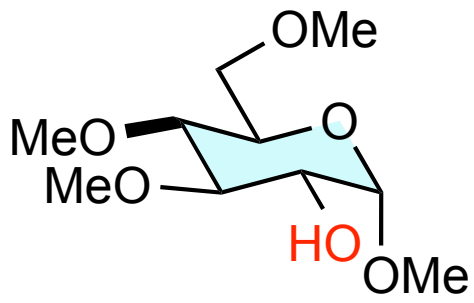

Glc2_3: Glc2-(*g*+)-(5-*c*-IMHB)[a,b]	Glc2_1: Glc2-(*t*)-(5-*c*-IMHB)[a,b]	Glc2_2: Glc2-(*g*+)-(5-*t*-IMHB)[a,b]	Glc2_4: Glc2-(*t*)-(5-*t*-IMHB)[a,b]
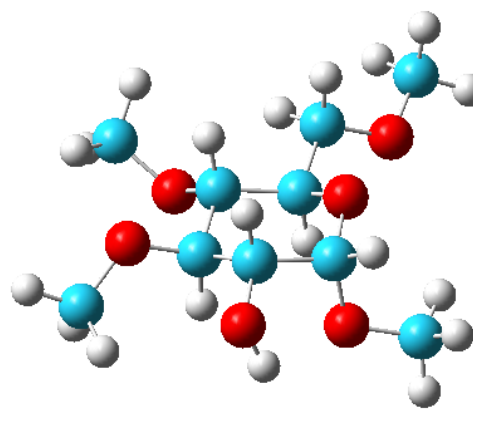	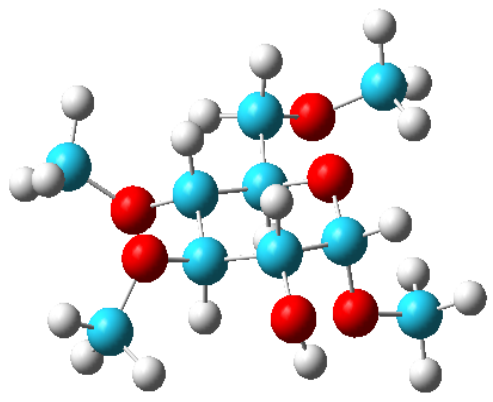	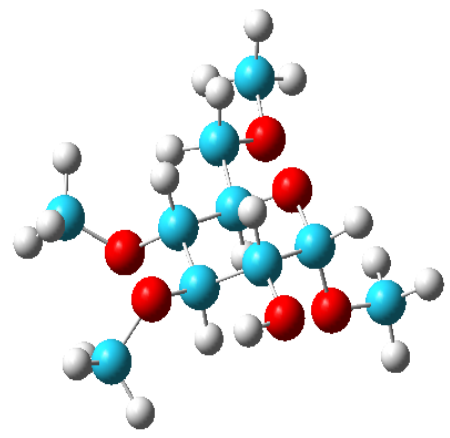	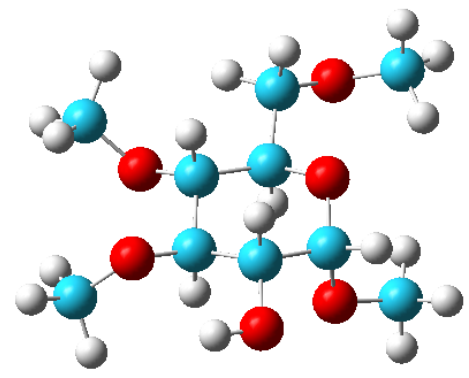
Vα=0.3035	Vα=0.3069	Vα=0.3057	Vα=0.3078
d(O2H…O1)=2.225 (Å)<br>1,2 cis 5 ring IMHB<br>The most stable conformer found<br>μ=1.73 D	*d*(O2H…O1)=2.236 (Å)<br>1,2 cis 5 ring IMHB<br>Δ*G* = +3.7 kJ/mol above the most stable conformer found<br>μ=0.87 D	*d*(O2H…O3)=2.360 (Å)<br>1,2 trans 5 ring IMHB<br>Δ*G* = +5.9 kJ/mol above the most stable conformer found<br>μ=3.05 D	*d*(O2H…O3)=2.360 (Å)<br>1,2 trans 5 ring IMHB<br>Δ*G* = +8.3 kJ/mol above the most stable conformer found<br>μ=1.79 D

[a] Descriptors *g*, *t* refer to dihedral angle between carbinol C–H and O–H bonds. [b] IMHB descriptors *c, t* refer to ring size and fused bond stereochemistry.

The IMHB conformers of Glc2 (5-*c*-IMHB and 5-*t*-IMHB) were calculated for the two minimum energy exocyclic hydroxymethyl conformers (*g*+ and *t*). Clearly for both hydroxymethyl conformers the Glc2 (5-*c*-IMHB) appears as the preferred conformer, in agreement with the larger intensity of the absorption band in the IR spectrum, and the observed $^{3}J_{H2,OH}$ coupling constant. The V_{α}(r) values of the alcohol group in the 5-*c*-IMHB conformation vary rather considerably depending on the hydroxymethyl conformation (0.3035 vs 0.3069), but importantly, for both these conformers the V_{α}(r) value is lower than that of their corresponding 5-*t*-IMHB conformers (0.3035 vs 0.3057 and 0.3069 vs 0.3078), which indicates that the 5-*c*-IMHB is stronger.[4] This agrees with the position, at lower frequency compared to other 5-IMHB rings, of the main absorption band seen in the IR spectrum.

3.2 Table S4. 3D-structures of the different major conformers of Gal3 in CCl_4.

Gal-3

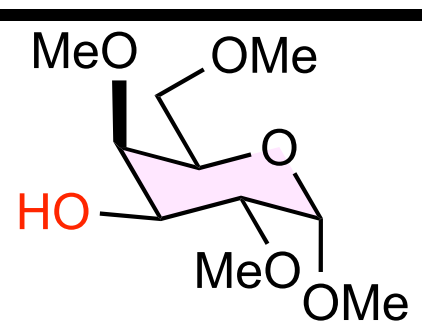

Gal3_1: Gal3-(*g*+)-(5-*t*-IMHB)[a,b]	Gal3_2: Gal3-(*t*)-(5-*c*-IMHB)[a,b]
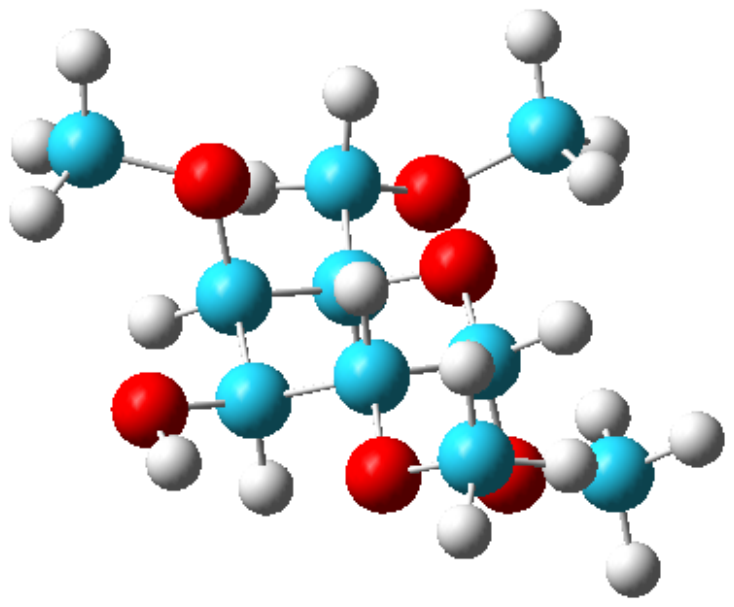	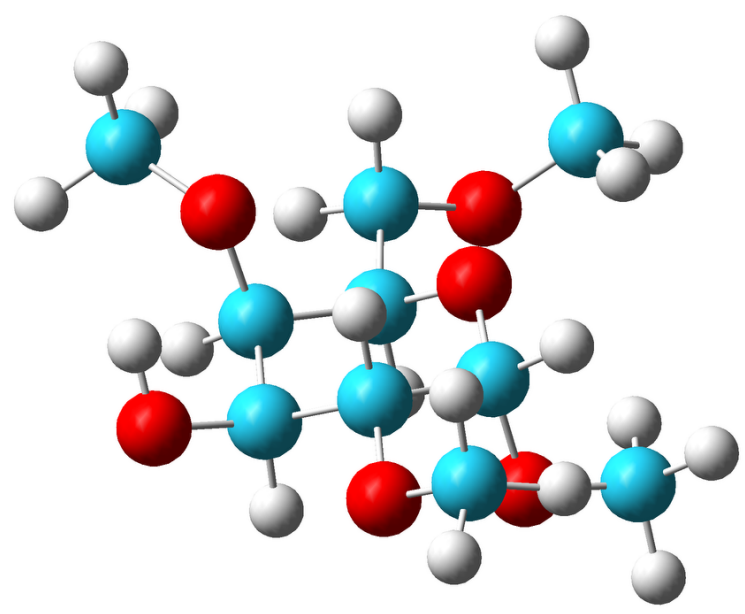
Vα=0.3025 (pK_{AHY} = -0.11)	Vα=0.3000 (pK_{AHY} = -0.24)
d(O3H…O2)=2.266(Å)<br>1,2 trans 5 ring IMHB<br>Most stable conformer<br>μ=2.06 D	*d*(O3H…O4)=2.221 (Å)<br>1,2 cis 5 ring IMHB<br>Δ*G* = +1,6 kJ/mol above the most stable conformer found<br>μ=2.98 D

[a] Descriptors *g*, *t* refer to dihedral angle between carbinol C–H and O–H bonds. [b] IMHB descriptors *c*, *t* refer to ring size and fused bond stereochemistry.

The relative energy of the 5-*t*-IMHB conformer is lower than that of the 5-*c*-IMHB conformer. This is consistent with the larger IR absorption band of the 5-*t*-IMHB conformer, and with the observed $^3J_{H3,OH}$ value.

However, the $V_\alpha(r)$ value for the 5-*c*-IMHB is lower than that of the 5-*t*-IMHB which does indicate that it is a stronger IMHB.[4] Hence IMHB strength cannot be the reason for the lower abundance of the Gal3-(*t*) conformer. Its larger dipole moment compared to the most stable Gal3-(*g*+) conformer might contribute to its destabilisation in CCl_4.

3.3 Table S5. 3D-structures of the different major conformers of Gal4 in CCl_4.

**Gal4**	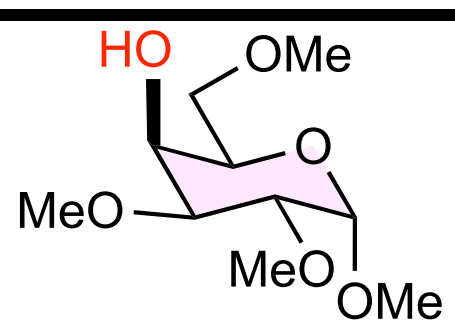	
Gal4_1: Gal4-(*g*+)-(5-*c*-IMHB)-(*gt*)[a,b,c]	Gal4_2: Gal4-(*t*)-(5-*c*-IMHB)-(*gt*)[a,b,c]	Gal4_3: Gal4-(*g*-)-(6-IMHB)-(*gg*)[a,c]
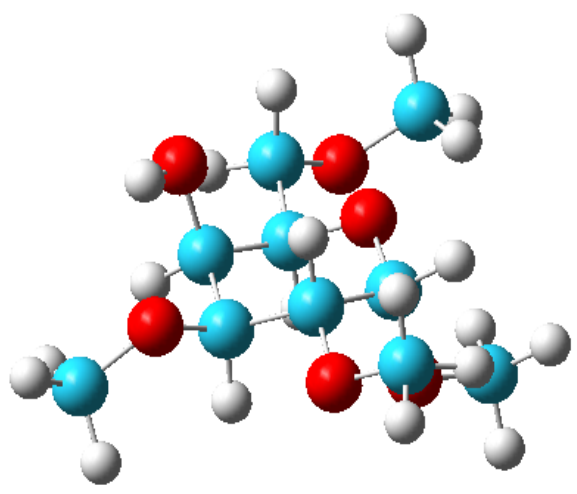	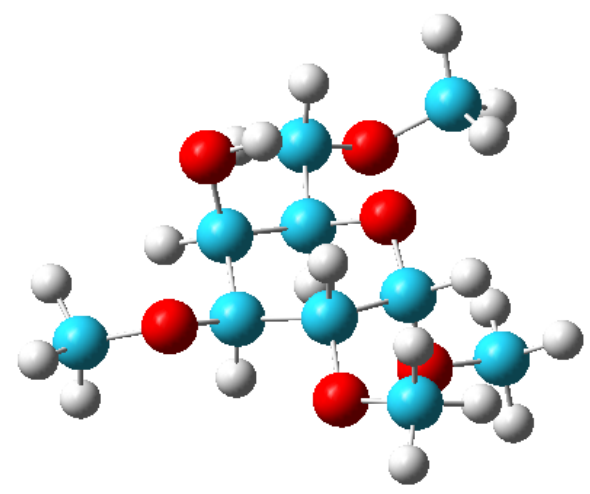	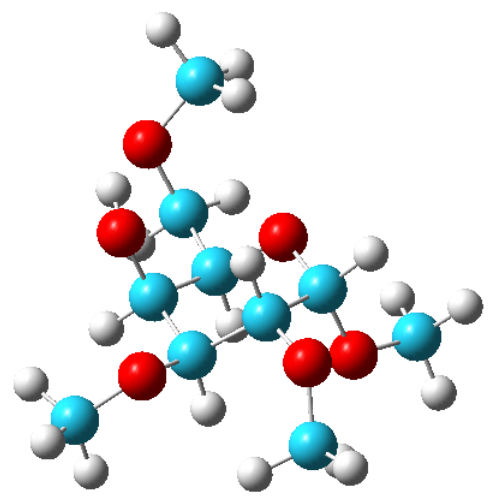
Vα=0.2977	Vα=0.3162	Vα=0.2717
d(O4H…O3)=2.135 (Å)<br>1,2 cis 5 ring IMHB<br>Most stable conformer<br>μ=1.46 D	*d*(O4H…O5)=2.527 (Å)<br>1,2 cis 5 ring IMHB<br>Δ*G* = +9.6 kJ/mol above the most stable conformer<br>μ=2.26 D	*d*(O4H…O6)=1.911 (Å)<br>6 ring IMHB<br>Δ*G* = +13.0 kJ/mol above the most stable conformer<br>μ=4.82 D

[a] Descriptors *g*, *t* refer to dihedral angle between carbinol C–H and O–H bonds. [b] IMHB descriptors *c, t* refer to ring size and fused bond stereochemistry. [c] C5–C6 conformational descriptors *gg, tg, gt* as conventionally defined.

The Gal4-(*g*+) is by far the most stable conformer. This is fully consistent with the IR spectrum, where the absorption bands of the other conformers have a much lower intensity. It is also fully consistent with the observed coupling constants in the ^{1}H NMR spectrum: the $^3J_{H4,OH}$ value is 1.2 Hz, indicating a dihedral angle even less than the *gauche*-angle, and the presence of a $^4J_{H5,OH}$ value, indicating a rigid situation.

The $V_\alpha(r)$ values indeed show that the 5-*c*-IMHB to 3-OMe is much stronger than the potential 5-*c*-IMHB to the ring oxygen. The latter is an acetal oxygen involved in the anomeric hyperconjugation and hence a less strong HB acceptor. The 6-IMHB is the strongest IMHB, as revealed by the very low $V_\alpha(r)$ value, but the high dipole moment of this conformer won't be stabilised in CCl_4. In addition, there is electronic repulsion between the parallel C4-OH and C6-CO bonds.

3.4 Table S6. 3D-structures of the different major conformers of Gal6 in CCl_4.

**Gal6**	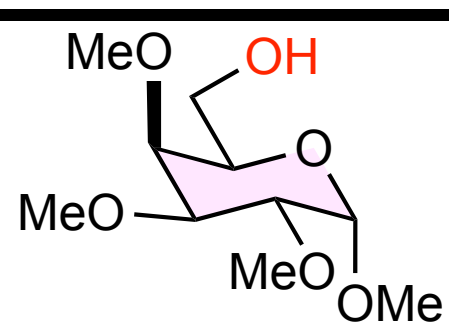		
Gal6_1:<br>Gal6-(*g*-)-(5-IMHB)-(*gt*)[c,d]	Gal6_2:<br>Gal6-(*g*+)-free-(*tg*)[c,d]	Gal6_3:<br>Gal6-(*g*-)-(6-ring)-(*gg*)[c,d]	Gal6_4:<br>Gal6-(*g*-)-free-(*tg*)[c,d]
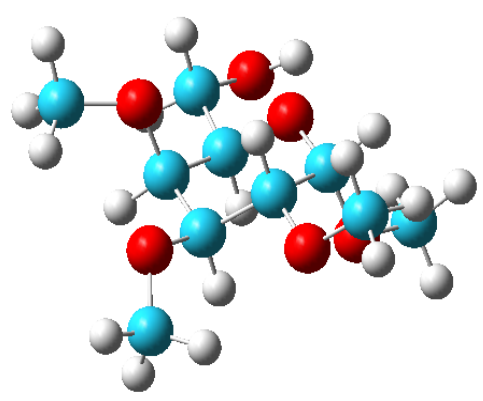	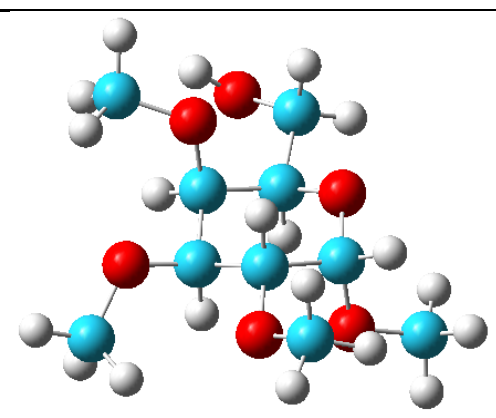	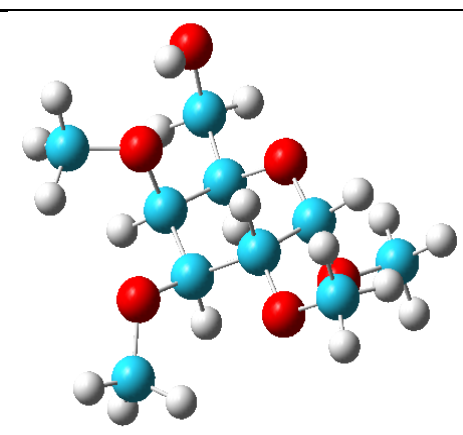	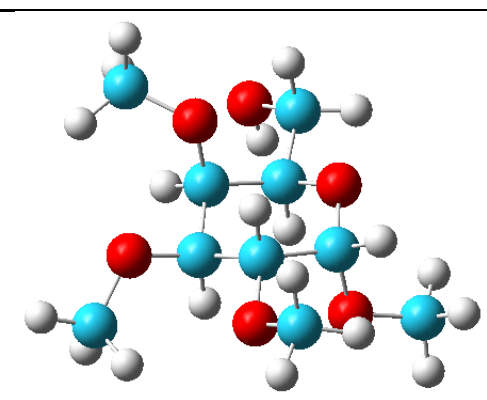
Vα=0.3034	Vα=0.3157	Vα=0.2651	Vα=0.3234
d(O6H…O5)=2.391 (Å)<br>5 ring IMHB<br>Most stable conformer<br>μ=1.74 D	*d*(O6H…O4)=2.747 (Å)<br>"Free OH"<br>Δ*G* = +5.2 kJ/mol above the most stable conformer<br>μ=1.64 D	*d*(O6H…O4)=1.931 (Å)<br>6 ring IMHB<br>Δ*G* = +5.7 kJ/mol above the most stable conformer<br>μ=3.07 D	-<br>Free OH<br>Δ*G* = +5.7 kJ/mol above the most stable conformer<br>μ=2.69 D

[c] C5–C6 conformational descriptors *gg, tg, gt* as conventionally defined. [d] Descriptors *g*, *t* refer to the dihedral angle between C5–C6 and O–H bonds.

The *gt*-conformer is calculated to be the most stable one, which corresponds to the NMR data (47% population based on $^3J_{H5,H6}$ values. The *tg* and *gg*-conformers have similar destabilisations, and indeed similar populations were calculated by NMR. Interestingly, there are two *tg*-conformations of similar destabilisation that differ in the orientation of the O–H bond (see below).

The V_α(r) value for the *gg*-conformer again reveals the strong 6-ring IMHB. Interestingly, the value for the *gt* conformer also indicates a good IMHB, despite an acetal oxygen being involved. Possible explanations include: as it is a primary alcohol, the OH group may be a stronger donor. The conformational flexibility may also allow a more favourable geometry for optimal orbital overlap. Finally, it is the equatorial alcohol which is involved in this IMHB, which is not involved in the anomeric hyperconjugation.

The V_α(r) values of the two *tg*-conformers are very different. However, it can be seen that the

O–H group is pointing towards O4 in the Gal6_2 conformer, suggesting an electrostatic interaction. The alcohol hydrogen atom of Gal6_4 is pointing away towards space. This alcohol group displays a very large $V_{\alpha}(r)$ value, as expected due to its antiperiplanar disposition with the ring oxygen. Due to the electrostatic interaction with O4, the $V_{\alpha}(r)$ value of the OH group of Gal6_2 is reduced compared to that of Gal6_4.

3.5 Table S7. 3D-structures of the different major conformers of Glc6 in CCl_4.

**Glc6**	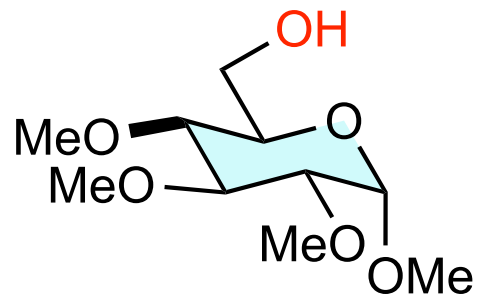	
Glc6_1 : Glc6-(*g*+)-(5-IMHB)-(*gg*)[c,d]	Glc6_2: Glc6-(*g*+)-free-(*gt*)[c,d]	Glc6_3: Glc6-(*g*+)-(6-IMHB)-(*tg*)[c,d]
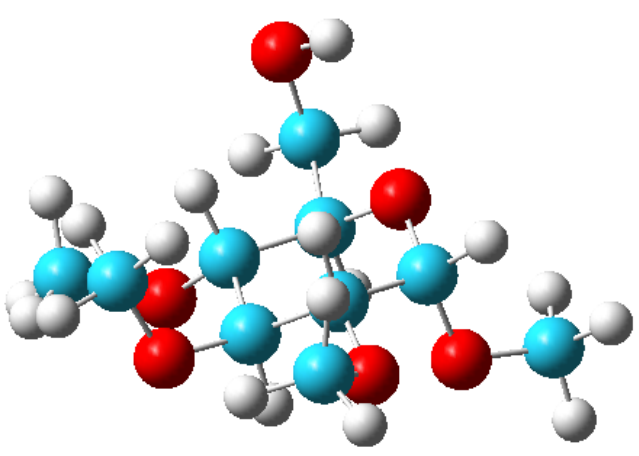	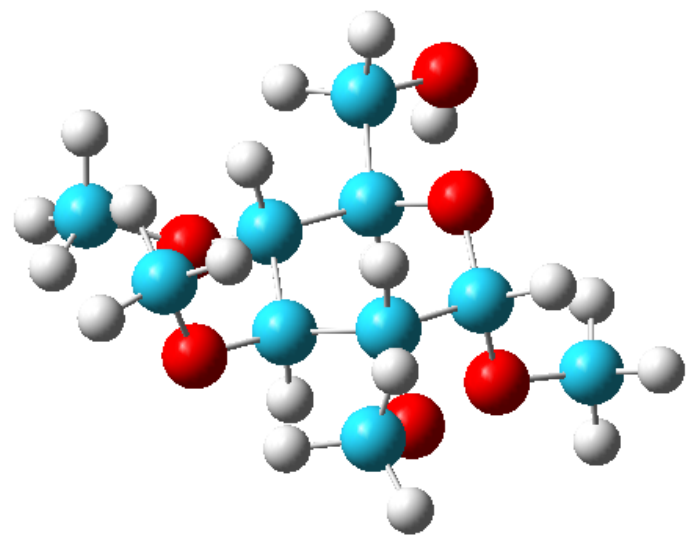	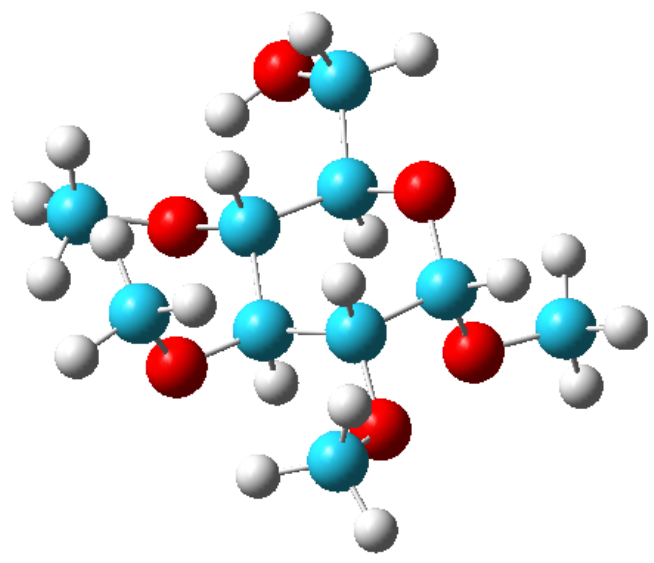
Vα=0.3068	Vα=0.3171	Vα=0.2772
d(O6H…O5) = 2.374 (Å)<br>5 ring IMHB<br>Most stable conformer<br>μ=2.07 D	-<br>Free OH<br>Δ*G* =+7.6 kJ/mol above the most stable conformer<br>μ=2.43 D	*d*(O6H…O4)=1.991 (Å)<br>6 ring IMHB<br>Δ*G* =+7.8 kJ/mol above the most stable conformer<br>μ=3.92 D

[c] C5–C6 conformational descriptors *gg, tg, gt* as conventionally defined. [d] Descriptors *g*, *t* refer to the dihedral angle between C5–C6 and O–H bonds.

The Glc6 *gt* alcohol is involved in a 5-ring IMHB with the anomeric oxygen, which is, as said above, a worse HB acceptor. However, its $V_{\alpha}(r)$ value is larger than the value of the Gal6_1 conformer, which is also involved in a 5-ring IMHB to the endocyclic oxygen. We suggest this is consistent with the notion that the axial oxygen lone pair is a worse acceptor than the equatorial lone pair.

The $V_\alpha(r)$ value of the 6-OH is larger than that of the 6-OH group of galactose, which means that the 6-ring IMHB in glucose is weaker than that in galactose. While in glucose, the 6-OH is antiperiplanar with the ring oxygen, and thus a better HB donor, the same is true for the 4-OMe HB acceptor oxygen. The latter appears to be the larger effect.

3.6 Structural coordinates, total energies and dipole moment for Glc 2, Gal3, Gal4, Gal6 and Glc6.

3.6.1 Glc2_3: Glc2-(*g*+)-(5-*c*-IMHB)[a,b]

SCF energy (Hartree) = -844.237293, Dipole Moment (Debye)= 1.7307

C	-0.210524	-1.062291	-0.186762
C	0.998729	-0.149736	0.029133
O	0.903395	0.997024	-0.804846
C	-0.221383	1.792569	-0.585502
C	-1.514120	1.007595	-0.804071
C	-1.508831	-0.286211	0.010006
C	2.294296	-0.846384	-0.307309
O	3.372464	-0.096001	0.185949
C	4.612304	-0.672082	-0.121546
O	-0.278274	2.314228	0.710305
C	0.750404	3.237618	1.006257
O	-2.639304	1.809114	-0.531842
O	-2.584178	-1.114437	-0.371436
C	-3.669947	-1.133031	0.523490
O	-0.138398	-2.124937	0.736248
C	-0.422187	-3.397676	0.204711
H	0.568170	3.606373	2.014460
H	0.730997	4.080524	0.305872
H	1.734879	2.764584	0.966890
H	-0.142436	2.607789	-1.313956
H	-1.568515	0.742584	-1.862048
H	-1.584507	-0.037886	1.075986
H	2.286269	-1.846510	0.144487
H	2.369712	-0.960859	-1.398648
H	1.026184	0.152033	1.082178
H	-3.362920	-1.491441	1.513689
H	-4.129451	-0.145014	0.625264
H	-4.408899	-1.823229	0.115689
H	4.707565	-1.679320	0.306463
H	4.768025	-0.740093	-1.206555
H	5.387242	-0.035188	0.304532
H	-0.184367	-1.446565	-1.215230
H	-0.319492	-4.113957	1.020025
H	-1.438690	-3.451501	-0.195196
H	0.286548	-3.666702	-0.589008
H	-2.464413	2.262213	0.299650

3.6.2 Glc2_1: Glc2-(*t*)-(5-*c*-IMHB)[a,b]

SCF energy (Hartree) = -844.235884, Dipole Moment (Debye)= 0.8669

C	-0.500102	-1.061325	-0.167119
C	0.918496	-0.552715	0.098920
O	1.195335	0.572564	-0.728036
C	0.352228	1.668912	-0.533890
C	-1.105964	1.305106	-0.807013
C	-1.515815	0.066376	-0.010622
C	1.960805	-1.615245	-0.192874
O	3.230903	-1.333825	0.338116
C	4.051272	-0.515217	-0.460897
O	0.408563	2.176816	0.766754
C	1.665827	2.710388	1.126609
O	-1.951237	2.404468	-0.564330
O	-2.772854	-0.401974	-0.444615
C	-3.845788	-0.122119	0.421738
O	-0.780783	-2.094454	0.749180
C	-1.419185	-3.223942	0.200852

```
H      1.549789     3.170071     2.106780
H      1.989062     3.472015     0.407746
H      2.429757     1.929924     1.185823
H      0.697924     2.426403    -1.246701
H     -1.197098     1.071104    -1.869712
H     -1.557395     0.324409     1.054801
H      1.629913    -2.542954     0.275679
H      2.019128    -1.775592    -1.278469
H      0.997330    -0.269742     1.154645
H     -3.691463    -0.578638     1.407050
H     -3.998453     0.954781     0.544314
H     -4.740616    -0.553764    -0.027301
H      4.150759    -0.922498    -1.475330
H      3.672283     0.507842    -0.535530
H      5.036323    -0.500124     0.006439
H     -0.552563    -1.438682    -1.197339
H     -1.558342    -3.936902     1.013568
H     -2.393511    -2.970508    -0.226323
H     -0.802224    -3.694277    -0.575304
H     -1.690658     2.777053     0.284371
```

3.6.3 Glc2_2: Glc2-(*g*+)-(5-*t*-IMHB)[a,b]

```
SCF energy (Hartree) = -844.235031, Dipole Moment (Debye)= 3.0506

C     -0.276337    -0.998884    -0.196552
C      0.972896    -0.138723     0.030742
O      0.923447     1.031488    -0.773134
C     -0.179872     1.867337    -0.526965
C     -1.460417     1.101754    -0.838709
C     -1.530582    -0.158187     0.012010
C      2.235257    -0.883035    -0.329982
O      3.347509    -0.194947     0.176792
C      4.559853    -0.810810    -0.160950
O     -0.225884     2.321903     0.782171
C      0.832705     3.192801     1.119907
O     -2.578048     1.928324    -0.648269
O     -2.701415    -0.858714    -0.365180
C     -3.424419    -1.426341     0.705568
O     -0.256694    -2.088035     0.701446
C     -0.469711    -3.345890     0.107469
H      0.658231     3.523198     2.142799
H      0.845272     4.068044     0.459382
H      1.803475     2.692223     1.063005
H     -0.057836     2.706827    -1.221272
H     -1.386766     0.799175    -1.891935
H     -1.593582     0.129627     1.067070
H      2.186787    -1.893795     0.096130
H      2.301229    -0.972634    -1.424292
H      1.017139     0.133301     1.091265
H     -2.824019    -2.146536     1.268709
H     -3.782558    -0.653521     1.395857
H     -4.284372    -1.938434     0.273696
H      4.616190    -1.835582     0.231110
H      4.704998    -0.847128    -1.248973
H      5.363638    -0.221907     0.280404
H     -0.272151    -1.362869    -1.232122
H     -0.448877    -4.086732     0.906750
H     -1.438232    -3.398320    -0.401859
H      0.318602    -3.588621    -0.616060
H     -3.354728     1.373395    -0.765518
```

3.6.4 Glc2_4: Glc2-(*t*)-(5-*t*-IMHB)[a,b]

```
SCF energy (Hartree) = -844.234128, Dipole Moment (Debye)= 1.7947

C      0.569637     0.972817    -0.182018
C     -0.880880     0.554618     0.091295
O     -1.226377    -0.573612    -0.704560
C     -0.436937    -1.716736    -0.481153
C      1.009322    -1.395579    -0.836510
C      1.500063    -0.221292    -0.001828
C     -1.854058     1.672830    -0.231414
O     -3.137871     1.494820     0.309635
C     -4.005608     0.684758    -0.447373
O     -0.498402    -2.160675     0.830694
C     -1.775086    -2.615748     1.221111
```

O	1.812338	-2.533335	-0.668092
O	2.819842	0.070254	-0.421822
C	3.717319	0.390709	0.619291
O	0.925011	2.012510	0.703884
C	1.489713	3.145426	0.088393
H	-1.669118	-3.047653	2.215098
H	-2.145895	-3.386079	0.534412
H	-2.502366	-1.798856	1.262519
H	-0.840218	-2.475061	-1.162498
H	1.003995	-1.086480	-1.890413
H	1.502183	-0.513041	1.054096
H	-1.464115	2.592124	0.208045
H	-1.905491	1.802760	-1.321647
H	-0.975768	0.307495	1.154667
H	3.395418	1.272424	1.180539
H	3.829207	-0.447145	1.317429
H	4.682838	0.594533	0.156309
H	-4.071415	1.035681	-1.485343
H	-3.694283	-0.363286	-0.456646
H	-4.992223	0.762628	0.010318
H	0.647599	1.318131	-1.221207
H	1.725768	3.856876	0.879589
H	2.408298	2.899055	-0.455502
H	0.786824	3.617495	-0.609180
H	2.721522	-2.252099	-0.807261

3.6.5 Gal3_1: Gal3-(*g*+)-(5-*t*-IMHB)[a,b]

SCF energy (Hartree) = -844.233155, Dipole Moment (Debye)= 2.0580

C	0.113763	1.422576	-0.542475
C	1.125958	0.284370	-0.422576
O	0.748873	-0.627220	0.604790
C	-0.491638	-1.251187	0.410131
C	-1.593663	-0.188826	0.303006
C	-1.261320	0.809117	-0.795682
C	2.525184	0.782594	-0.115409
O	3.521783	-0.181129	-0.358410
C	3.821649	-1.016445	0.734324
O	-0.523893	-2.050860	-0.725855
C	0.385108	-3.128931	-0.687689
O	-2.867222	-0.705142	0.010157
C	-3.438698	-1.479529	1.035507
O	-2.237303	1.822494	-0.870891
O	0.154181	2.197308	0.633221
C	-0.049392	3.578375	0.444173
H	0.182046	-3.748753	-1.559812
H	0.245676	-3.730747	0.218960
H	1.423479	-2.785907	-0.725815
H	-0.626334	-1.866444	1.308493
H	-4.477219	-1.662610	0.761308
H	-2.934818	-2.445821	1.147262
H	-3.413351	-0.951564	1.996895
H	-1.616723	0.342113	1.263565
H	-1.211397	0.264253	-1.746753
H	2.739044	1.625017	-0.778266
H	2.569288	1.143397	0.917394
H	4.120884	-0.429166	1.611994
H	4.660382	-1.645171	0.433822
H	2.976665	-1.651948	1.014040
H	1.157401	-0.243202	-1.382943
H	0.387195	2.038843	-1.408328
H	-3.093553	1.385850	-0.832049
H	0.054891	4.050751	1.421167
H	0.706785	4.001157	-0.230042
H	-1.043396	3.793666	0.045154

3.6.6 Gal3_2: Gal3-(*t*)-(5-*c*-IMHB)[a,b]

SCF energy (Hartree) = -844.232546, Dipole Moment (Debye)= 2.9779

C	0.254451	1.393287	-0.574834
C	1.090473	0.122846	-0.450335
O	0.593011	-0.703449	0.591799
C	-0.723063	-1.146911	0.391164
C	-1.683272	0.051219	0.305502
C	-1.224190	1.039480	-0.765736

```
C      2.560249     0.382333    -0.179761
O      3.359689    -0.760902    -0.356997
C      3.561520    -1.535987     0.801694
O     -0.861714    -1.915266    -0.757403
C     -0.123953    -3.117320    -0.729655
O     -3.000473    -0.319287     0.018782
C     -3.634179    -1.062231     1.026253
O     -2.003289     2.208791    -0.775664
O      0.345474     2.215338     0.577040
C      1.144081     3.366353     0.430373
H     -0.393230    -3.678013    -1.623591
H     -0.376851    -3.713657     0.155771
H      0.954753    -2.932696    -0.733183
H     -0.938815    -1.753046     1.279516
H     -4.692876    -1.111042     0.772299
H     -3.249017    -2.086778     1.093298
H     -3.530193    -0.581344     2.007984
H     -1.634839     0.550883     1.284663
H     -1.352078     0.568314    -1.741810
H      2.914617     1.124001    -0.900598
H      2.685565     0.794286     0.828729
H      4.022256    -0.941011     1.600746
H      4.244978    -2.340804     0.529981
H      2.629578    -1.965487     1.178549
H      1.017646    -0.410619    -1.406081
H      0.585683     1.954171    -1.456179
H     -1.738712     2.718014    -0.002021
H      1.084187     3.919528     1.367491
H      2.193759     3.119800     0.238409
H      0.779297     4.005279    -0.382406
```

3.6.7 Gal4_1: Gal4-(*g*+)-(5-*c*-IMHB)-(*gt*)[a,b,c]

```
SCF energy (Hartree) = -844.233494, Dipole Moment (Debye)= 1.4569

C     -1.360843    -0.710311     0.360798
C     -0.086005    -1.540574     0.154367
C      1.149366    -0.661044     0.267990
O      1.058877     0.465738    -0.593263
C     -0.027769     1.307358    -0.323366
C     -1.352553     0.546113    -0.494794
O     -0.113228    -2.174534    -1.104903
C      2.429401    -1.407041    -0.060564
O      3.586356    -0.737459     0.380684
C      4.139628     0.169119    -0.543427
O      0.012248     1.847690     0.956780
C      1.131893     2.673572     1.189602
O     -2.474437     1.307413    -0.149096
C     -2.733719     2.390296    -1.003823
O     -2.484833    -1.483078    -0.008974
C     -2.992126    -2.296286     1.023287
H      0.993788     3.130448     2.168483
H      1.202410     3.464566     0.432819
H      2.065708     2.102918     1.188933
H      0.056644     2.104460    -1.072196
H     -3.720569     2.774055    -0.746019
H     -2.006093     3.201079    -0.880163
H     -2.741389     2.079345    -2.056583
H     -1.404612     0.250664    -1.551740
H     -3.332495    -1.689341     1.869581
H     -2.253100    -3.022254     1.382618
H     -3.842523    -2.840924     0.614459
H     -1.433987    -0.411673     1.412333
H      2.407345    -2.367291     0.460534
H      2.475720    -1.606402    -1.136378
H      4.374404    -0.327233    -1.493777
H      5.066846     0.542151    -0.107307
H      3.472501     1.011414    -0.747257
H      1.216601    -0.320869     1.308274
H     -1.032650    -2.413476    -1.267946
H     -0.028046    -2.294997     0.951092
```

3.6.8 Gal4_2: Gal4-(*t*)-(5-*c*-IMHB)-(*gt*)[a,b,c]

```
SCF energy (Hartree) = -844.229806, Dipole Moment (Debye)= 2.2619

C     -0.139682    -1.560008     0.106126
```

```
C      1.094763    -0.682878     0.273175
O      1.012994     0.406793    -0.643762
C     -0.037411     1.298046    -0.356573
C     -1.392465     0.573104    -0.447729
C     -1.397373    -0.709143     0.376792
C      2.395200    -1.420738     0.021833
O      3.523326    -0.700403     0.457055
C      4.116313     0.122517    -0.519247
O      0.081083     1.878711     0.896178
C      1.236401     2.675428     1.048311
O     -2.463774     1.365304    -0.027593
C     -2.730257     2.473105    -0.847148
O     -2.586400    -1.385139     0.076819
C     -2.857597    -2.488430     0.899805
O     -0.168702    -2.198492    -1.151871
H      1.147496     3.185325     2.006314
H      1.306083     3.423850     0.249362
H      2.148982     2.071589     1.046842
H      0.041308     2.063793    -1.138177
H     -3.684982     2.888016    -0.525204
H     -1.966064     3.253732    -0.752785
H     -2.812198     2.182283    -1.902765
H     -1.526294     0.301424    -1.506533
H     -2.776789    -2.226765     1.963176
H     -2.194610    -3.336046     0.691676
H     -3.882524    -2.796859     0.694630
H     -1.381333    -0.428750     1.440287
H      2.372689    -2.351222     0.594604
H      2.483409    -1.682687    -1.038200
H      4.436245    -0.463580    -1.390462
H      4.997187     0.574687    -0.062818
H      3.441369     0.913135    -0.859461
H      1.118319    -0.297247     1.297820
H     -0.086246    -1.529871    -1.837502
H     -0.097608    -2.365023     0.842525
```

3.6.9 Gal4_3: Gal4-(*g*-)-(6-IMHB)-(*gg*)[a,c]

```
SCF energy (Hartree) = -844.228525, Dipole Moment (Debye)= 4.8219

C     -1.171345     0.139267    -0.756739
O     -1.002196     1.070765     0.300443
C      0.340012     1.420970     0.529365
C      1.184714     0.184652     0.863597
C      1.058213    -0.863717    -0.236850
C     -0.428293    -1.169796    -0.474211
O      0.893923     2.080843    -0.567199
C      0.347948     3.360172    -0.801961
O      2.496147     0.541163     1.222841
C      3.466703     0.644220     0.204784
O      1.799324    -1.986662     0.156195
C      1.908452    -2.986276    -0.821949
C     -2.659088    -0.039939    -1.010561
O     -3.363806    -0.741527    -0.005171
C     -3.808797     0.050512     1.080833
H      0.906152     3.800851    -1.626894
H      0.452727     4.000262     0.081881
H     -0.710441     3.306391    -1.074125
H      0.316670     2.089545     1.396825
H      4.374039     1.004806     0.689755
H      3.681386    -0.330395    -0.245464
H      3.179563     1.353647    -0.574663
H      0.746425    -0.244876     1.766203
H      2.246519    -2.574978    -1.783183
H      0.963481    -3.518599    -0.977951
H      2.650805    -3.701539    -0.468462
H      1.459802    -0.466779    -1.181069
H     -3.113260     0.944388    -1.172849
H     -2.776660    -0.623501    -1.926442
H     -2.975059     0.422519     1.680132
H     -4.444251    -0.586631     1.695285
H     -4.399220     0.901361     0.722843
H     -0.754466     0.557369    -1.683235
O     -0.926891    -1.864867     0.641305
H     -1.889558    -1.785700     0.616885
H     -0.517766    -1.786248    -1.380627
```

3.6.10 Gal6_1: Gal6-(*g*-)-(5-IMHB)-(*gt*)[c,d]

SCF energy (Hartree) = -844.231648, Dipole Moment (Debye)= 1.7444

C	-0.881584	0.652052	-0.551019
C	0.495109	1.291745	-0.347515
C	1.557363	0.198060	-0.352217
O	1.284468	-0.784723	0.641037
C	0.054501	-1.441933	0.483476
C	-1.103523	-0.428631	0.502975
O	0.563209	2.004610	0.866467
C	0.381894	3.396281	0.753073
C	2.952509	0.712110	-0.066279
O	3.917739	-0.313944	-0.167350
O	-0.017675	-2.173004	-0.695933
C	0.888854	-3.250979	-0.759675
O	-2.351762	-1.024446	0.275814
C	-2.800818	-1.859605	1.311987
O	-1.864342	1.656758	-0.481533
C	-2.925568	1.517030	-1.397458
H	0.655840	-3.811872	-1.663710
H	0.777400	-3.911953	0.108752
H	1.926599	-2.908018	-0.810805
H	-0.003066	-2.113569	1.348504
H	-3.845735	-2.092680	1.108044
H	-2.241251	-2.801189	1.358045
H	-2.734785	-1.360454	2.287088
H	-1.088700	0.050778	1.490377
H	-0.894264	0.180539	-1.541119
H	3.216368	1.469956	-0.806316
H	2.971695	1.176675	0.923905
H	1.554655	-0.277472	-1.340172
H	0.690420	1.965788	-1.190685
H	0.496202	3.813742	1.753634
H	1.142659	3.843493	0.099955
H	-0.610622	3.647348	0.370751
H	-2.562093	1.534649	-2.432972
H	-3.485390	0.593281	-1.232501
H	-3.588581	2.369456	-1.247756
H	3.657138	-1.007294	0.444872

3.6.11 Gal6_2: Gal6-(*g*+)-free-(*tg*)[c,d]

SCF energy (Hartree) = -844.229665, Dipole Moment (Debye)= 1.6411

C	-1.176528	0.515357	0.265869
C	-1.280958	-1.007787	0.232153
O	-0.377925	-1.573606	-0.706213
C	0.971448	-1.267758	-0.486199
C	1.189311	0.254856	-0.493934
C	0.277678	0.912544	0.535651
C	-2.657289	-1.508204	-0.187920
O	-3.695869	-0.959077	0.595537
O	1.450183	-1.775614	0.716422
C	1.489734	-3.184020	0.766658
O	2.514719	0.617965	-0.218911
C	3.438365	0.271856	-1.218700
O	0.384880	2.315793	0.517327
C	1.257189	2.860167	1.480930
O	-1.680703	1.019232	-0.950391
C	-2.236419	2.313782	-0.904746
H	1.952592	-3.457702	1.713782
H	2.092027	-3.590551	-0.055181
H	0.488475	-3.622029	0.717046
H	1.493163	-1.737956	-1.328261
H	4.372403	0.782574	-0.984997
H	3.634116	-0.806329	-1.247915
H	3.097829	0.596598	-2.210194
H	0.900160	0.612535	-1.491012
H	0.546119	0.530861	1.528314
H	-2.800852	-1.294435	-1.249632
H	-2.692450	-2.589958	-0.052253
H	-1.052989	-1.376432	1.239242
H	-1.786061	0.884988	1.100167
H	-2.805183	2.445380	-1.826075
H	-2.918441	2.424881	-0.051147
H	-1.470230	3.087912	-0.836768

```
H      0.936550     2.599970     2.497692
H      2.285786     2.522411     1.334170
H      1.215860     3.943988     1.370250
H     -3.886331    -0.079016     0.264042
```

3.6.12 Gal6_3: Gal6-(*g*-)-(6-ring)-(*gg*)[c,d]

```
SCF energy (Hartree) = -844.229494, Dipole Moment (Debye)= 3.0694

C     -1.113107    -0.619582    -0.500281
C     -1.297667     0.896819    -0.565288
O     -0.563514     1.544808     0.460508
C      0.817323     1.309369     0.425820
C      1.118845    -0.195550     0.530551
C      0.378034    -0.955856    -0.563769
C     -2.746496     1.347744    -0.468273
O     -3.361315     1.099046     0.769905
O      1.416366     1.798993    -0.730539
C      1.387459     3.204956    -0.836256
O      2.485655    -0.488299     0.429128
C      3.264157    -0.041959     1.509970
O      0.539435    -2.348002    -0.438421
C      1.546415    -2.907483    -1.250374
O     -1.711106    -1.115188     0.687516
C     -2.353632    -2.368331     0.559121
H      1.945661     3.470106    -1.733122
H      1.864409     3.674740     0.032598
H      0.365746     3.586520    -0.922821
H      1.200869     1.842275     1.303911
H      4.246225    -0.504182     1.411710
H      3.394408     1.046395     1.505607
H      2.827518    -0.342631     2.470800
H      0.733327    -0.529649     1.502690
H      0.756402    -0.619620    -1.536824
H     -2.786283     2.425848    -0.640541
H     -3.295729     0.863061    -1.288850
H     -0.929325     1.221671    -1.547222
H     -1.608647    -1.064605    -1.370959
H     -2.788756    -2.600148     1.531068
H     -3.158471    -2.321348    -0.184602
H     -1.647912    -3.152689     0.281806
H      1.342753    -2.730009    -2.314085
H      2.533351    -2.508422    -1.005046
H      1.538861    -3.982557    -1.069786
H     -2.986563     0.273620     1.101569
```

3.6.13 Gal6_4: Gal6-(*g*-)-free-(*tg*)[c,d]

```
SCF energy (Hartree) = -844.229466, Dipole Moment (Debye)= 2.6859

C     -1.132193     0.613237     0.211583
C     -1.325061    -0.900463     0.174350
O     -0.449978    -1.503495    -0.765280
C      0.911913    -1.287512    -0.506869
C      1.231839     0.217329    -0.468624
C      0.329519     0.926812     0.535357
C     -2.732388    -1.314907    -0.224187
O     -3.702345    -0.860124     0.698206
O      1.323877    -1.858603     0.692905
C      1.277269    -3.267189     0.700369
O      2.568957     0.479789    -0.137849
C      3.499621     0.116561    -1.124761
O      0.509925     2.322334     0.505040
C      1.377824     2.831922     1.489848
O     -1.503599     1.161994    -1.031685
C     -2.294001     2.324399    -0.951800
H      1.692604    -3.596824     1.651785
H      1.879507    -3.685876    -0.115301
H      0.253617    -3.642481     0.606372
H      1.423765    -1.769493    -1.348115
H      4.458979     0.546469    -0.837062
H      3.619981    -0.970375    -1.202916
H      3.216788     0.512385    -2.108472
H      1.005739     0.614693    -1.466357
H      0.550302     0.540640     1.538016
H     -2.981130    -0.870147    -1.186903
H     -2.760901    -2.404100    -0.333168
```

```
H     -1.111233    -1.291373     1.179159
H     -1.763207     1.020636     1.008147
H     -2.519670     2.626744    -1.974738
H     -3.237266     2.128025    -0.427632
H     -1.764528     3.138492    -0.448623
H      1.008601     2.609862     2.499570
H      2.389992     2.433081     1.385780
H      1.404366     3.914270     1.360901
H     -3.607059    -1.350922     1.517264
```

3.6.14 Glc6_1: Glc6-(*g*+)-(5-IMHB)-(*gg*)[c,d]

```
SCF energy (Hartree) = -844.228749, Dipole Moment (Debye)= 2.0692

C     -1.259722     1.017162    -0.120036
O     -0.307480     1.619360     0.746988
C      1.029709     1.421758     0.358757
C      1.368326    -0.067643     0.399984
C      0.408781    -0.849444    -0.505462
C     -1.052407    -0.496490    -0.206516
O      1.286501     1.909825    -0.911757
C      1.242740     3.316352    -1.006318
O      2.713353    -0.213636     0.031299
O      0.650719    -2.246484    -0.492433
C     -2.621602     1.386999     0.435395
O     -2.807141     0.945093     1.763409
H      1.520163     3.575837    -2.026726
H      1.956262     3.778508    -0.313884
H      0.242692     3.708069    -0.796362
H      1.625890     1.959125     1.105387
H      1.229453    -0.372297     1.445424
H      0.617254    -0.555292    -1.535489
H     -2.743289     2.474371     0.366593
H     -3.395709     0.917736    -0.170959
H     -1.170743     1.433761    -1.129362
C      0.425529    -2.940452     0.710286
H      0.994959    -2.534127     1.552737
H     -0.631649    -2.968628     0.993861
H      0.757669    -3.964571     0.540076
H     -1.351460    -0.925837     0.756962
O     -1.907408    -0.952337    -1.229672
C     -2.514429    -2.203158    -1.015799
H     -3.261629    -2.331466    -1.799306
H     -1.794809    -3.024718    -1.081536
H     -3.020612    -2.240367    -0.043230
C      3.411144    -1.269586     0.644881
H      4.465393    -1.133591     0.402967
H      3.300658    -1.242725     1.736789
H      3.089431    -2.246820     0.274633
H     -2.086679     1.305476     2.287718
```

3.6.15 Glc6_2: Glc6-(*g*+)-free-(*gt*)[c,d]

```
SCF energy (Hartree) = -844.225841, Dipole Moment (Debye)= 2.4332

C     -1.526323     0.138720    -0.120628
O     -1.143459    -0.972271    -0.910458
C     -0.058086    -1.695272    -0.392175
C      1.189168    -0.812409    -0.379409
C      0.921451     0.459167     0.434793
C     -0.380363     1.148282     0.003941
O     -0.294993    -2.167282     0.890144
C     -1.284423    -3.171336     0.951969
O      2.256026    -1.568462     0.126927
O      2.029942     1.342772     0.462524
C     -2.745955     0.762688    -0.772252
O     -3.909532    -0.029351    -0.660374
H     -1.354163    -3.486244     1.992087
H     -1.005568    -4.034448     0.335865
H     -2.259561    -2.802539     0.619629
H      0.086701    -2.530580    -1.087089
H      1.376159    -0.555026    -1.430043
H      0.798458     0.158339     1.476659
H     -2.902338     1.756643    -0.342007
H     -2.554663     0.876944    -1.841294
H     -1.790318    -0.192144     0.891749
C      2.420128     1.944029    -0.747501
```

H	2.616731	1.218311	-1.543116
H	1.686282	2.667986	-1.117611
H	3.347070	2.478726	-0.540229
H	-0.244945	1.625939	-0.976633
O	-0.787066	2.103513	0.956955
C	-0.344996	3.421625	0.743524
H	-0.867481	4.051676	1.463583
H	0.732040	3.524504	0.903683
H	-0.595699	3.768625	-0.267191
C	3.525126	-1.278343	-0.405202
H	4.199806	-2.060933	-0.057654
H	3.513108	-1.293972	-1.502952
H	3.904228	-0.310991	-0.063687
H	-4.187543	-0.035083	0.258154

3.6.16 Glc6_3: Glc6-(*g*+)-(6-IMHB)-(*tg*)[c,d]

SCF energy (Hartree) = -844.225765, Dipole Moment (Debye)= 3.9189

C	-1.322464	0.818032	0.221984
O	-0.381029	1.510271	1.022094
C	0.908131	1.551253	0.459977
C	1.475541	0.135366	0.356845
C	0.524678	-0.766802	-0.443028
C	-0.911166	-0.650734	0.083022
O	0.921228	2.135331	-0.795515
C	0.630426	3.515916	-0.789708
O	2.752826	0.220940	-0.213457
O	0.984483	-2.104835	-0.531033
C	-2.678964	0.991459	0.892078
O	-3.749901	0.632469	0.051330
H	0.743300	3.868994	-1.813457
H	1.329294	4.061037	-0.144272
H	-0.391222	3.715642	-0.452588
H	1.506379	2.140143	1.164766
H	1.546844	-0.227789	1.390220
H	0.528145	-0.417103	-1.477026
H	-2.698267	0.417749	1.829412
H	-2.801255	2.045260	1.148154
H	-1.371451	1.261950	-0.778631
C	1.073062	-2.850588	0.659012
H	1.717078	-2.383693	1.410848
H	0.096066	-3.040813	1.115071
H	1.511720	-3.810373	0.386637
H	-0.987272	-1.111544	1.077335
O	-1.851016	-1.250172	-0.791412
C	-2.079573	-2.633394	-0.618804
H	-2.933405	-2.890821	-1.245376
H	-1.218623	-3.227720	-0.931012
H	-2.328434	-2.865915	0.423366
C	3.698499	-0.719222	0.234361
H	4.667787	-0.396618	-0.146043
H	3.748938	-0.747083	1.330597
H	3.491010	-1.724739	-0.142251
H	-3.478380	-0.166323	-0.416229

[a] Descriptors *g*, *t* refer to the dihedral angle between carbinol C–H and O–H bonds. [b] IMHB descriptors refer to ring size and fused bond stereochemistry. [c] C5–C6 conformational descriptors as conventionally defined. [d] Descriptors *g*, *t* refer to the dihedral angle between C5–C6 and O–H bonds.

4 Synthesis

4.1 General Methods.

All chemical reagents were obtained from commercial sources and used without further purification. Anhydrous solvents were purchased from commercial sources. All glassware was flame-dried under vacuum and cooled under Ar prior to use. Water or air-sensitive reactions were performed under an inert atmosphere, using dry solvents. Reactions were monitored by TLC (Merck Kieselgel 60 F_{254}, aluminium sheet) and spots were visualised by UV and/or by exposure to CAM stain (Hanessian's Stain; Cerium Ammonium Molybdate stain) or Phosphomolybdic Acid (PMA) or Sugar dip stain for carbohydrates, followed by brief heating. Flash column chromatography was performed on silica gel (Merck silica gel 60, particle size 40–63 µm). All reported solvent mixtures are volume measures. Nuclear magnetic resonance spectra were recorded using either a Bruker Ultrashield 400 MHz or 500 MHz spectrometer. The chemical shift (δ) is given in ppm using the residual solvent peak as an internal standard. The coupling constants (*J*) are shown in Hertz (Hz).

4.2 Synthesis of final cyclohexanol compounds

4.2.1 *Cis*-4-*tert*-butyl-*trans*-2-methoxy cyclohexanol (**10b**)

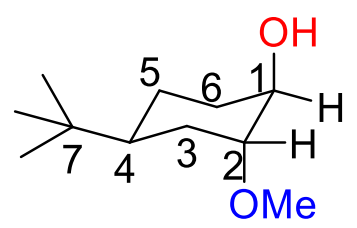

10b

Prepared according to the literature procedure, reported by Rosenberg *et. al.*[21] **^{1}H NMR** (400 MHz, $CDCl_3$) δ 3.90 (dd, 1H, *J*=5.7, 2.8 Hz, H-2), 3.39-3.43 (m, 1H, H-1), 3.35 (s, 3H, OC*H*$_3$), 1.63-1.91 (m, 3H, O*H*, H-$3_{ax/eq}$ & H-$6_{ax/eq}$), 1.47-1.60 (m, 2H, H-$3_{ax/eq}$ & H-$5_{ax/eq}$), 1.29-1.43 (m, 3H, H-4, H-$5_{ax/eq}$ & H-$6_{ax/eq}$), 0.86 (s, 9H, H-*t*-Bu) ppm; **^{13}C NMR** (101 MHz, $CDCl_3$) δ 79.6 (C-2), 66.9 (C-1), 56.4 (O*C*H$_3$), 40.6 (C-4), 32.1 (C-7), 28.7 (C-6), 27.3 (*t*-Bu), 25.1 (C-3), 20.3 (C-5) ppm.

The ^{1}H and ^{13}C NMR spectra corresponded to the reported data.[21]

4.2.2 *Cis*-4-*tert*-butyl-*cis*-2-methoxy cyclohexanol (**10c**)

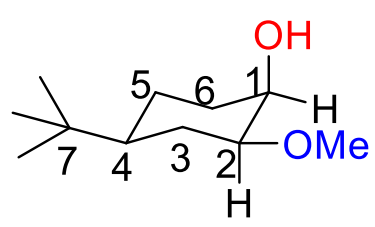

10c

Prepared according to the literature procedure, reported by Rosenberg *et. al.*[21] **^{1}H NMR** (400 MHz, $CDCl_3$) δ 4.10 (br. s., 1H, H-1), 3.40 (s, 3H, OC*H*$_3$), 3.15 (ddd, 1H, *J*=11.6, 4.6, 2.8 Hz, H-2), 2.09 (s, 1H,O*H*), 2.00-2.06 (m, 1H, H-$6_{ax/eq}$), 1.75-1.82 (m, 1H, H-$3_{ax/eq}$), 1.42-1.48 (m, 1H, H-$5_{ax/eq}$), 1.29-1.37 (m, 3H, H-$3_{ax/eq}$, H-$5_{ax/eq}$, H-$6_{ax/eq}$), 0.98-1.07 (m, 1H, H-4), 0.87 (s, 9H, *t*-Bu) ppm; **^{13}C NMR** (101 MHz, $CDCl_3$) δ 81.8 (C-2), 65.2 (C-1), 55.8 (O*C*H$_3$), 46.4 (C-4), 32.4 (C-7), 30.1 (C-6), 27.5 (*t*-Bu), 26.7 (C-3), 20.0 (C-5) ppm.

The ^1H and ^{13}C NMR spectra corresponded to the reported data.[21]

4.2.3 *Trans*-4-*tert*-butyl-*trans*-2-methoxy cyclohexanol (**11b**)

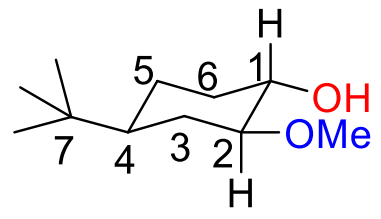

11b

Prepared according to the literature procedure, reported by Rosenberg et. al.[21] **^{1}H NMR** (400 MHz, $CDCl_3$) δ 3.42 (s, 3H, OC*H*$_3$), 3.37 (ddd, 1H, J=11.3, 8.8, 4.8 Hz, H-1), 2.96 (ddd, 1H, J=11.1, 8.8, 4.3 Hz, H-2), 2.66 (s, 1H,O*H*), 2.12-2.19 (m, 1H, H-$3_{ax/eq}$), 1.99-2.07 (m, 1H, H-$6_{ax/eq}$), 1.71-1.78 (m, 1H, H-$5_{ax/eq}$), 1.21-1.34 (m, 2H, H-$6_{ax/eq}$ & H-$3_{ax/eq}$), 1.03-1.15 (m, 2H, H-$5_{ax/eq}$ & H-4), 0.88 (s, 9H, *t*-Bu) ppm; **^{13}C NMR** (101 MHz, $CDCl_3$) δ 85.1 (C-2), 73.7 (C-1), 56.2 (O*C*H$_3$), 45.9 (C-4), 32.1 (C-7), 31.2 (C-6), 29.1 (C-3), 27.3 (*t*-Bu), 24.6 (C-5) ppm.

The ^1H and ^{13}C NMR spectra corresponded to the reported data.[21]

4.3 Synthesis of final carbohydrate compounds

4.3.1 Synthesis of methyl 3,4,6-tri-*O*-methyl-α-D-glucopyranoside (**Glc2**)

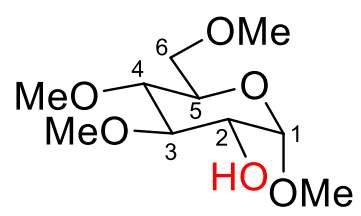

Glc2

Prepared according to the literature procedure, reported by Thiem *et. al.*[22] **^{1}H NMR** (400 MHz, $CDCl_3$) δ 4.75 (d, 1H, *J* = 3.9 Hz, H-1), 3.61 – 3.53 (m, 4H, H-5, H-6, H-2), 3.35 (t, 1H, *J* = 9.2 Hz, H-3), 3.19 (t, 1H, *J* = 9.2 Hz, H-4), 2.07 (d, 1H, *J* = 8.0 Hz, O*H*), 3.65, 3.52, 3.42, 3.41 (OC*H*$_3$) ppm; **^{13}C NMR** (101 MHz, $CDCl_3$) δ 99.5 (C-1), 84.6 (C-3), 79.4 (C-4), 72.5 (C-2), 71.1 (C-6), 70.3 (C-5), 61.0, 60.4, 59.3, 55.4 (O*C*H3) ppm.

The NMR data were consistent with those reported in the literature.[22]

4.3.2 Synthesis of methyl 2,4,6-tri-*O*-methyl-α-D-glucopyranoside (**Glc3**)

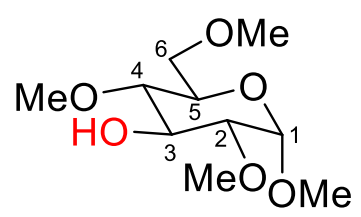

Glc3

Prepared according to the literature procedure, reported by Thiem *et. al.*[22] **^{1}H NMR** (400 MHz, $CDCl_3$) δ 4.87 (d, 1H, *J* = 3.5 Hz, H-1), 3.90 (app dt, 1H, *J* = 9.3, 2.03 Hz, H-3), 3.64 – 3.56 (m, 3H, H-5, H-6), 3.29 – 3.22 (m, 1H, H-4), 3.23 (dd, 1H, *J* = 9.6, 3.5 Hz, H-2), 2.43 (d, 1H, *J* = 2.1 Hz, O*H*), 3.55, 3.48, 3.41, 3.40 (OC*H*$_3$) ppm; **^{13}C NMR** (101 MHz, CDCl3) δ 96.9 (C-1), 81.4 (C-2), 79.2 (C-4), 73.2 (C-3), 71.2 (C-6), 69.7 (C-5), 60.6, 59.3, 58.5, 55.3 (O*C*H$_3$) ppm.

The NMR data were consistent with those reported in the literature.[22]

4.3.3 Synthesis of methyl 2,3,6-tri-*O*-methyl-α-D- glucopyranoside (**Glc4**)

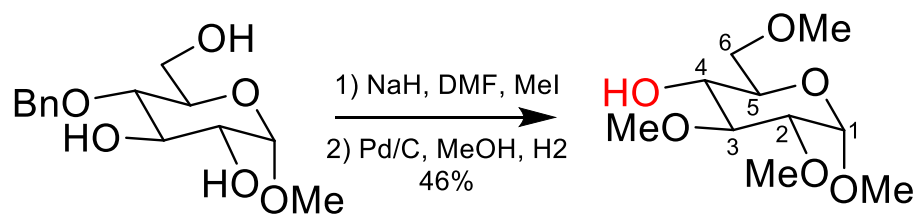

Glc4

To a 500 mL two-neck round-bottom flask were added methyl 4-*O*-benzyl-α-D-glucopyranoside (prepared following literature procedures,[23] 3.90 g, 13.72 mmol, 1.0 equiv and anhydrous DMF (140 mL, 10.2 mL/mmol). The resulting mixture was cooled to 0 °C. At this temperature, NaH (60wt% dispersion in mineral oil, 5.50 g, 137.5 mmol, 10.0 equiv) was added and stirred for 1 h, followed by the addition of MeI (8.6 mL, 138.1 mmol, 10.1 equiv) at 0 °C. The resulting reaction mixture was stirred at room temperature overnight. On the following day, the reaction was slowly quenched with MeOH (20 mL), and the solvent was removed by evaporation. The residue was diluted with water (150 mL) and extracted with CH_2Cl_2 (150 mL × 3). The combined organic layer was dried over Na_2SO_4, filtered and concentrated. The crude mixture was purified by flash chromatography to afford 2.57 g of intermediate, which was used directly for further hydrogenolysis reaction. To a 500 mL two-neck round-bottom flask, this intermediate (2.57 g), methanol (137 mL), and Pd/C (10 wt% loading, 603 mg) were added under argon. The resulting reaction mixture was degassed with H_2 gas and stirred under H_2 at room temperature for 24 h. Upon completion, as indicated by TLC analysis, the reaction mixture was filtered over a pad of silica gel and concentrated. The crude mixture was purified by flash chromatography (eluent: acetone/petroleum ether, 15/85 → 28/72) to afford the desired product, **Glc4** (1.50 g, 6.35 mmol, 46% yield over two steps), as a gummy solid. Yield the desired product **Glc4** (1.50 g, 6.35 mmol, 46% over two steps) as a gummy solid, purified by flash chromatography (eluent: acetone/petroleum ether 15/85 → 28/72).

^{1}H NMR (400 MHz, $CDCl_3$) δ 4.84 (d, 1H, *J* = 3.5 Hz, H-1), 3.71(1H, d, *J* = 9.2, 4.1, 3.6 Hz, H-5), 3.58 – 3.64 (m, 5H, H-6, with a OC*H*3 singlet at 3.62 ppm), 3.50 (td, 1H, *J* = 9.0, 2.6 Hz, H-4), 3.44 (1H, t, *J* 9.2 Hz, H-3), 3.23 (dd, 1H, *J* = 9.3, 3.5 Hz, H-2), 2.79 (d, 1H, *J* = 1.1 Hz, -O*H*), 3.62, 3.48, 3.43, 3.40 (s, 3H, OC*H*3), ppm; **^{13}C NMR** (101 MHz, $CDCl_3$) δ 97.5 (C-1), 82.8(C-3), 81.7(C-2), 71.9(C-6), 70.5(C-4), 69.7(C-5), 61.1, 59.4, 58.4, 55.2 (O*C*H3) ppm.

The NMR data were consistent with those reported in the literature.[22]

4.3.4 Synthesis of methyl-2,3,4-tri-*O*-methyl-α-D-glucopyranosid (**Glc6**)

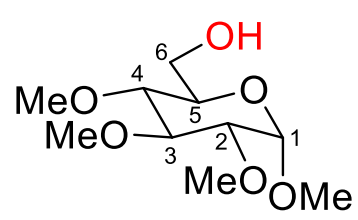

Glc6

Prepared according to the literature procedure, reported by Thiem *et. al.*[22] **^{1}H NMR** (400 MHz, $CDCl_3$) δ 4.81 (d, 1H, *J* = 3.6 Hz, H-1), 3.83 (ddd, 1H, *J* = 11.8, 5.1, 2.9 Hz, H-6), 3.75 (ddd, 1H, *J* = 11.7, 7.8, 4.2 Hz, H-6'), 3.58 – 3.46 (m, 2H, H-3, H-5), 3.19 – 3.09 (m, 2H, H-2, H-4), 1.83 (dd,

1H, J = 7.8, 5.1 Hz, H-1), 3.61, 3.55, 3.52, 3.39 (OC$\underline{H}_3$) ppm; **^{13}C NMR** (101 MHz, $CDCl_3$) δ 97.6 (C-1), 83.5 (C-3), 82.0 (C-2), 79.7 (C-4), 70.7 (C-5), 62.0 (C-6), 61.0, 60.7, 59.2, 55.3 (O$\underline{C}H_3$) ppm.

The NMR data were consistent with those reported in the literature.[22]

4.3.5 Synthesis of methyl 3,4,6-tri-*O*-methyl-α-D-galactopyranoside (**Gal2**)

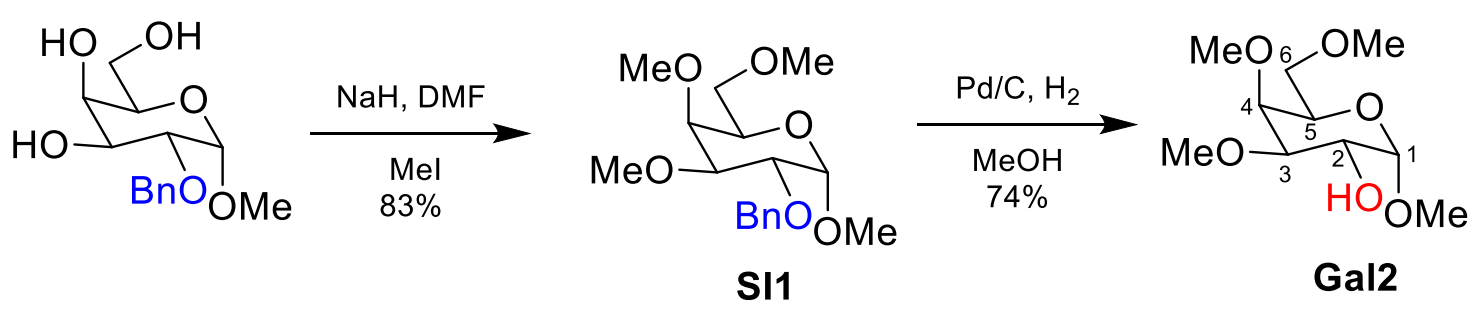

Gal2 was prepared from methyl 2-*O*-benzyl-α-D-galactopyranoside[24] as described for **Glc4**. 2.75 g methyl 2-*O*-benzyl-α-D-galactopyranoside (prepared following literature procedures,[24] 9.67 mmol, 1.0 equiv), 2.09 g (87.05 mmol, 3.0 equiv per -OH) NaH, 2.4 mL (37.72 mmol, 1.3 equiv per -OH) MeI, anhydrous 30.0 mL *N, N*-dimethylformamide. Yield: 2.62 g **SI1** (8.03 mmol, 83%), colourless oil, purified by flash chromatography (n-Hexane/EtOAc; 90:10 → 70:30). **^{1}H NMR** (400 MHz, $CDCl_3$, COSY-HSQC): δ 7.23-7.38 (m, 5H, H_{Ar}), 4.84 (d, J=12.1 Hz, 1H, PhC$\underline{H}_2$), 4.62 (d, J=5.3 Hz, 1H, PhC$\underline{H}_2$), 4.60 (d, J=3.0 Hz, 1H, H-1), 3.86 (app. t, J=6.4 Hz, 1H, H-5), 3.83 (app. dd, J=10.1, 3.4 Hz, 1H, H-2), 3.69 (app. dd, J=3.1, 1.0 Hz, 1H, H-4), 3.62 (dd, J=10.1, 3.0 Hz, 1H, H-3), 3.46-3.55 (m, 2H, H-6),3.57, 3.56, 3.39 , 3.35 (s, 3H, OC$\underline{H}_3$) ppm; **^{13}C NMR** (101 MHz, $CDCl_3$, HSQC) δ 138.7 (C_{Ar}), 128.3, 127.9, 127.6 (CH_{Ar}), 98.7 (C-1), 80.4 (C-3), 76.5 (C-4), 76.3 (C-2), 73.6 (Ph$\underline{C}H_2$), 71.2 (C-6), 68.8 (C-5), 61.3, 59.2, 58.5, 55.3 (O$\underline{C}H_3$) ppm;

This was used for further hydrogenolysis reaction. 2.48 g **SI1** (2.48 mmol), Pd/C (300 mg), 50.0 mL MeOH. Yield: 1.32 g of **Gal2** (5.59 mmol, 74%), white solid needles, purified by re-crystallisation (n-Hexane/Et_2O; 90:10). **^{1}H NMR** (400 MHz, C_6D_6) δ 4.70 (d, J=3.9 Hz, 1H, H-1), 4.29 (app. ddd, J=10.0, 6.9, 3.9 Hz, 1H, H-2), 3.87 (app. dt, J=6.7, 0.6 Hz, 1H, H-5), 3.62 (dd, J=9.2, 7.0 Hz, 1H, H-6), 3.53 (dd, J=9.2, 5.9 Hz, 1H, H-6'), 3.54-3.53 (m, 1H, H-4), 3.33 (dd, J=10.0, 2.9 Hz, 1H, H-3), 2.07 (br d, J=7.2 Hz, 1H, O$\underline{H}$), 3.45, 3.26, 3.13, 3.10 (s, 3H, OC$\underline{H}_3$) ppm; **^{13}C NMR** (101 MHz, C_6D_6, HSQC) δ 100.8 (C-1), 82.4 (C-3), 76.7 (C-4), 72.1 (C-6), 70.4 (C-5), 70.0 (C-2), 61.2, 59.2, 58.4, 55.4 (O$\underline{C}H_3$) ppm.

^{1}H NMR in C_6D_6 data were consistent with the data reported in the literature.[25]

^{1}H NMR (400 MHz, $CDCl_3$): δ 4.82 (d, J=3.9 Hz, 1H, H-1), 3.98 (ddd, J=10.2, 6.6, 3.9 Hz, 1H, H-2), 3.85 (app. t, J=6.6 Hz, 1H, H-5), 3.72 (d, J=2.5 Hz, 1H, H-4), 3.48 - 3.59 (m, 8H, H-6, OC$\underline{H}_3$), 3.39 - 3.43 (m, 7H, H-3, OC$\underline{H}_3$), 2.24 (d, J=6.9 Hz, 1H, -O$\underline{H}$) ppm; **^{13}C NMR** (101 MHz, $CDCl_3$, HSQC) δ 99.5 (C-1) 81.1 (C-3), 75.0 (C-4), 71.1 (C-6), 69.2 (C-5), 68.5 (C-2), 61.1, 59.2, 57.6, 55.4 (O$\underline{C}H_3$) ppm.

The NMR data were consistent with those reported in the literature.[26]

4.3.6 Synthesis of methyl 2,4,6-tri-*O*-methyl-α-D-galactopyranoside (**Gal3**)

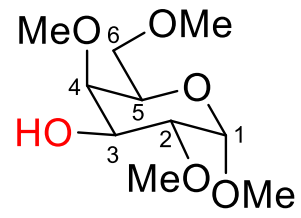

Gal3

Prepared according to the literature procedure, reported by Thiem *et. al.*[27] **^{1}H NMR** (400 MHz, $CDCl_3$) δ 4.86 (d, *J*=3.6 Hz, 1H, H-1), 3.90 (ddd, *J*=10.1, 4.5, 3.5 Hz, 1H, H-3), 3.85 (br td, *J*=6.5, 0.8 Hz, 1H, H-5), 3.55 (dd, *J*=3.4, 1.1 Hz, 1H, H-4), 3.51 (dd, *J*=9.4, 6.3 Hz, 1H, H-6), 3.48 (dd, *J*=9.6, 6.6 Hz, 1H, H-6'), 3.44 (dd, *J*=10.1, 3.6 Hz, 1H, H-2), 2.59 (br d, *J*=5.3 Hz, 1H, O*H*),3.53, 3.44, 3.36, 3.35 (s, 3H, $OC\underline{H}_3$) ppm; **^{13}C NMR** (101 MHz, $CDCl_3$) δ 97.0 (C-1), 78.9 (C-4), 78.7 (C-2), 71.1 (C-6), 69.9 (C-3), 68.7 (C-5), 61.6, 59.0, 58.1, 55.2 ($O\underline{C}H_3$) ppm.

The NMR data were consistent with those reported in the literature.[27]

4.3.7 Synthesis of methyl 2,3,6-tri-*O*-methyl-α-D-galactopyranoside (**Gal4**)

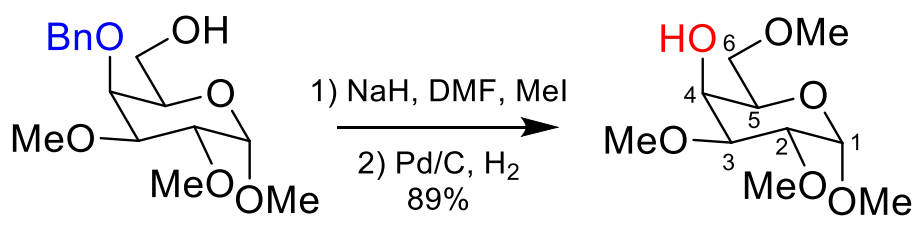

Gal4

Gal4 was prepared from methyl 4-*O*-benzyl-2,3-di-*O*-methyl-α-D-galactopyranoside[24] as described for **Glc4**. 2.05 g methyl 4-*O*-benzyl-2,3-di-*O*-methyl-α-D-galactopyranoside (prepared following literature procedures,[28] 6.56 mmol, 1.0 equiv), 0.47 g (19.69 mmol, 3.0 equiv) NaH, 0.5 mL (8.53mmol, 1.3 equiv) MeI, 20.0 mL *N, N*-dimethylformamide. Yield: 2.04 g (6.25 mmol, 94%), colourless oil, purified by flash chromatography (n-Hexane/acetone; 80:20). This 2.04 g intermediate was used directly for further hydrogenolysis reaction. The intermediate (2.04 g), methanol (100 mL), and Pd/C (10wt% loading, 823 mg). Yield the desired product **Gal4** (1.38 g, 5.83 mmol, 89% over two steps) as viscous oil, purified by flash chromatography (eluent *n*-hexane/acetone; 80:20 → 50:50). **^{1}H NMR** (400 MHz, $CDCl_3$) δ 4.90 (d, *J*=3.5 Hz, 1H, H-1), 4.11 (dt, *J*=3.1, 1.5 Hz, 1H, H-4), 3.86 (app t, *J*=5.5 Hz, 1H, H-5), 3.66 (dd, *J*=10.2, 5.2 Hz, 1H, H-6), 3.65 (dd, *J*=10.1, 6.1 Hz, 1H, H-6'), 3.59 (dd, *J*=9.9, 3.6 Hz, 1H, H-2), 3.53 (dd, *J*=9.9, 3.2 Hz, 1H, H-3), 2.68 (t, *J*=1.4 Hz, 1H, O*H*), 3.50, 3.49, 3.43, 3.41 (s, 3H, $OC\underline{H}_3$) ppm; **^{13}C NMR** (101 MHz, $CDCl_3$) δ 97.8 (C-1), 79.1 (C-3), 77.2 (C-2), 72.3 (C-6), 68.1 (C-5), 67.0 (C-4), 59.4, 58.9, 57.6, 55.3 ($O\underline{C}H_3$) ppm.

NMR data were consistent with the data reported in the literature.[27]

4.3.8 Synthesis of methyl 2,3,4-tri-*O*-methyl-α-D-galactopyranoside (**Gal6**)

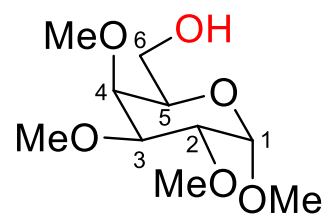

Gal6

Prepared according to the literature procedure, reported by Arnold *et. al.*[26] **^{1}H NMR** (400 MHz, $CDCl_3$, COSY-HSQC-TSE-PSYCHEDELIC) δ 4.93 (d, *J*=3.7 Hz, 1H, H-1), 3.91 (ddd, *J*= 10.9, 6.5, 2.5 Hz, 1H, H-6), 3.80 (app td, *J*= 5.6, 0.7 Hz, 1H, H-5), 3.76 (ddd, *J*=10.8, 8.7, 4.7 Hz, 1H, H-6'), 3.71 (dd, *J*=2.8, 0.9 Hz, 1H, H-4), 3.67 (dd, *J*=10.1, 3.7 Hz, 1H, H-2), 3.57 (dd, *J*=10.2, 3.0 Hz, 1H, H-3), 2.09 (dd, *J*=8.8, 2.8 Hz, 1H, O*H*), 3.59, 3.54, 3.53, 3.44 (s, 3H, OCH_3) ppm; **^{13}C NMR** (101 MHz, $CDCl_3$, HSQC) δ 97.9 (C-1), 80.4 (C-3), 77.8 (C-2), 77.1 (C-4), 70.3 (C-5) 62.3 (C-6), 61.2, 58.9, 58.3, 55.3 (O*C*H_3) ppm.

The NMR data were consistent with those reported in the literature.[26]

5 Copies of spectra

5.1 General

NMR samples were prepared in $CDCl_3$ (Eurisotop) at a concentration of approximately 30 mg/mL for the acquisition of ^1H, ^{13}C, COSY, and HSQC spectra. In selected cases, $CDCl_3$ was dried by storage over freshly activated 4 Å molecular sieves for several days and subsequently filtered through a small Celite pad in a pipette prior to use. This procedure enabled the acquisition of ^1H NMR spectra at very low sample concentrations (~1 mg/mL) to allow observation of hydroxyl (OH) resonances and their multiplicities.

For **Gal6**, carbon tetrachloride (CCl_4, spectroscopic grade) was used as the solvent; it was similarly dried over freshly activated 4 Å molecular sieves for several days prior to use. A sealed capillary containing 0.1 mL of acetone-d_6 was placed in the NMR tube to provide a lock signal. All NMR tubes were sealed with caps and Parafilm to prevent solvent and sample evaporation.

NMR spectra were recorded on Bruker Ultrashield 400 or 500 MHz spectrometers. Chemical shifts (δ) are reported in parts per million (ppm) relative to the residual solvent signal as an internal standard, and coupling constants (J) are reported in hertz (Hz).

The J-coupling measurements on **Gal6** were performed on a Bruker Avance III HD spectrometer operating at a ^1H and ^{13}C frequencies of 600.13 MHz and 150.90 MHz, respectively, equipped with a CP-QCI-F cryoprobe and running Topspin 3.6.2. All experiments were performed at 298.0 K.

For the **Gal6** in CCl_4, the $^3J_{H5\text{-}H6}$, $^3J_{H5\text{-}H6'}$, $^3J_{H6\text{-}OH}$ and $^3J_{H6'\text{-}OH}$ couplings were measured on the H6 and H6' signals using the TSE-PSYCHEDELIC experiment,[29],[30] with the 180° shaped (ISnob2) inversion pulses set to either the H5 or H(O) protons. It applied a 30 ms PSYCHE saltire-shaped pulse and 40 ms 180° chirp pulses, each sweeping 10 kHz, applied during magnetic field gradients of 0.8025 G/cm. The PSYCHE flip angle was set to 15°.

For the **Gal6** in $CDCl_3$, the H5 and H6' protons were significantly strongly coupled and had partly overlapped multiplets. This complicates PSYCHEDELIC measurements and reduces the accuracy of *J*-couplings measurements. To counter this, SERFBIRD and SATASERF experiments were used, which avoid the strong coupling situation by focusing on ^{13}C satellite signals.[31] The $^3J_{H6H(O)}$ and $^3J_{H6'H(O)}$ couplings were measured using SATASERF by setting the 180° shaped (RSnob) refocussing pulse on the H6 $^{13}C^{\beta}$ or H6 $^{13}C^{\beta}$ satellite signals, respectively, with the 180° shaped inversion pulses (ISnob2) set to the H(O) signal. The $^3J_{H5H6}$, $^3J_{H5H6'}$couplings were measured on the H5 $^{13}C^{\alpha}$ satellite signal using SERFBIRD, with the 180° shaped (ISnob2) inversion pulses set to either the H6 or H6' proton

All experiments used a spectral window of 5000 Hz in the direct dimension and 20 Hz or 10 Hz in the J-resolved dimension. All experiments using interferogram pure shift acquisition (PSYCHEDELIC, SATASERF) had a time domain data chunk duration of 12.8 ms. The SERFBIRD experiments employed

a real-time BIRD homodecoupling, with a chunk duration of 40 ms. Interferogram pure shift experiments were processed using the pshift au-program available from the University of Manchester NMR methodology group. All selective 2DJ data were processed as described in the literature.[30-31] Time-domain data was weighted by a Lorentz-to-Gauss resolution enhancement window function and zero-filled prior to the Fourier transform.

5.2 *Cis*-4-*tert*-butyl-*trans*-2-methoxy cyclohexanol (10b)

^{1}H NMR (400 MHz, $CDCl_3$)

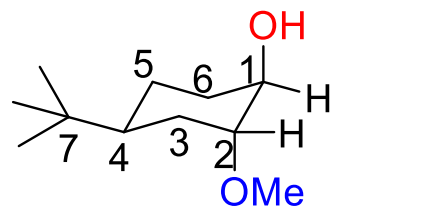

10b

Nucleus	δ (ppm)	m	*J* (Hz)
**OH**	1.63-1.91	m	
H1	3.39-3.43	m	
H2	3.90	dd	$^3J_{H2\text{-}H1}$ = 2.9, $^3J_{H2\text{-}H3}$ = 5.7
$H3_{ax/eq}$	1.63-1.91 and 1.47-1.60	m	
H4	1.29-1.43	m	
$H5_{ax/eq}$	1.47-1.60 and 1.29-1.43	m	
$H6_{ax/eq}$	1.63-1.91 and 1.29-1.43	m	
OCH_3	3.35	s	
t-Bu	0.86	s	

1H NMR, CDCl3, 400 MHz

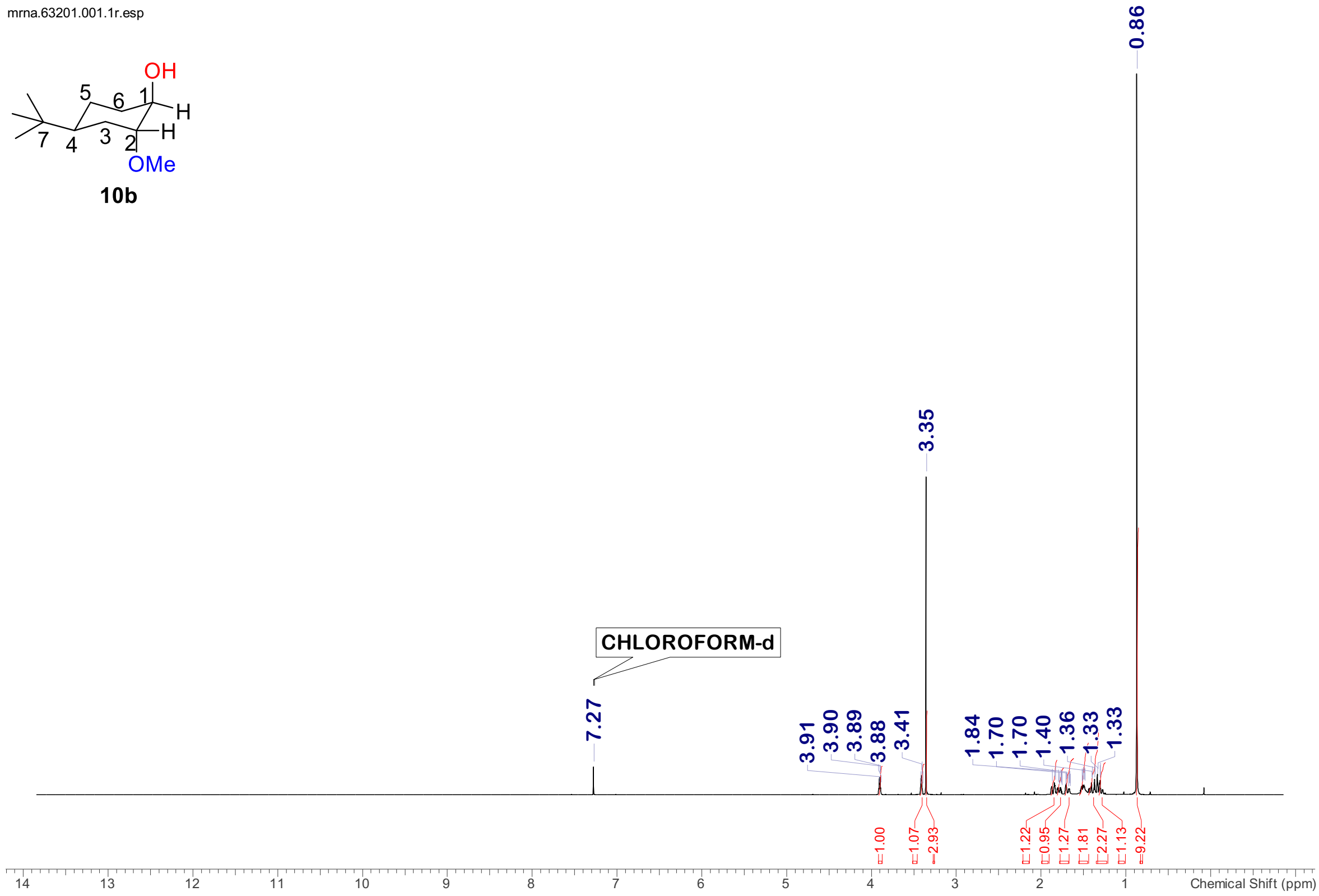

$^{13}C\{^1H\}$ NMR, $CDCl_3$, 101 MHz

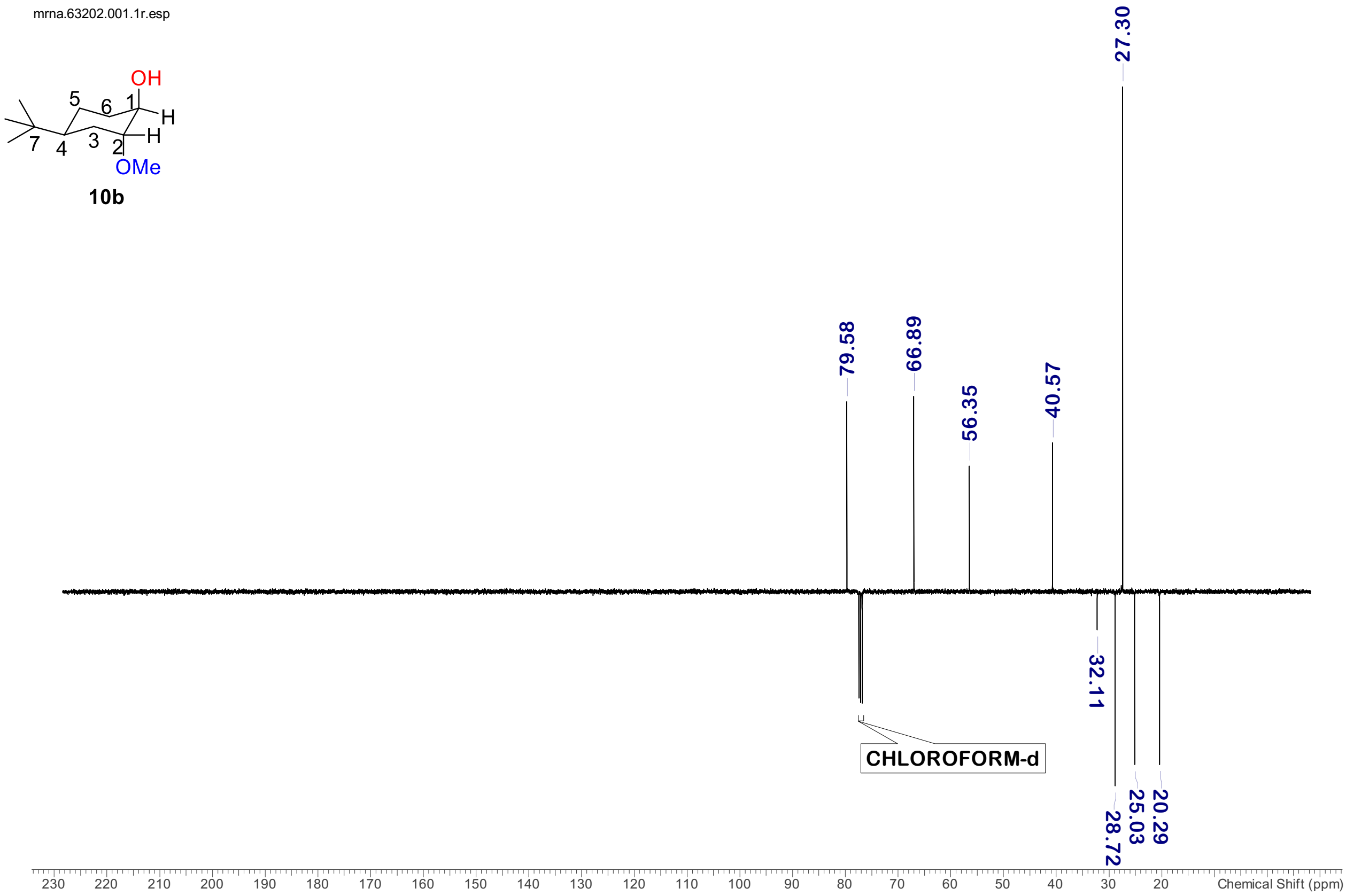

5.3 *Cis*-4-*tert*-butyl-*cis*-2-methoxy cyclohexanol (10c)

^{1}H NMR (400 MHz, $CDCl_3$)

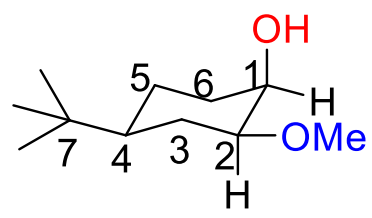

10c

Nucleus	δ (ppm)	m	*J* (Hz)
**OH**	2.09	s	
H1	4.07-4.11	br s	
H2	3.15	ddd	$^3J_{H2\text{-}H1}$ = 2.8, $^3J_{H2\text{-}H3ax}$ = 11.6, $^3J_{H2\text{-}H3eq}$ = 4.6
$H3_{ax/eq}$	1.75-1.82 and 1.29-1.37	m	
H4	0.98-1.07	m	
$H5_{ax/eq}$	1.42-1.48 and 1.29-1.37	m	
$H6_{ax/eq}$	2.00-2.06 and 1.29-1.37	m	
OCH_3	3.40	s	
t-Bu	0.87	s	

1H NMR, CDCl3, 400 MHz

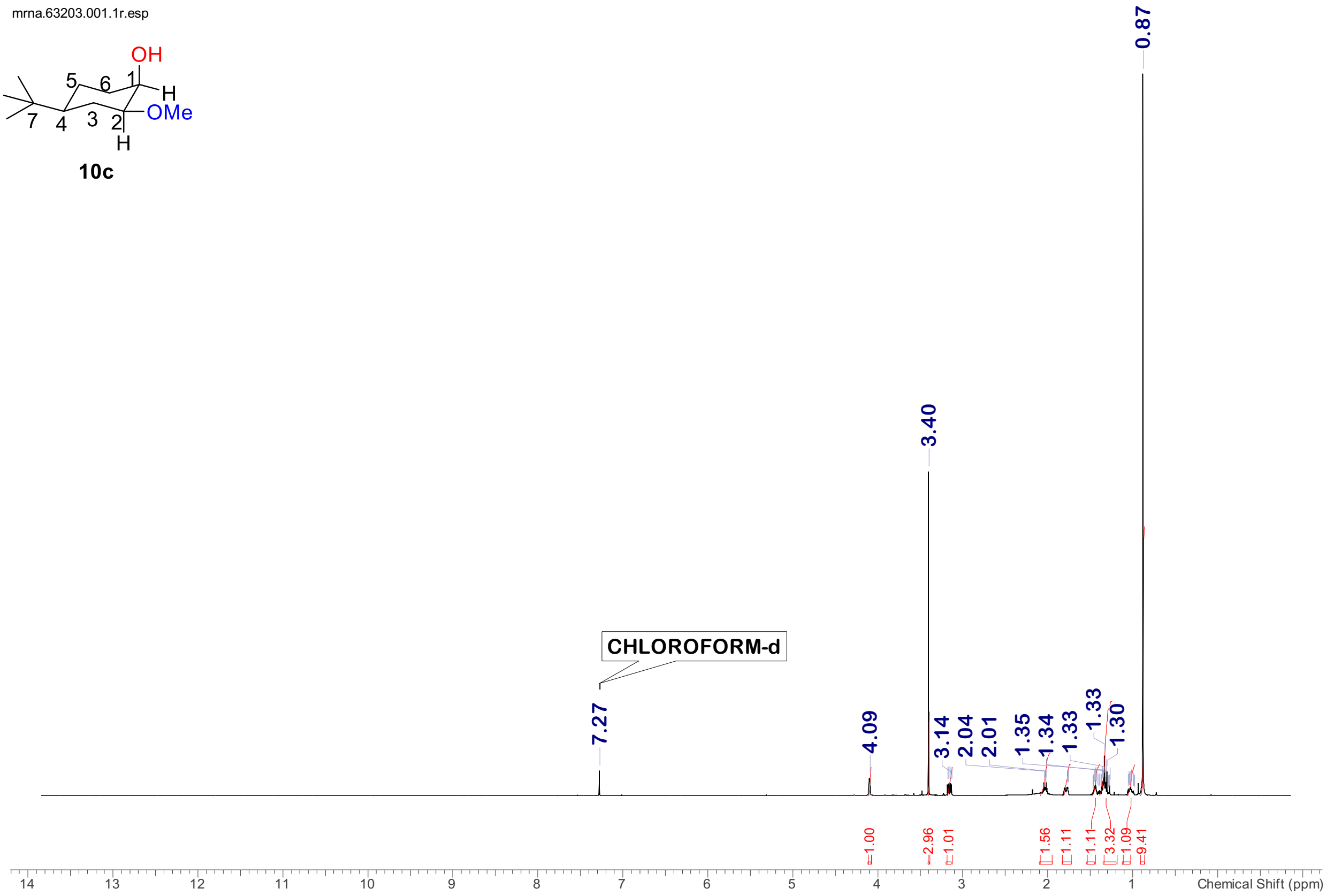

$^{13}C\{^{1}H\}$ NMR, $CDCl_3$, 101 MHz

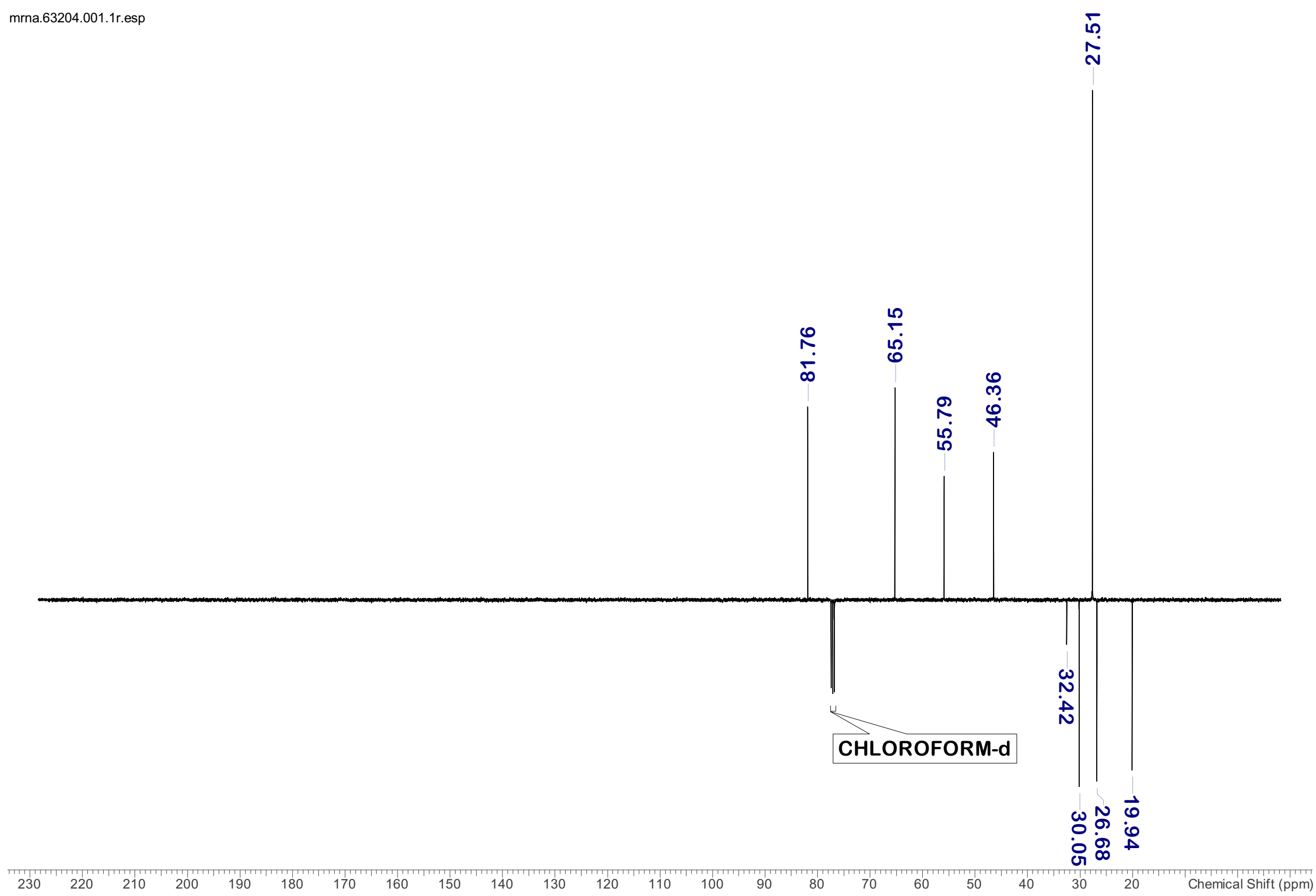

5.4 *Trans*-4-*tert*-butyl-*trans*-2-methoxy cyclohexanol (11b)

^{1}**H NMR** (400 MHz, $CDCl_3$))

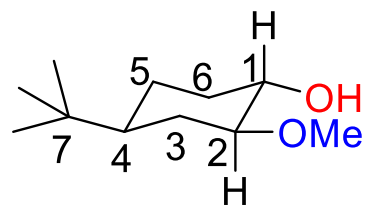

11b

Nucleus	δ (ppm)	m	*J* (Hz)
**OH**	2.73	s	
H1	3.37	ddd	$^{3}J_{H1\text{-}H2}$ = 11.3, $^{3}J_{H2\text{-}H5ax}$ = 4.8, $^{3}J_{H2\text{-}H5eq}$ = 8.8
H2	2.96	ddd	$^{3}J_{H2\text{-}H1}$ = 11.1, $^{3}J_{H2\text{-}H3ax}$ = 4.3, $^{3}J_{H2\text{-}H3eq}$ = 8.8
$H3_{ax/eq}$	2.12-2.19 and 1.21-1.34	m	
H4	1.03-1.15	m	
$H5_{ax/eq}$	1.71-1.78 and 1.03-1.15	m	
$H6_{ax/eq}$	1.99-2.07 and 1.21-1.34	m	
OCH_3	3.42	s	
t-Bu	0.88	s	

1H NMR, CDCl3, 400 MHz

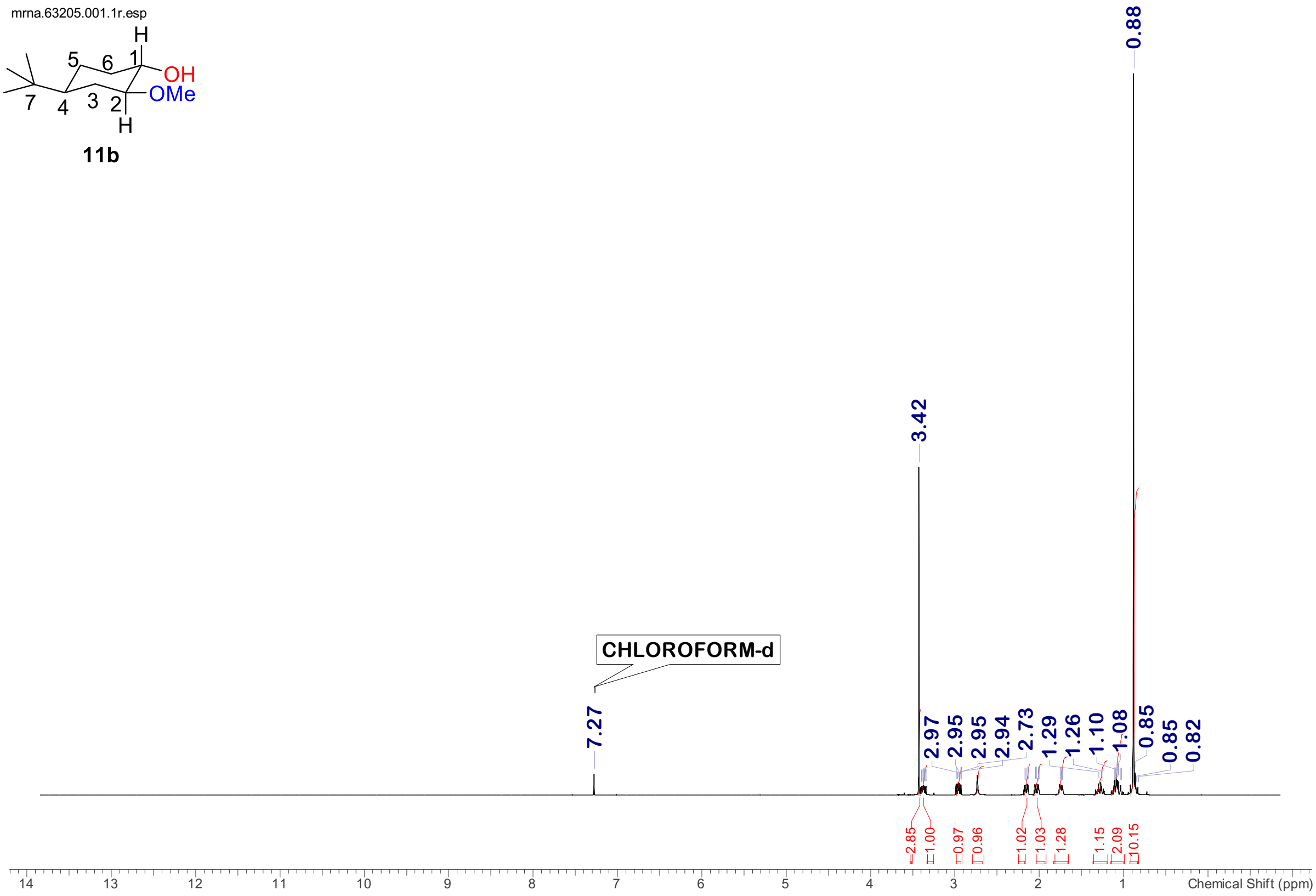

$^{13}C\{^{1}H\}$ NMR, $CDCl_3$, 101 MHz

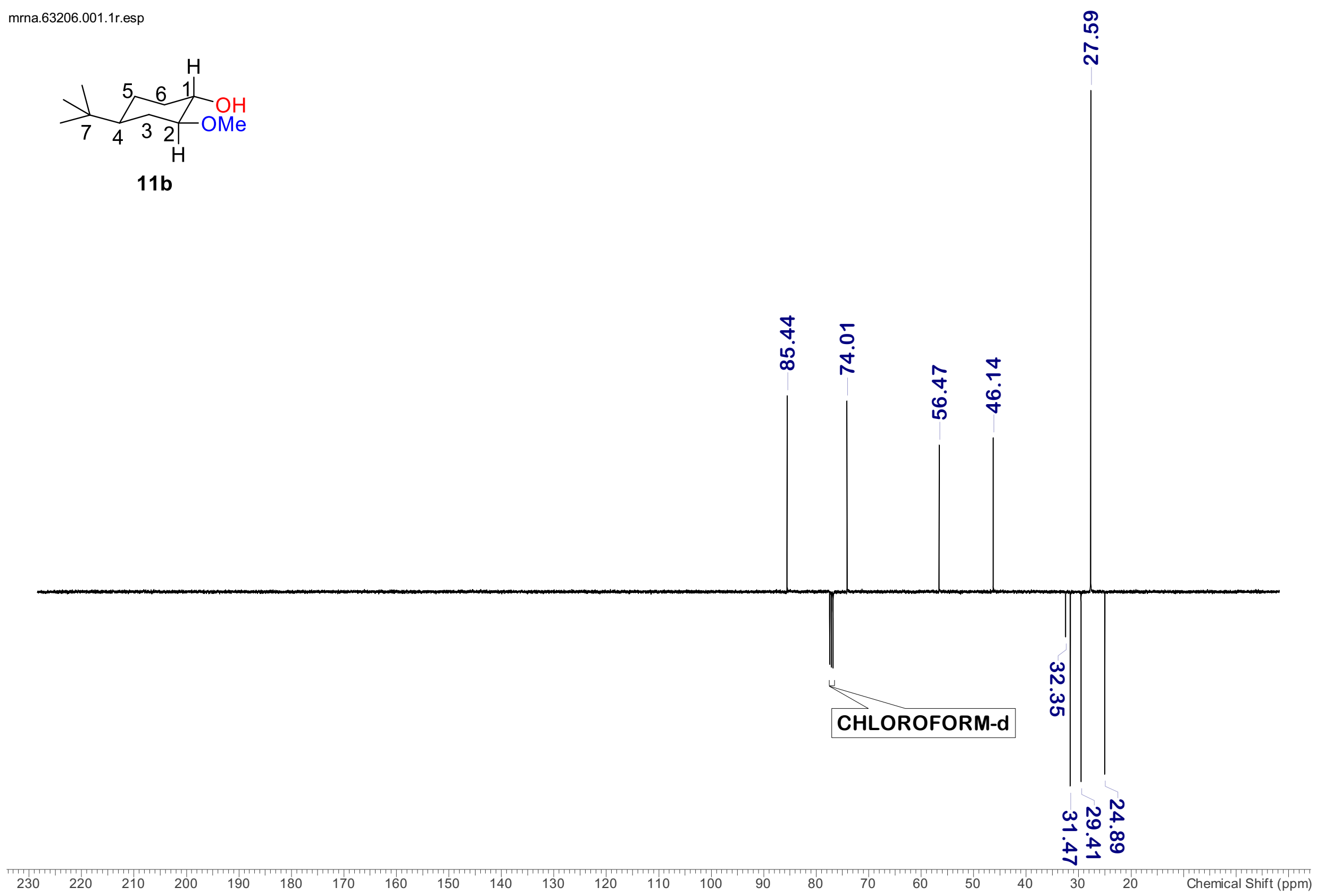

5.5 Methyl 3,4,6-tri-*O*-methyl-α-D-glucopyranoside (Glc2), in $CDCl_3$.

^{1}H NMR (400 MHz, $CDCl_3$))

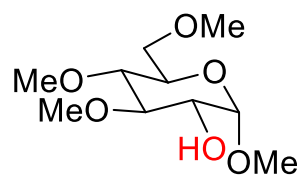

Glc2

Nucleus	δ (ppm)	m	*J* (Hz)
**OH**	2.07	d	$^{3}J_{OH\text{-}H2} = 8.0$
H1	4.75	d	$^{3}J_{H1\text{-}H2} = 3.9$
H2	3.61 – 3.53	m	
H3	3.35	t	$^{3}J_{H3\text{-}H2} = {}^{3}J_{H3\text{-}H4} = 9.2$
H4	3.19	t	$^{3}J_{H4\text{-}H3} = {}^{3}J_{H4\text{-}H5} = 9.2$
H5	3.61 – 3.53	m	
H6	3.61 – 3.53	m	
H6'	3.61 – 3.53	m	
OCH_3	3.65	s	
OCH_3	3.52	s	
OCH_3	3.42	s	
OCH_3	3.41	s	

^{1}H NMR, $CDCl_3$, 400 MHz (Conc., ~30 mg/mL)

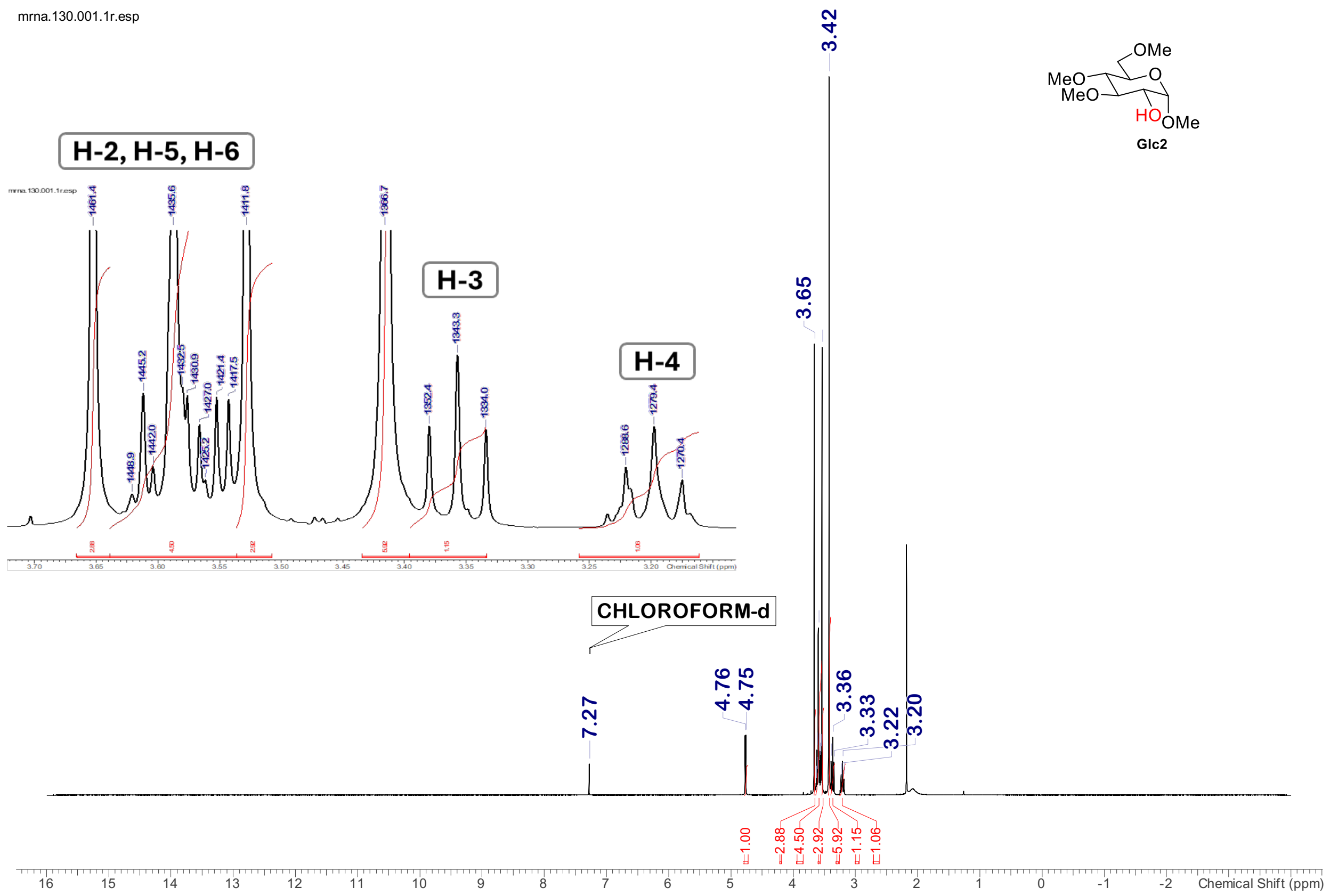

^{1}H NMR, $CDCl_3$, 400 MHz (diluted, 1mg/mL)

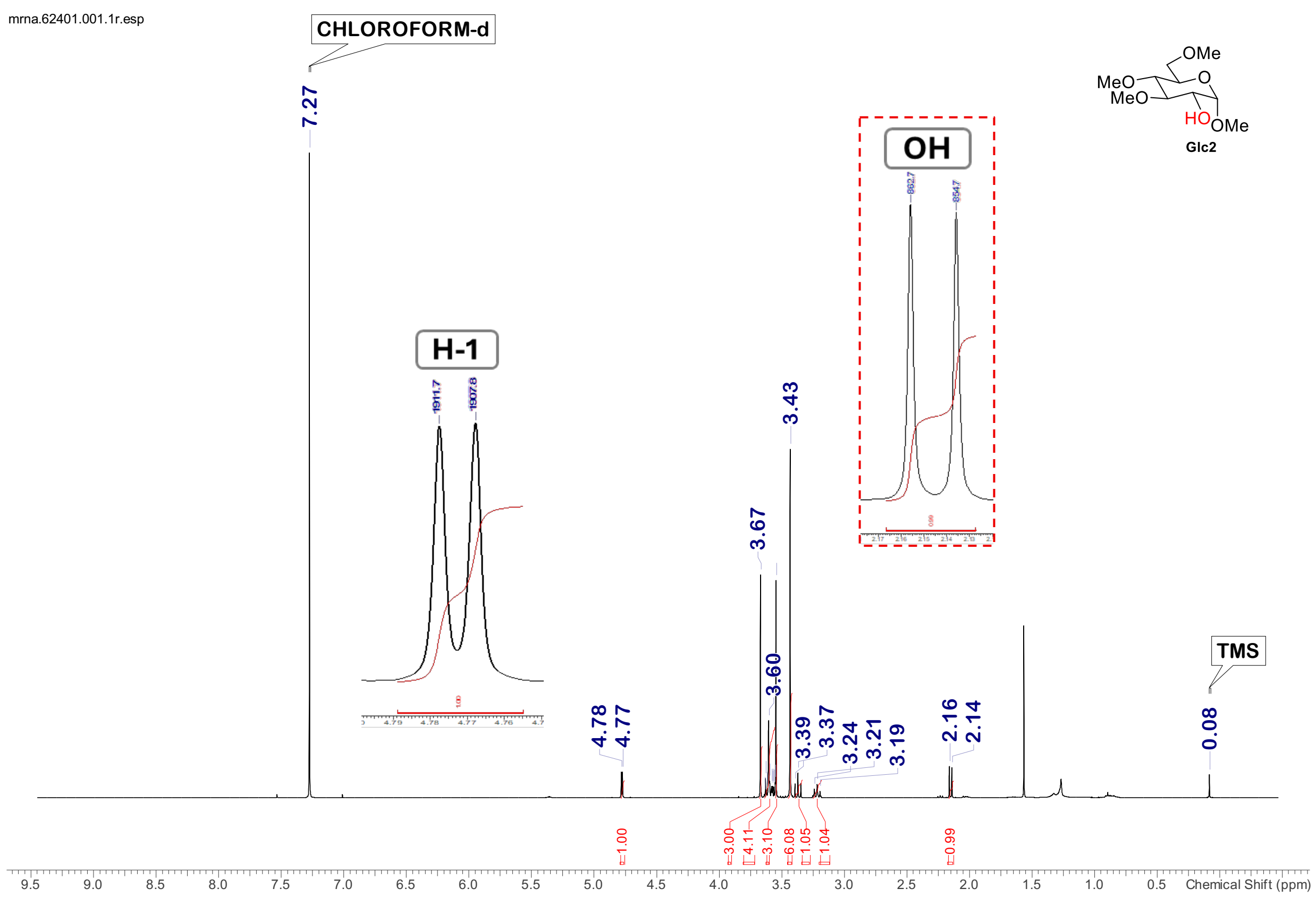

$^{13}C\{^1H\}$ NMR, $CDCl_3$, 101 MHz

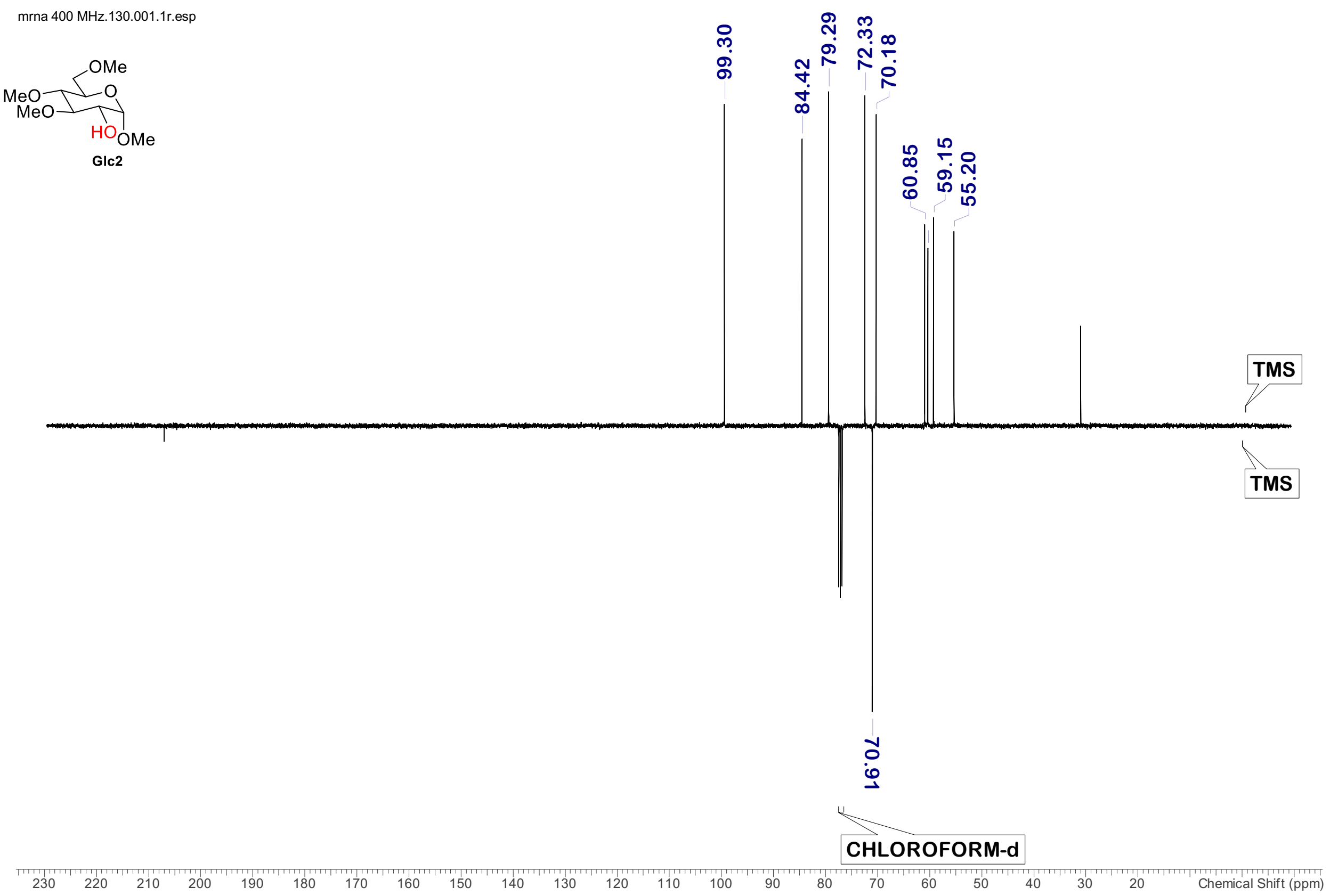

5.6 Methyl 2,4,6-tri-*O*-methyl-α-D-glucopyranoside (Glc3), in $CDCl_3$.

^{1}H NMR (400 MHz, $CDCl_3$))

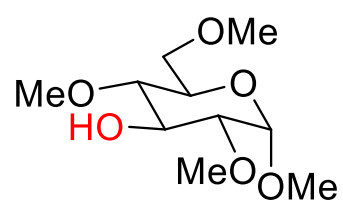

Glc3

Nucleus	δ (ppm)	m	*J* (Hz)
**OH**	2.43	d	$^3J_{OH\text{-}H3}$ = 2.1
H1	4.84	d	$^3J_{H1\text{-}H2}$ = 3.5
H2	3.23	dd	$^3J_{H2\text{-}H1}$ = 3.5, $^3J_{H2\text{-}H3}$ = 9.6
H3	3.90	app. dt	$^3J_{H3\text{-}H2}$ = $^3J_{H3\text{-}H4}$ = 9.3, $^3J_{H3\text{-}OH}$ = 2.03
H4	3.29 – 3.22	m	
H5	3.64 – 3.56	m	
H6	3.64 – 3.56	m	
H6’	3.64 – 3.56	m	
OCH_3	3.55	s	
OCH_3	3.48	s	
OCH_3	3.41	s	
OCH_3	3.40	s	

^{1}H NMR, $CDCl_3$, 400 MHz (Conc., ~30 mg/mL)

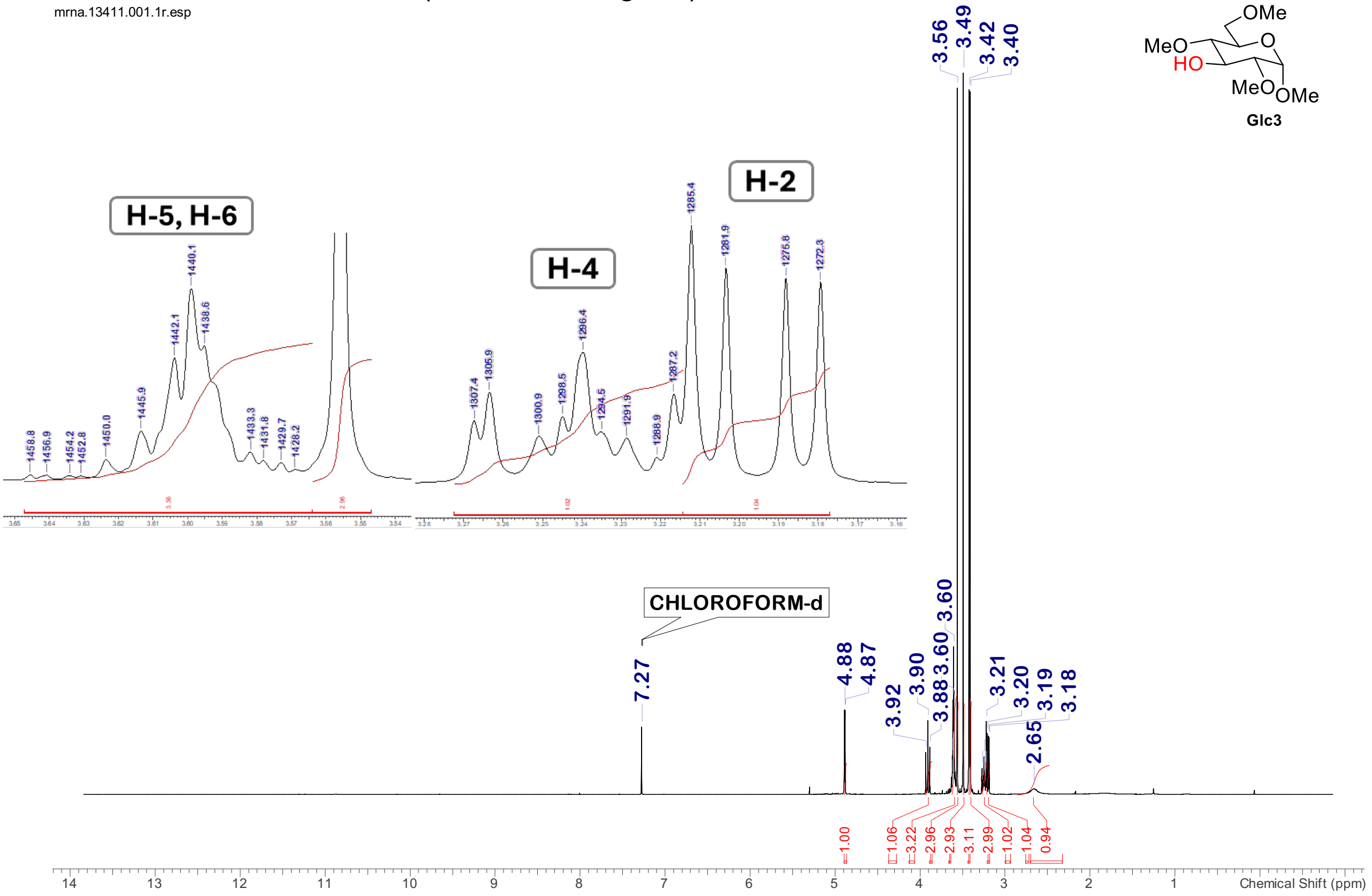

^{1}H NMR, $CDCl_3$, 400 MHz (diluted, 1mg/mL)

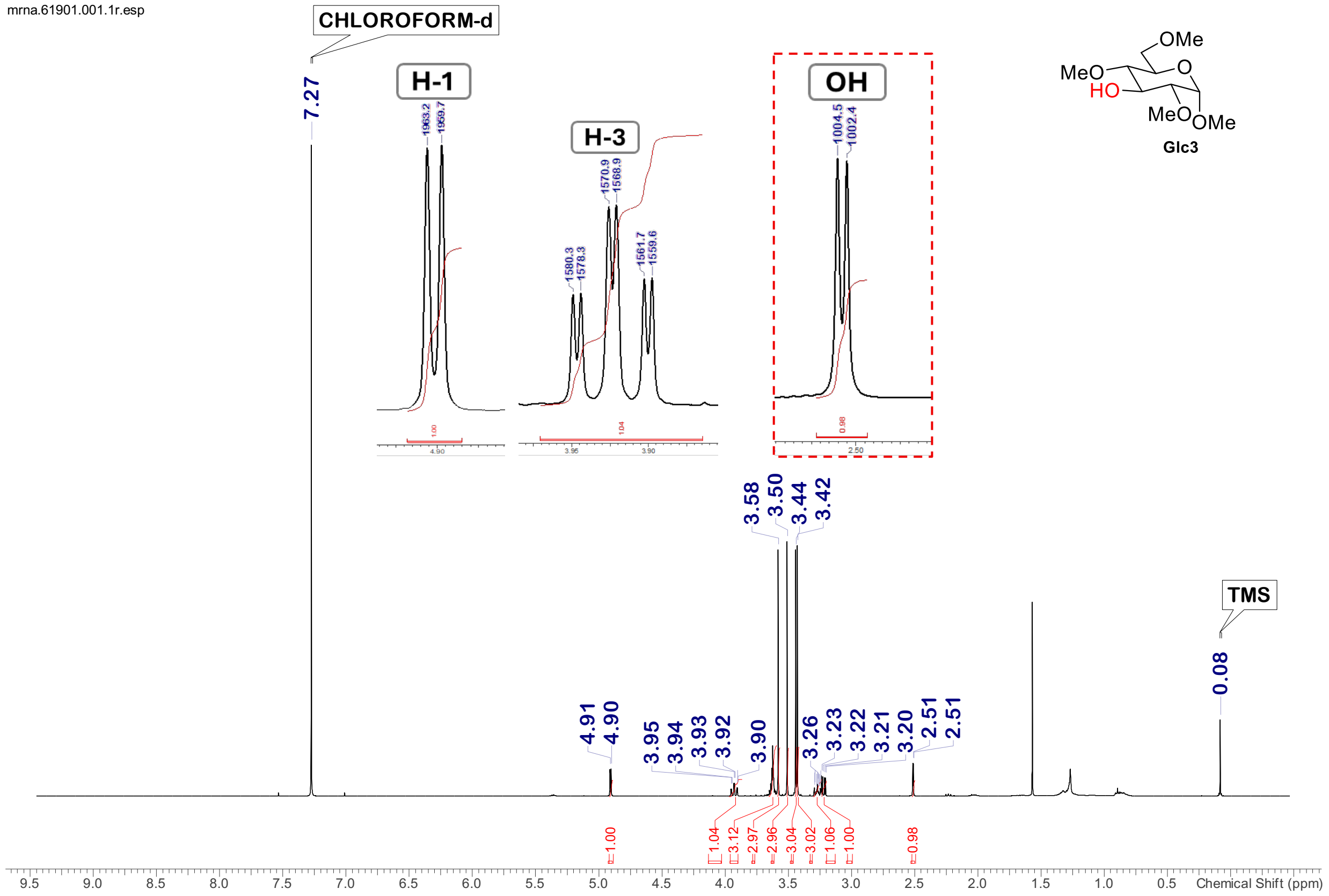

$^{13}C\{^{1}H\}$ NMR, $CDCl_3$, 101 MHz

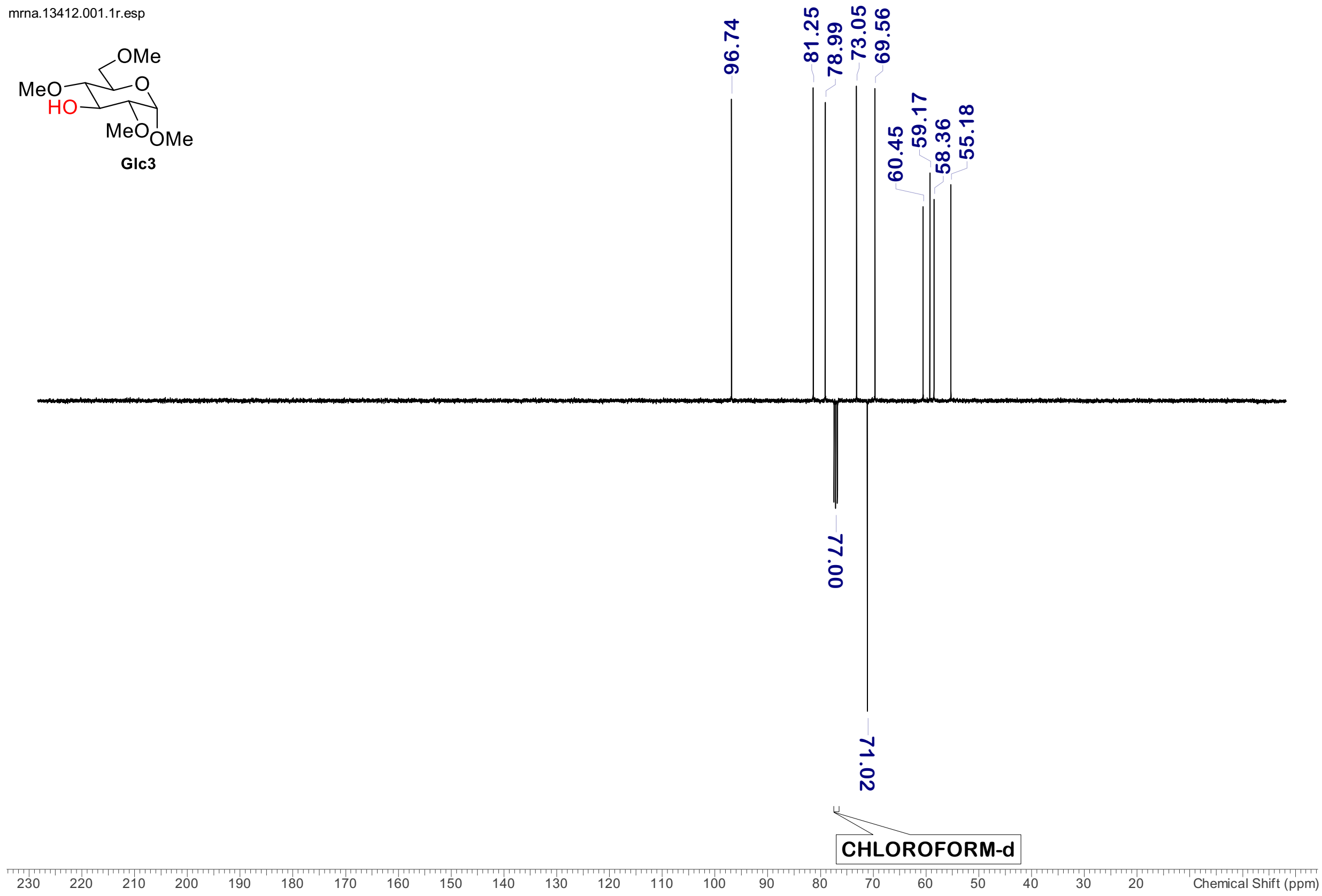

5.7 Methyl 2,3,6-tri-*O*-methyl-α-D-glucopyranoside (Glc4), in $CDCl_3$.

^{1}H NMR (400 MHz, $CDCl_3$))

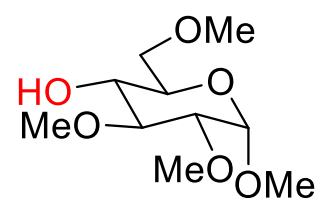

Glc4

Nucleus	δ (ppm)	m	*J* (Hz)
**OH**	2.79	d	$^{3}J_{OH\text{-}H4}$ = 1.1
H1	4.84	d	$^{3}J_{H1\text{-}H2}$ = 3.5
H2	3.23	dd	$^{3}J_{H2\text{-}H1}$ = 3.5, $^{3}J_{H2\text{-}H3}$ = 9.3
H3	3.44	t	$^{3}J_{H3\text{-}H2}$ = $^{3}J_{H3\text{-}H4}$ = 9.2
H4	3.50	td	$^{3}J_{H4\text{-}H3}$ = $^{3}J_{H4\text{-}H5}$ = 9.0, $^{3}J_{H4\text{-}OH}$ = 1.1
H5	3.71	ddd	$^{3}J_{H5\text{-}H4}$ = 9.2, $^{3}J_{H5\text{-}H6}$ = 4.1, $^{3}J_{H5\text{-}H6'}$ = 3.6
H6	3.58 – 3.64	m	with a $OC\underline{H}_3$ singlet at 3.62 ppm
H6’	3.58 – 3.64	m	with a $OC\underline{H}_3$ singlet at 3.62 ppm
OCH_3	3.62	s	
OCH_3	3.48	s	
OCH_3	3.43	s	
OCH_3	3.40	s	

^{1}H NMR, $CDCl_3$, 400 MHz (Conc.)

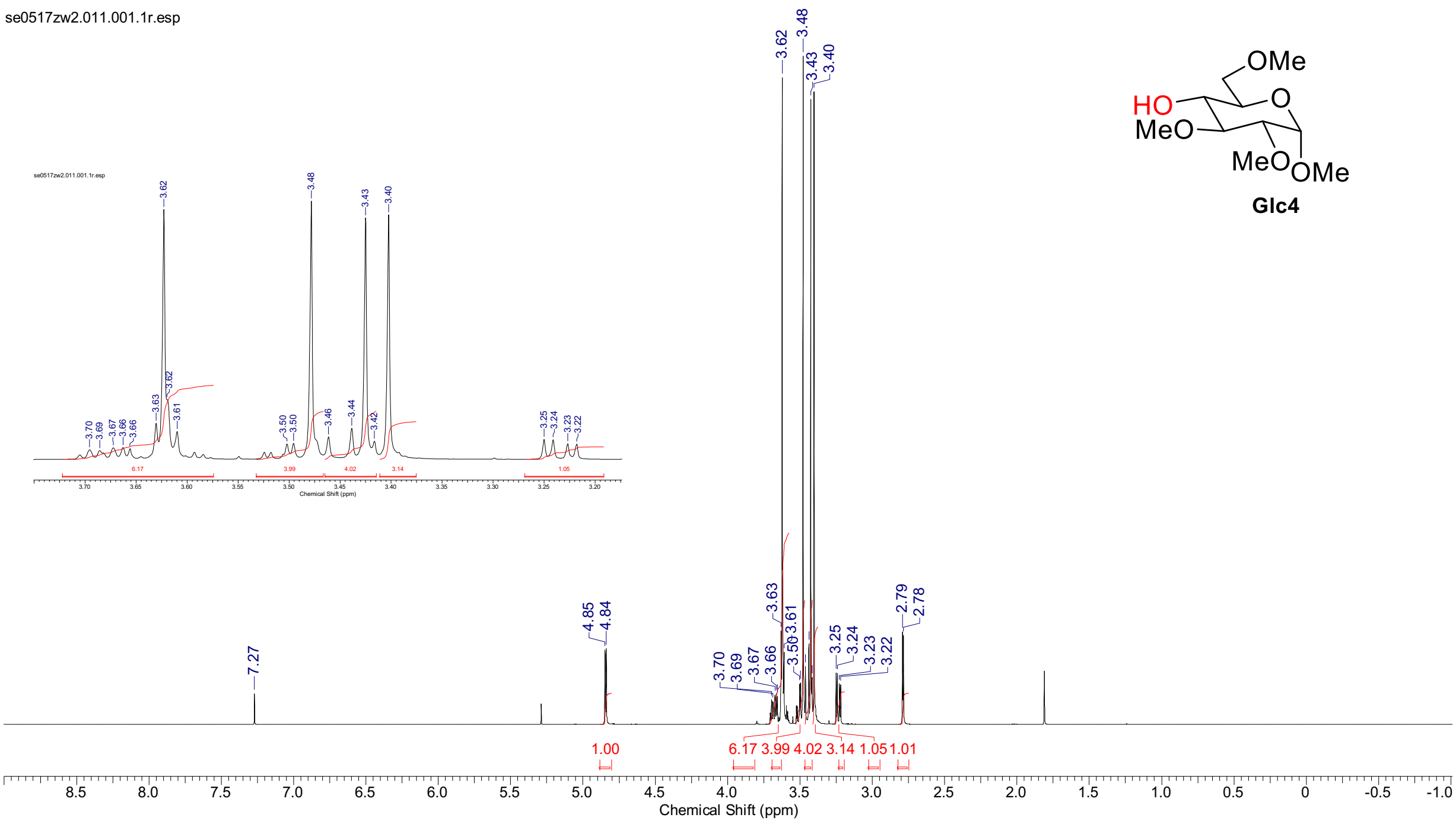

^{13}C{^{1}H} NMR, $CDCl_3$, 101 MHz

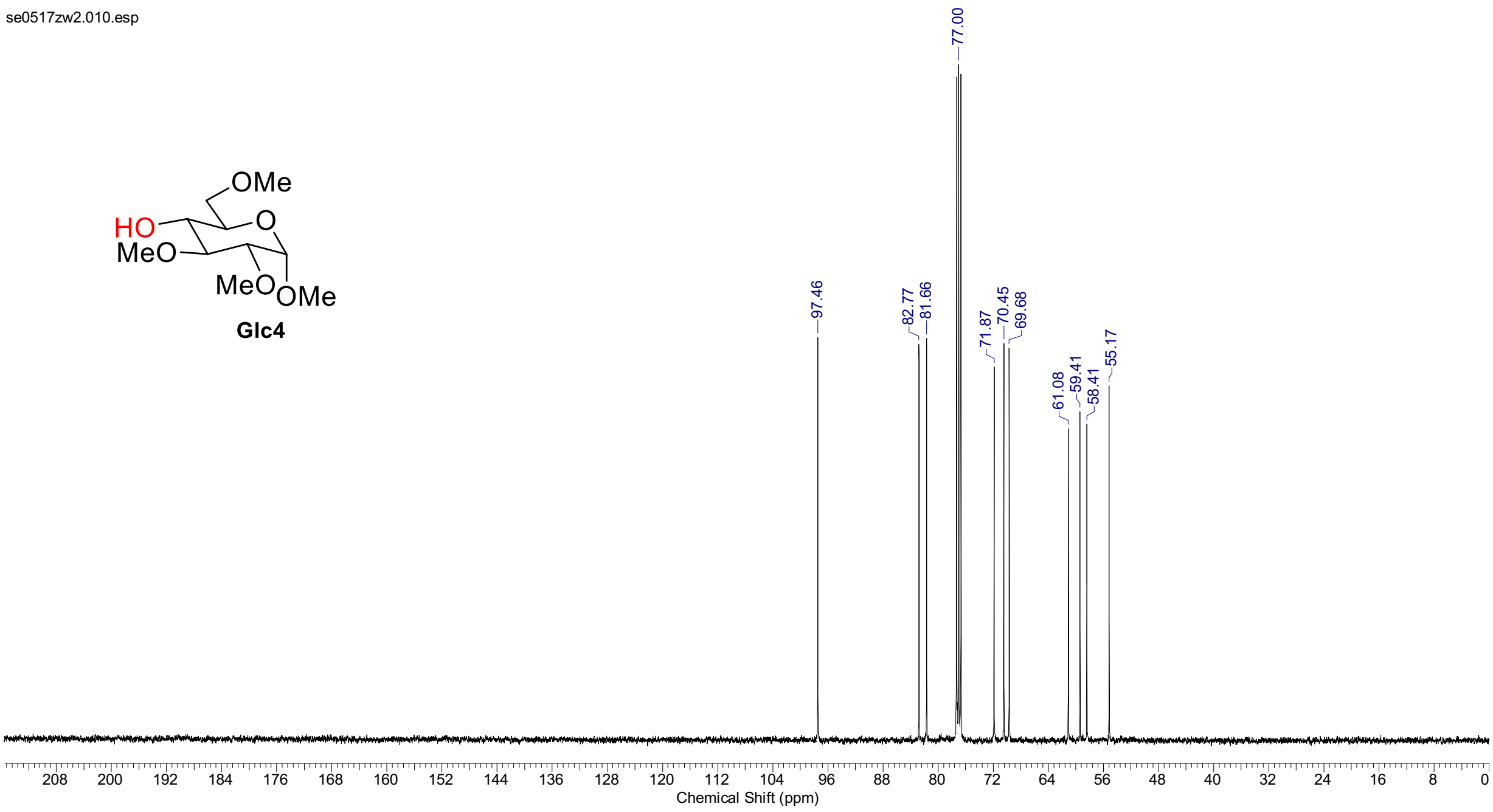

5.8 Methyl 2,3,4-tri-*O*-methyl-α-D-glucopyranoside (Glc6), in $CDCl_3$

^{1}H NMR (400 MHz, $CDCl_3$))

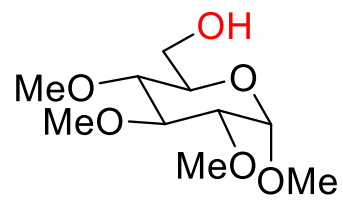

Glc6

Nucleus	δ (ppm)	m	*J* (Hz)
**OH**	1.83	dd	$^{3}J_{OH\text{-}H6}$ = 5.1, $^{3}J_{OH\text{-}H6'}$ = 7.8
H1	4.81	d	$^{3}J_{H1\text{-}H2}$ = 3.6
H2	3.20 – 3.13	m	
H3	3.58 – 3.51	m	
H4	3.20 – 3.13	m	
H5	3.58 – 3.51	m	
H6	3.83	ddd	$^{3}J_{H6\text{-}H5}$ = 2.9, $^{3}J_{H6\text{-}H6'}$ = 11.8, $^{3}J_{H6\text{-}OH}$ = 5.1
H6’	3.75	ddd	$^{3}J_{H6'\text{-}H5}$ = 4.2, $^{3}J_{H6'\text{-}H6}$ = 11.7, $^{3}J_{H6'\text{-}OH}$ = 7.8
OCH_3	3.64	s	
OCH_3	3.58	s	
OCH_3	3.53	s	
OCH_3	3.42	s	

^{1}H NMR, $CDCl_3$, 400 MHz

mrna.62503.001.1r.esp

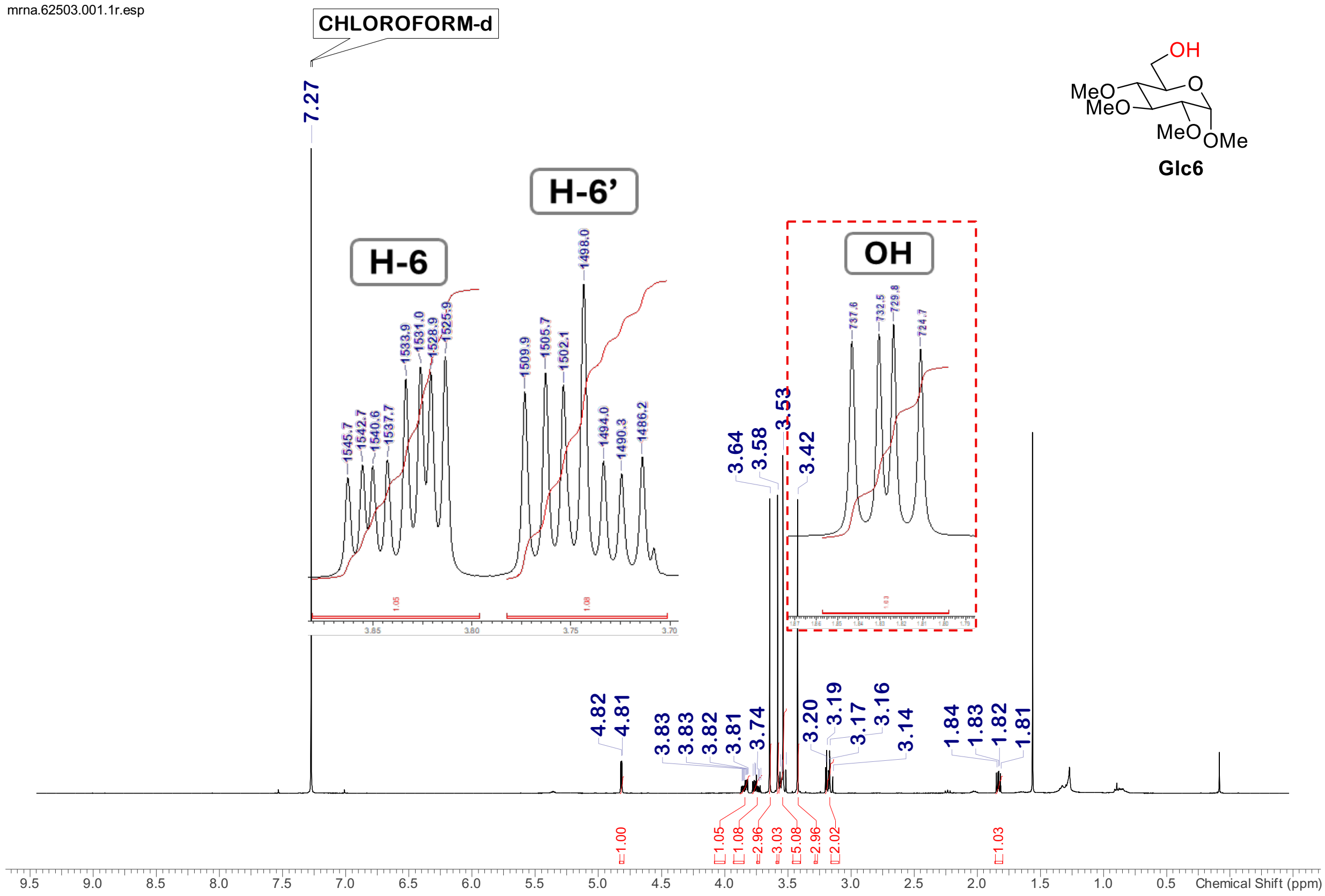

$^{13}C\{^1H\}$ NMR, $CDCl_3$, 101 MHz

mrna 400 MHz.126.001.1r.esp

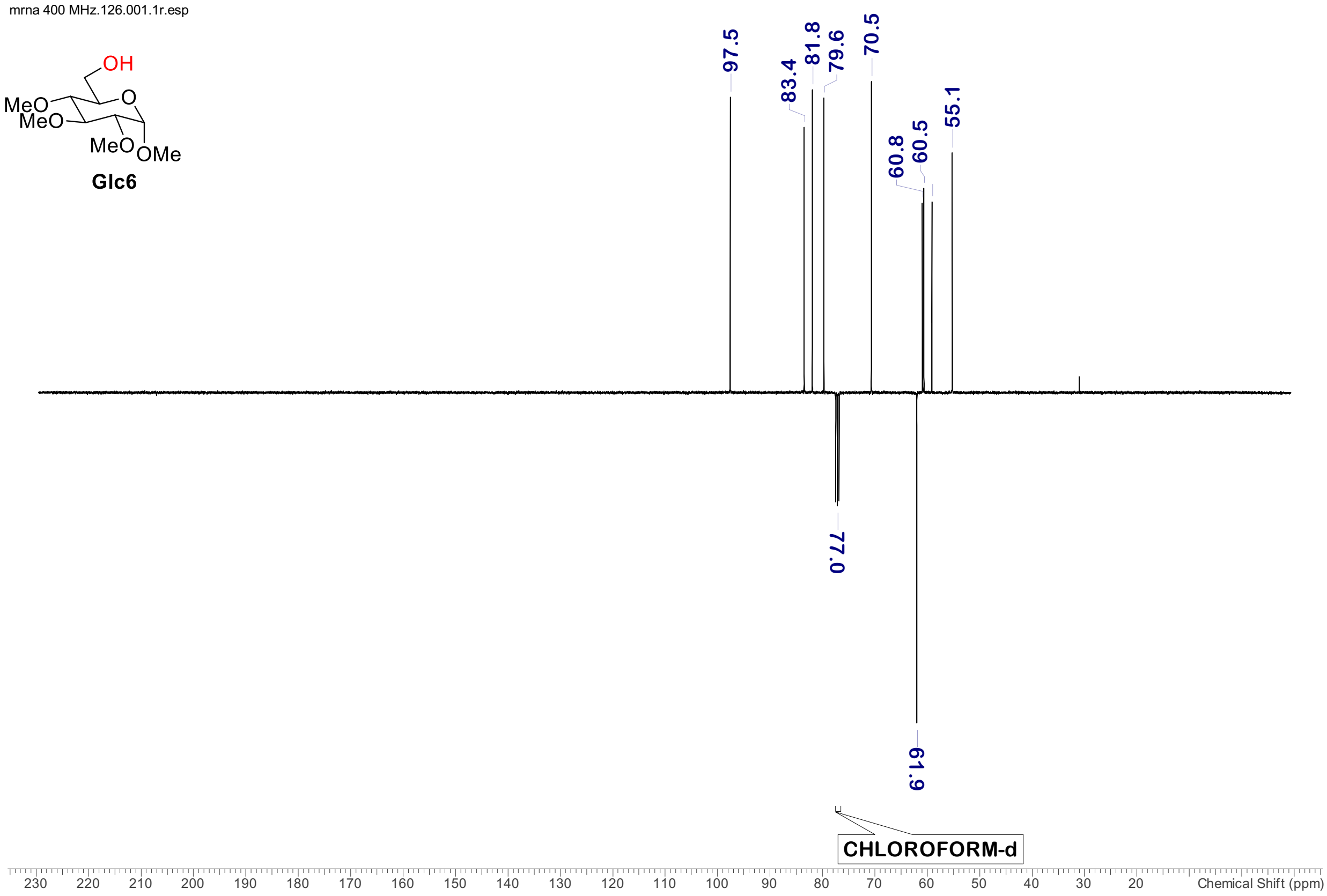

5.9 Methyl 3,4,6-tri-*O*-methyl-α-D-galactopyranoside (Gal2), in C_6D_6

^{1}H NMR (400 MHz, C_6D_6, COSY-HSQC)

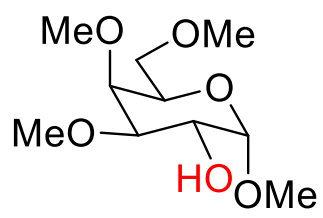

Gal2

Nucleus	δ (ppm)	m	*J* (Hz)
**OH**	2.07	br d	$^3J_{OH\text{-}H2}$ = 7.2
H1	4.70	d	$^3J_{H1\text{-}H2}$ = 3.9
H2	4.29	app. ddd	$^3J_{H2\text{-}H1}$ = 3.9, $^3J_{H2\text{-}H3}$ = 10.0, $^3J_{H2\text{-}OH}$ = 6.9
H3	3.33	dd	$^3J_{H3\text{-}H2}$ = 10.0, $^3J_{H3\text{-}H4}$ = 2.9
H4	3.54-3.53	m	with H-6’ overlap at 3.53 ppm
H5	3.87	app. dt	$^3J_{H5\text{-}H6}$ = $^3J_{H5\text{-}H6'}$ = 6.7, $^3J_{H5\text{-}H4}$ = 0.6
H6	3.62	dd	$^3J_{H6\text{-}H5}$ = 7.0, $^3J_{H6\text{-}H6'}$ = 9.2,
H6’	3.53	dd	$^3J_{H6'\text{-}H5}$ = 5.9, $^3J_{H6'\text{-}H6}$ = 9.2
OCH_3	3.45	s	
OCH_3	3.26	s	
OCH_3	3.13	s	
OCH_3	3.10	s	

^{1}H NMR, C_6D_6, 400 MHz

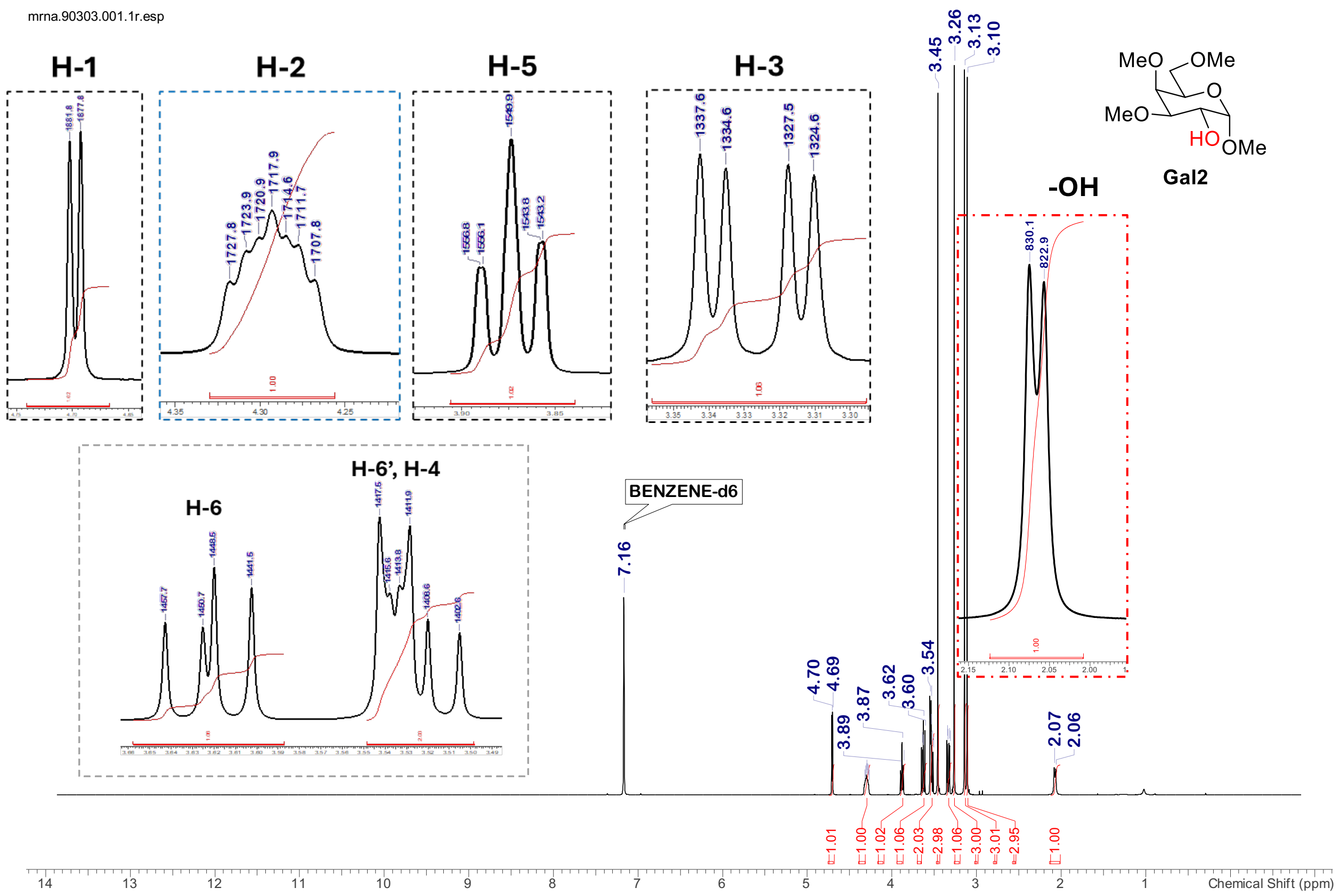

^{13}C NMR, C_6D_6, 101 MHz

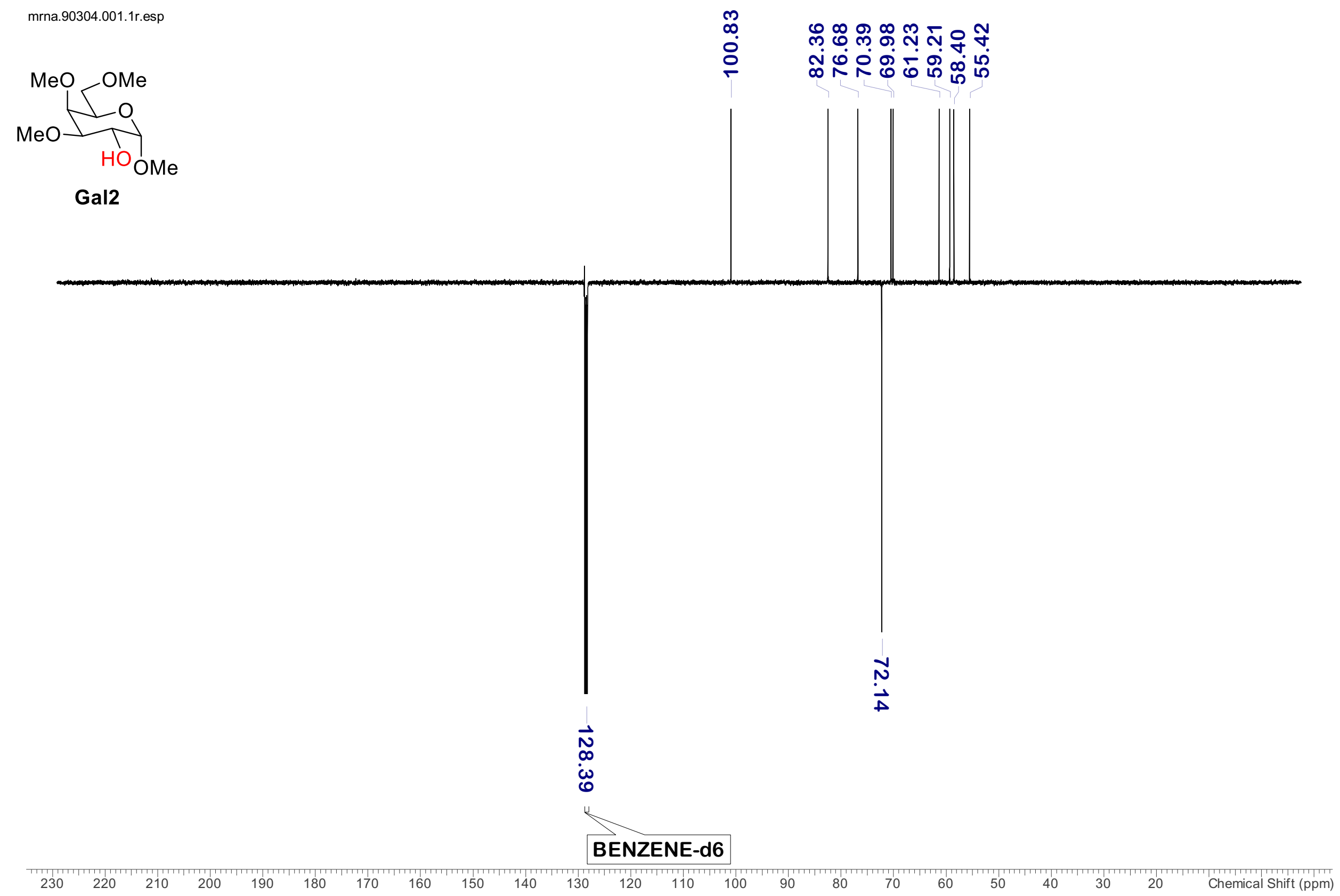

5.10 Methyl 3,4,6-tri-*O*-methyl-α-D-galactopyranoside (Gal2), in $CDCl_3$

^{1}H NMR (400 MHz, C_6D_6, COSY-HSQC)

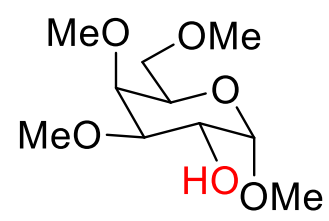

Gal2

Nucleus	δ (ppm)	m	*J* (Hz)
**OH**	2.24	d	$^3J_{OH\text{-}H2}$ = 6.9
H1	4.82	d	$^3J_{H1\text{-}H2}$ = 3.9
H2	4.98	app. ddd	$^3J_{H2\text{-}H1}$ = 3.9, $^3J_{H2\text{-}H3}$ = 10.2, $^3J_{H2\text{-}OH}$ = 6.6
H3	3.39 - 3.43	m	with two OC*H*$_3$ singlets at 3.42 and 3.40 ppm
H4	3.72	app d	$^3J_{H4\text{-}H3}$ = 2.5
H5	3.85	app. t	$^3J_{H5\text{-}H6}$ = $^3J_{H5\text{-}H6'}$ = 6.6
H6	3.48 - 3.59	m	with two OC*H*$_3$ singlets at 3.55 and 3.52 ppm
H6’	3.48 - 3.59	m	with two OC*H*$_3$ singlets at 3.55 and 3.52 ppm
OCH_3	3.55	s	
OCH_3	3.52	s	
OCH_3	3.42	s	
OCH_3	3.40	s	

^{1}H NMR, $CDCl_3$, 400 MHz

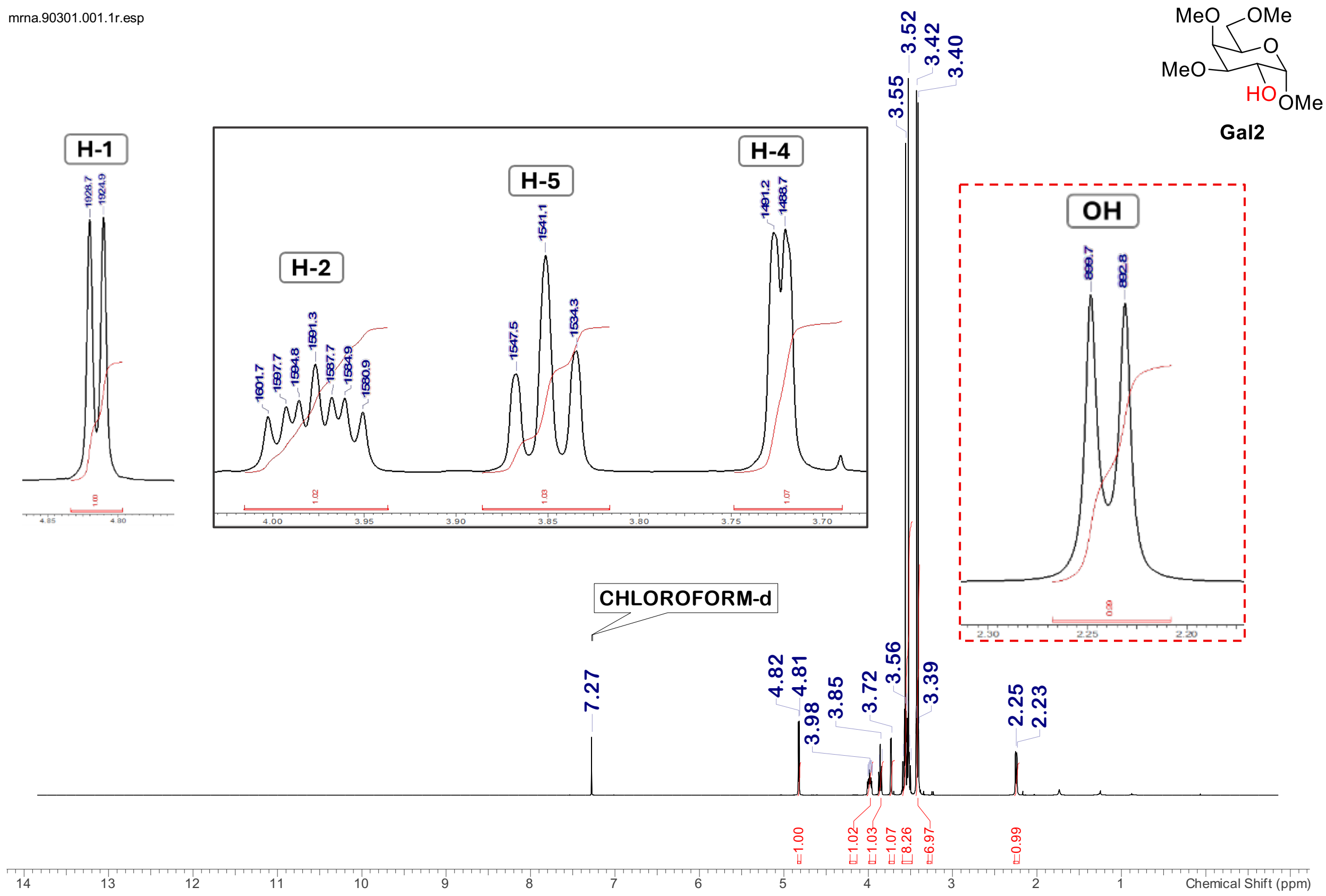

^{13}C NMR, $CDCl_3$, 101 MHz

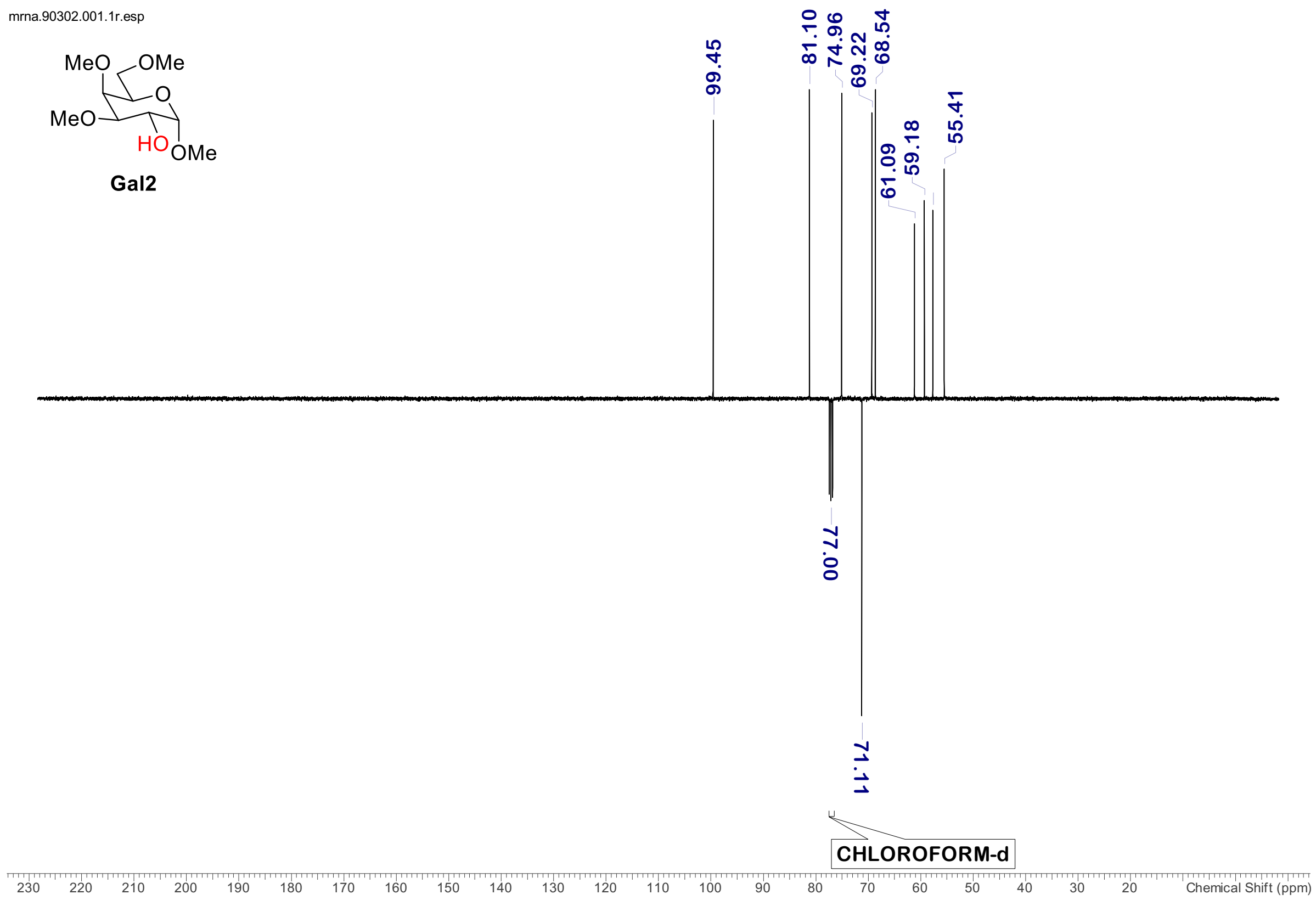

5.11 Methyl 2,4,6-tri-*O*-methyl-α-D-galactopyranoside (Gal3), in $CDCl_3$

^{1}H NMR (400 MHz $CDCl_3$, COSY-HSQC)

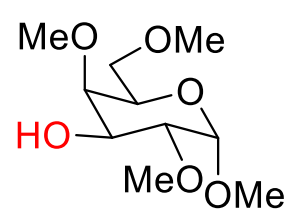

Gal3

Nucleus	δ (ppm)	m	*J* (Hz)
**OH**	2.59	br d	$^{3}J_{OH\text{-}H3}$ = 5.3
H1	4.86	d	$^{3}J_{H1\text{-}H2}$ = 3.6
H2	3.44	dd	$^{3}J_{H2\text{-}H1}$ = 3.6, $^{3}J_{H2\text{-}H3}$ = 10.1, with OC*H*$_3$ singlets at 3.44 ppm
H3	3.90	ddd	$^{3}J_{H3\text{-}H2}$ = 10.1, $^{3}J_{H3\text{-}H4}$ = 4.5, $^{3}J_{H3\text{-}OH}$ = 3.5
H4	3.55	dd	$^{3}J_{H4\text{-}H3}$ = 3.4, $^{3}J_{H4\text{-}H5}$ = 1.1
H5	3.85	br td	$^{3}J_{H5\text{-}H4}$ = 0.8, $^{3}J_{H5\text{-}H6}$ = $^{3}J_{H5\text{-}H6'}$ = 6.5,
H6	3.51	dd	$^{3}J_{H6\text{-}H5}$ = 6.3, $^{3}J_{H6\text{-}H6'}$ = 9.4, with OC*H*$_3$ singlets at 3.53 ppm
H6'	3.48	dd	$^{3}J_{H6\text{-}H5}$ = 6.6, $^{3}J_{H6'\text{-}H6}$ = 9.6
OCH_3	3.53	s	
OCH_3	3.44	s	
OCH_3	3.36	s	
OCH_3	3.35	s	

^{1}H NMR, $CDCl_3$, 400 MHz

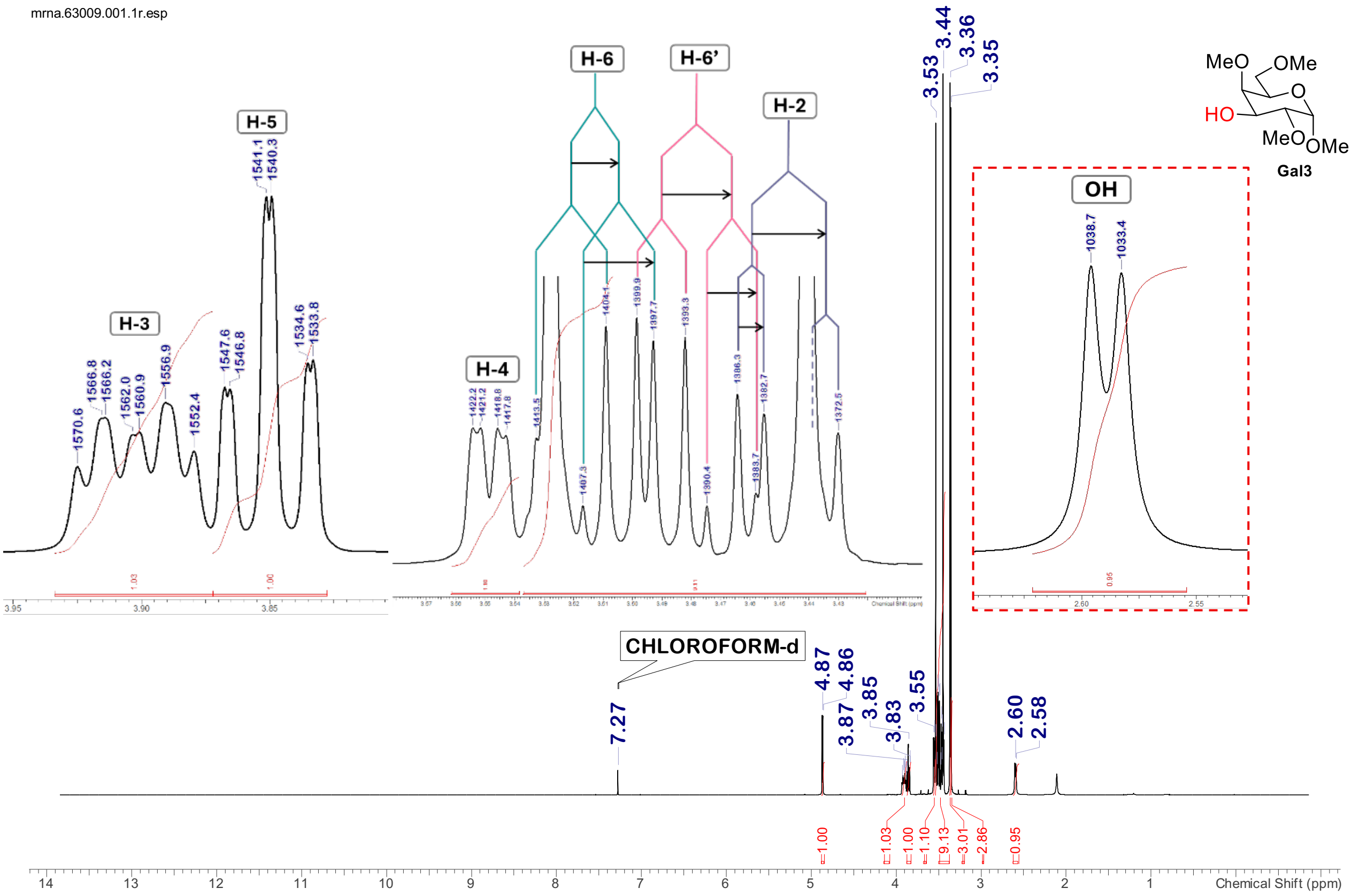

^{13}C NMR, $CDCl_3$, 101 MHz

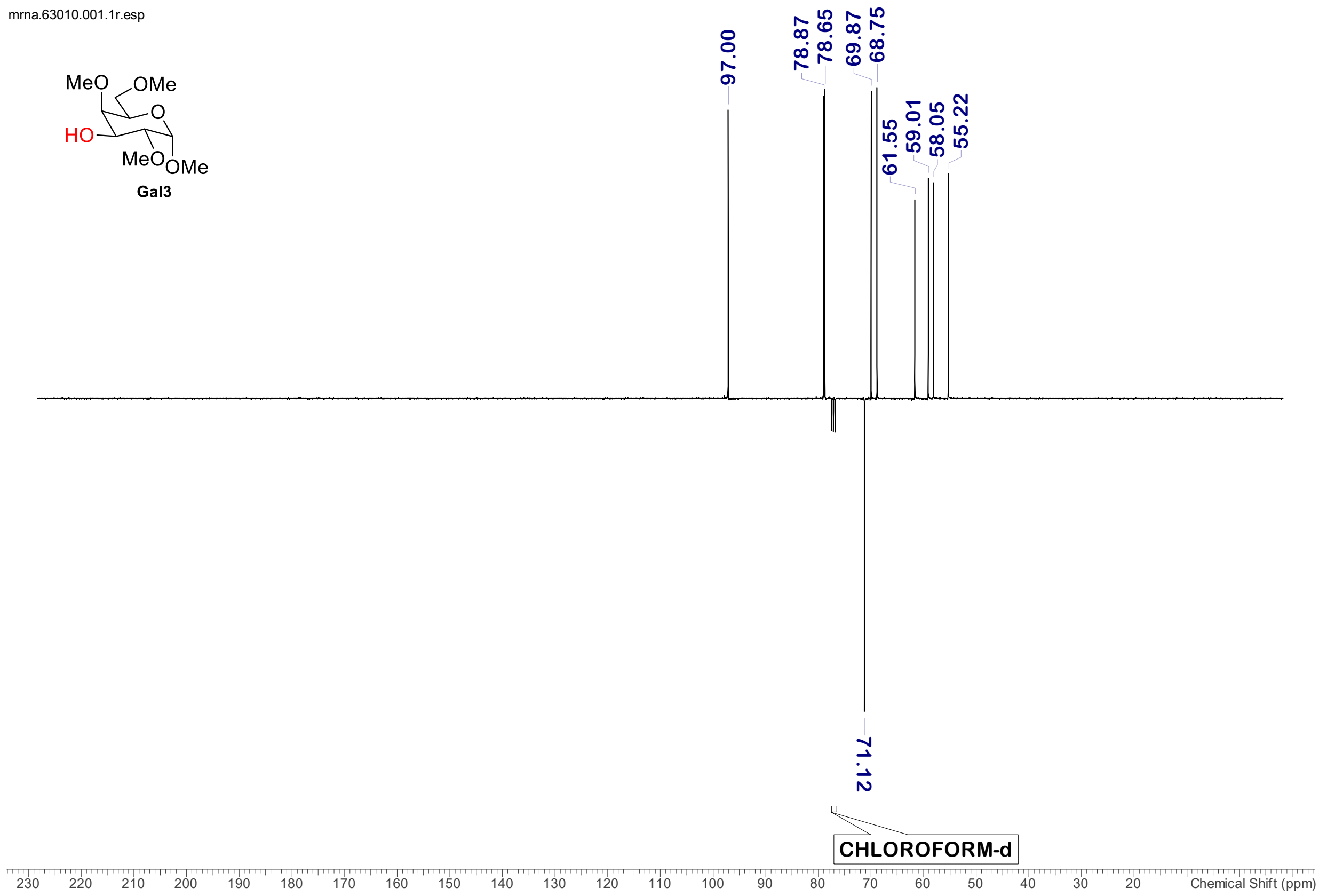

5.12 Methyl 2,3,6-tri-*O*-methyl-α-D-galactopyranoside (Gal4), in $CDCl_3$

^{1}H NMR (400 MHz $CDCl_3$, COSY-HSQC)

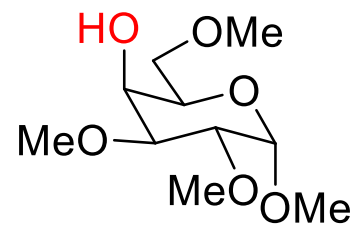

Gal4

Nucleus	δ (ppm)	m	*J* (Hz)
**OH**	2.68	t	$^3J_{OH\text{-}H4} = {}^3J_{OH\text{-}H5} = 1.4$
H1	4.90	d	$^3J_{H1\text{-}H2} = 3.5$
H2	3.59	dd	$^3J_{H2\text{-}H1} = 3.6$, $^3J_{H2\text{-}H3} = 9.9$
H3	3.53	dd	$^3J_{H3\text{-}H2} = 9.9$, $^3J_{H3\text{-}H4} = 3.2$
H4	4.11	dt	$^3J_{H4\text{-}H3} = 3.1$, $^3J_{H4\text{-}H5} = {}^3J_{H4\text{-}OH} = 1.5$
H5	3.86	app t	$^3J_{H5\text{-}H6} = {}^3J_{H5\text{-}H6'} = 5.5$
H6	3.66	dd	$^3J_{H6\text{-}H5} = 5.2$, $^3J_{H6\text{-}H6'} = 10.2$
H6'	3.65	dd	$^3J_{H6'\text{-}H5} = 6.1$, $^3J_{H6'\text{-}H6} = 10.1$
OCH_3	3.50	s	
OCH_3	3.49	s	
OCH_3	3.43	s	
OCH_3	3.41	s	

^{1}H NMR, $CDCl_3$, 400 MHz

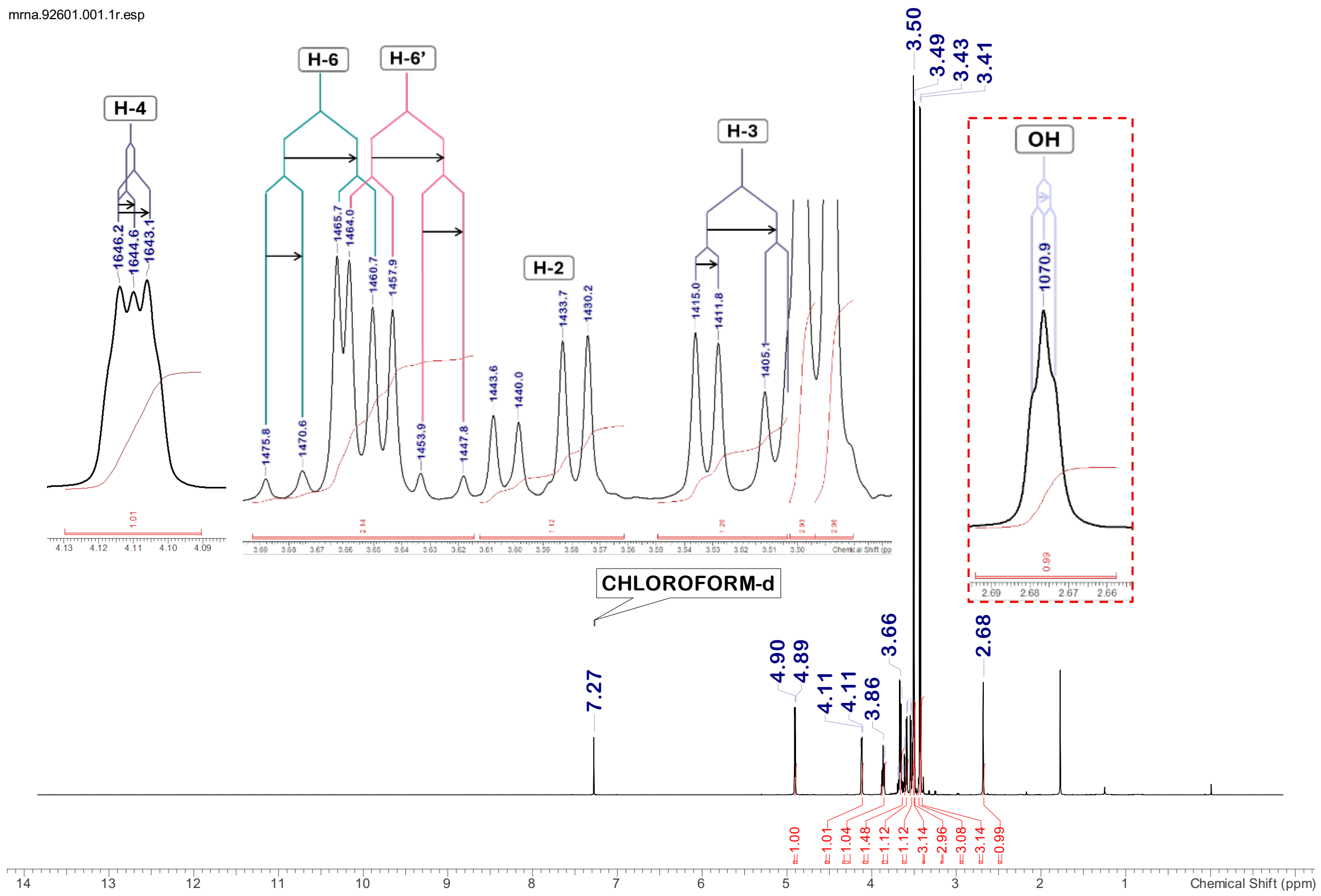

^{13}C NMR, $CDCl_3$, 101 MHz

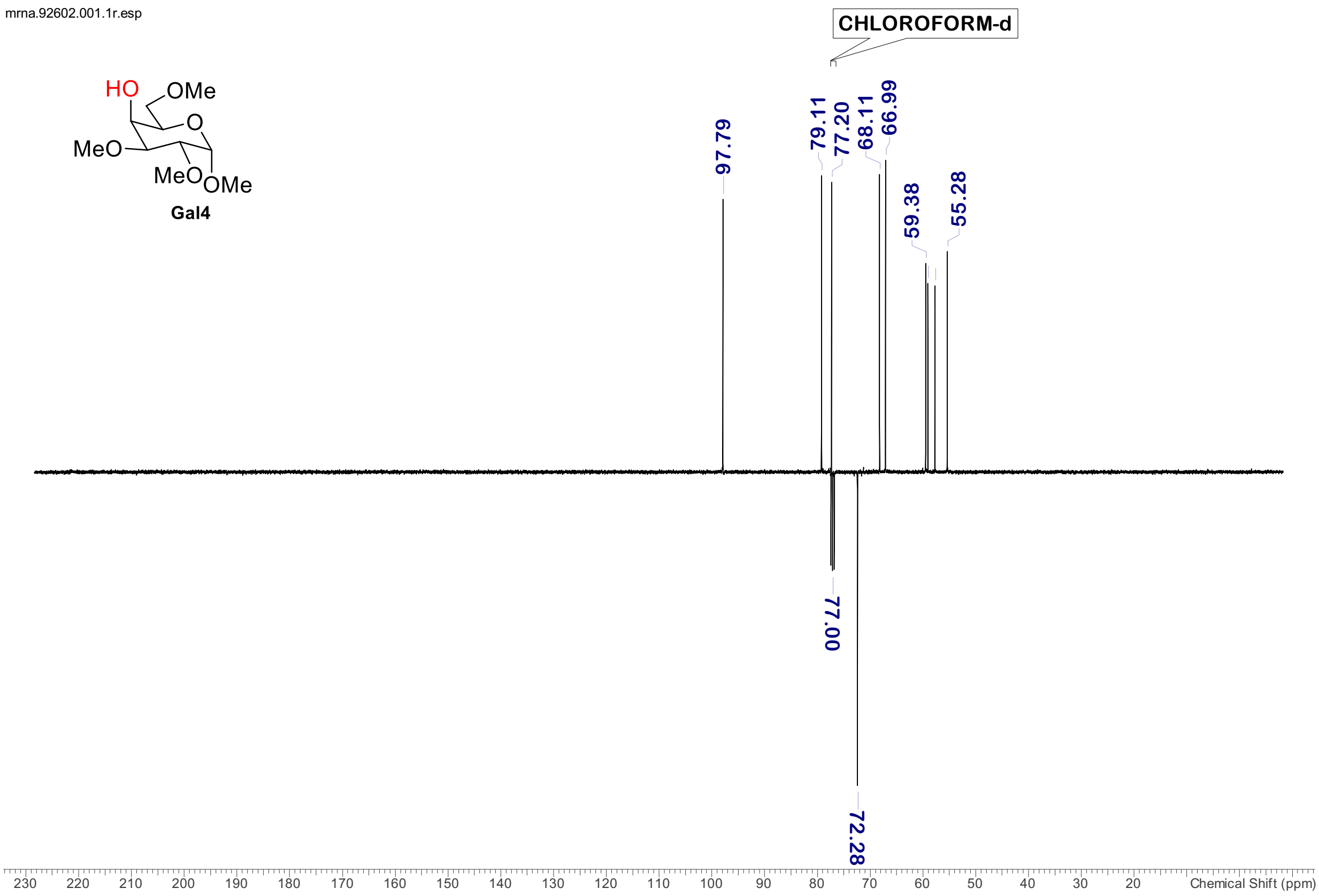

5.13 Methyl 2,3,4-tri-*O*-methyl-α-D-galactopyranoside (Gal6), in $CDCl_3$

^{1}H NMR (400 MHz $CDCl_3$, COSY-HSQCC-TSE-PSYCHEDELIC)

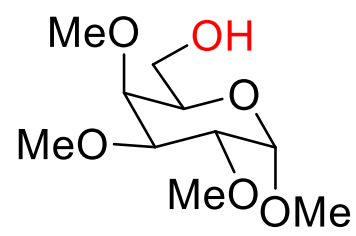

Gal6

Nucleus	δ (ppm)	m	*J* (Hz)
**OH**	2.09	dd	$^3J_{OH\text{-}H6}$ = 2.8, $^3J_{OH\text{-}H6'}$ = 8.8
H1	4.93	d	$^3J_{H1\text{-}H2}$ = 3.7
H2	3.67	dd	$^3J_{H2\text{-}H1}$ = 3.7, $^3J_{H2\text{-}H3}$ = 10.1
H3	3.57	dd	$^3J_{H3\text{-}H2}$ = 10.2, $^3J_{H3\text{-}H4}$ = 3.0
H4	3.71	dd	$^3J_{H4\text{-}H3}$ = 2.8, $^3J_{H4\text{-}H5}$ = 0.9
H5	3.80	app td	$^3J_{H5\text{-}H4}$ = 0.7, $^3J_{H5\text{-}H6}$ = $^3J_{H5\text{-}H6'}$ = 5.6
H6	3.91	ddd	$^3J_{H6\text{-}H5}$ = 6.8, $^3J_{H6\text{-}H6'}$ = 10.9, $^3J_{H6\text{-}OH}$ = 2.5
H6'	3.76	ddd	$^3J_{H6'\text{-}H5}$ = 4.7 , $^3J_{H6'\text{-}H6}$ = 10.8, $^3J_{H6'\text{-}OH}$ = 8.8
OCH_3	3.59	s	
OCH_3	3.54	s	
OCH_3	3.53	s	
OCH_3	3.44	s	

^{1}H NMR, $CDCl_3$, 400 MHz

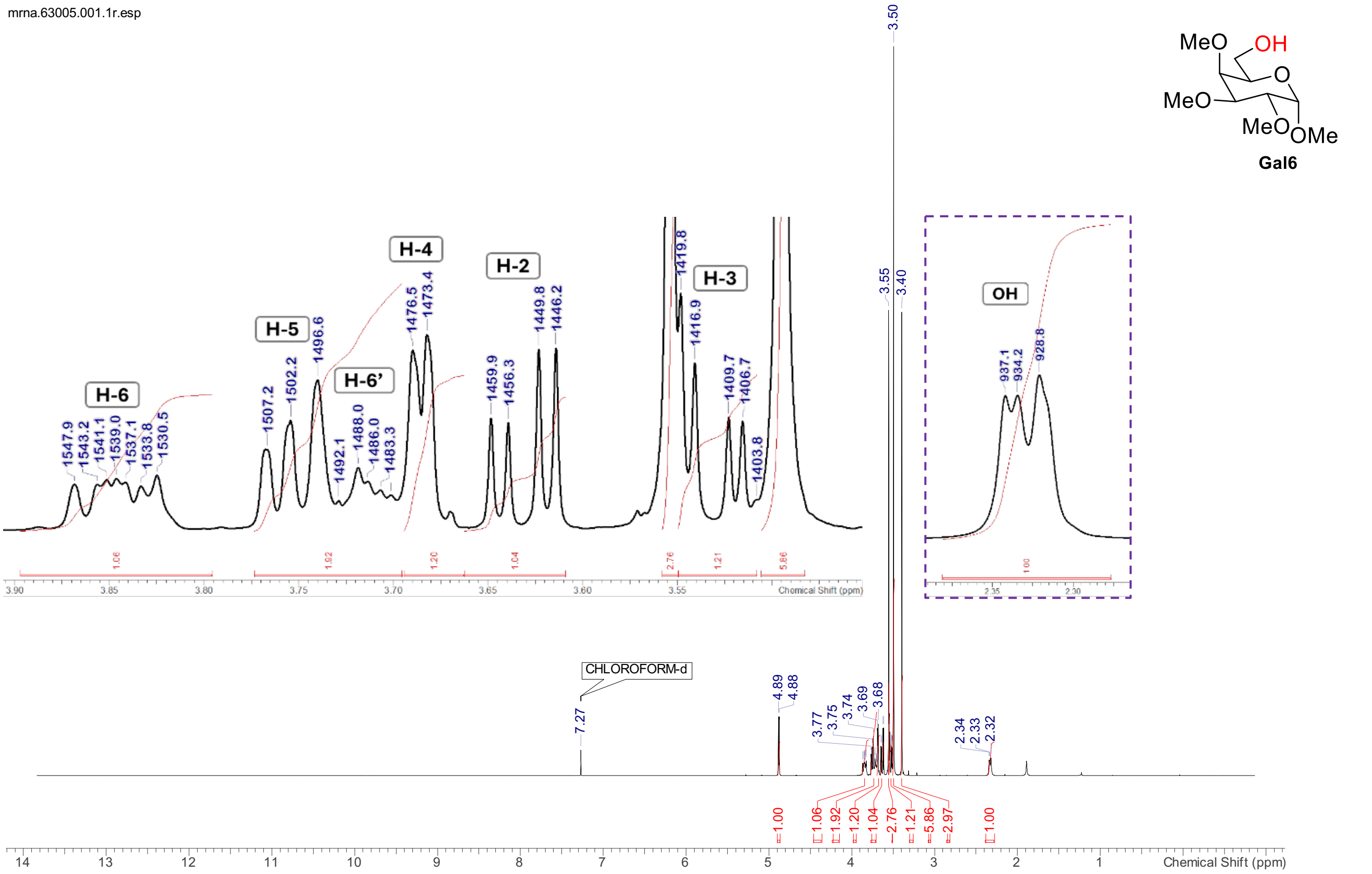

^{1}H NMR, $CDCl_3$, 600 MHz

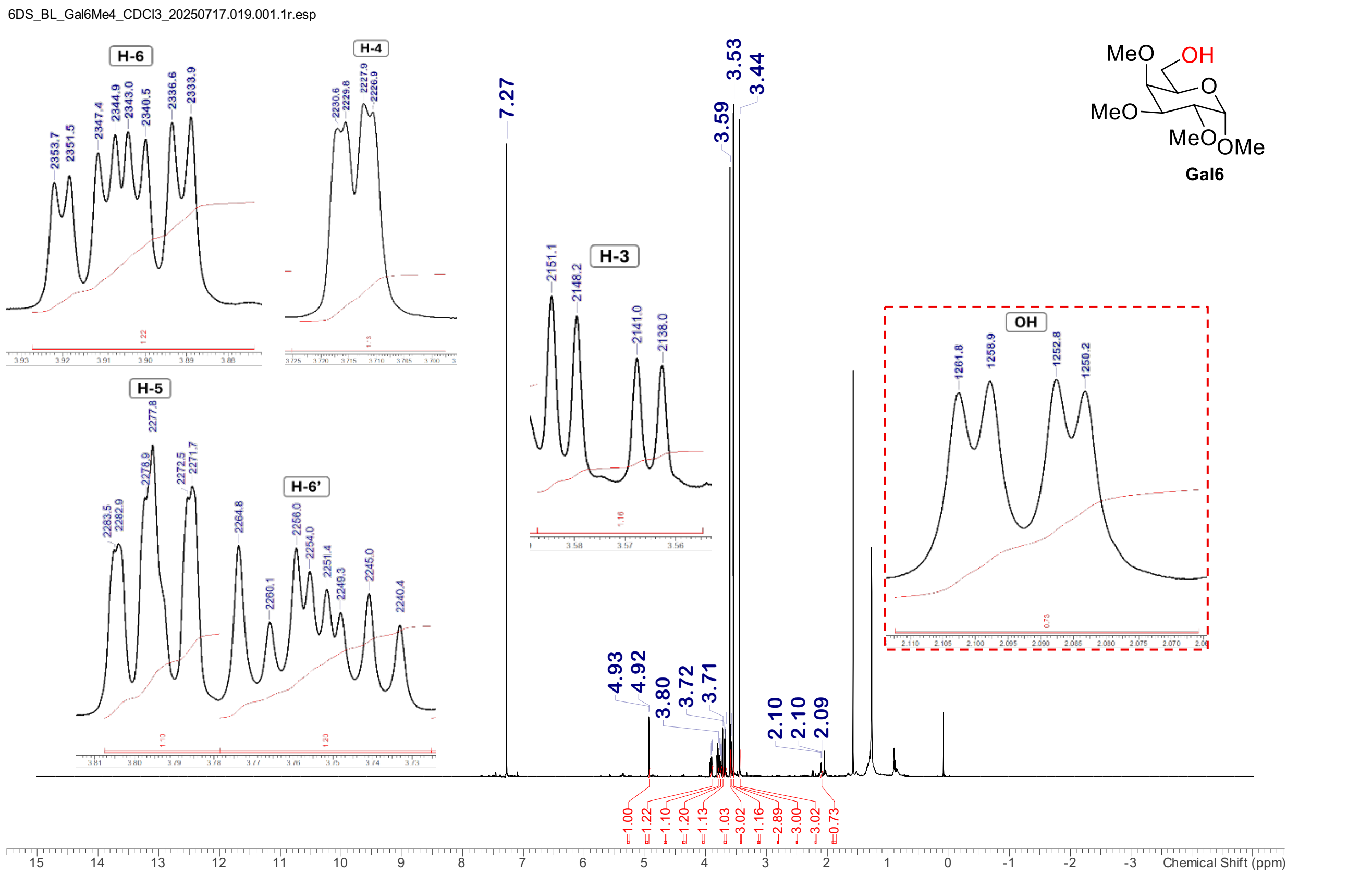

^{13}C NMR, $CDCl_3$, 101 MHz

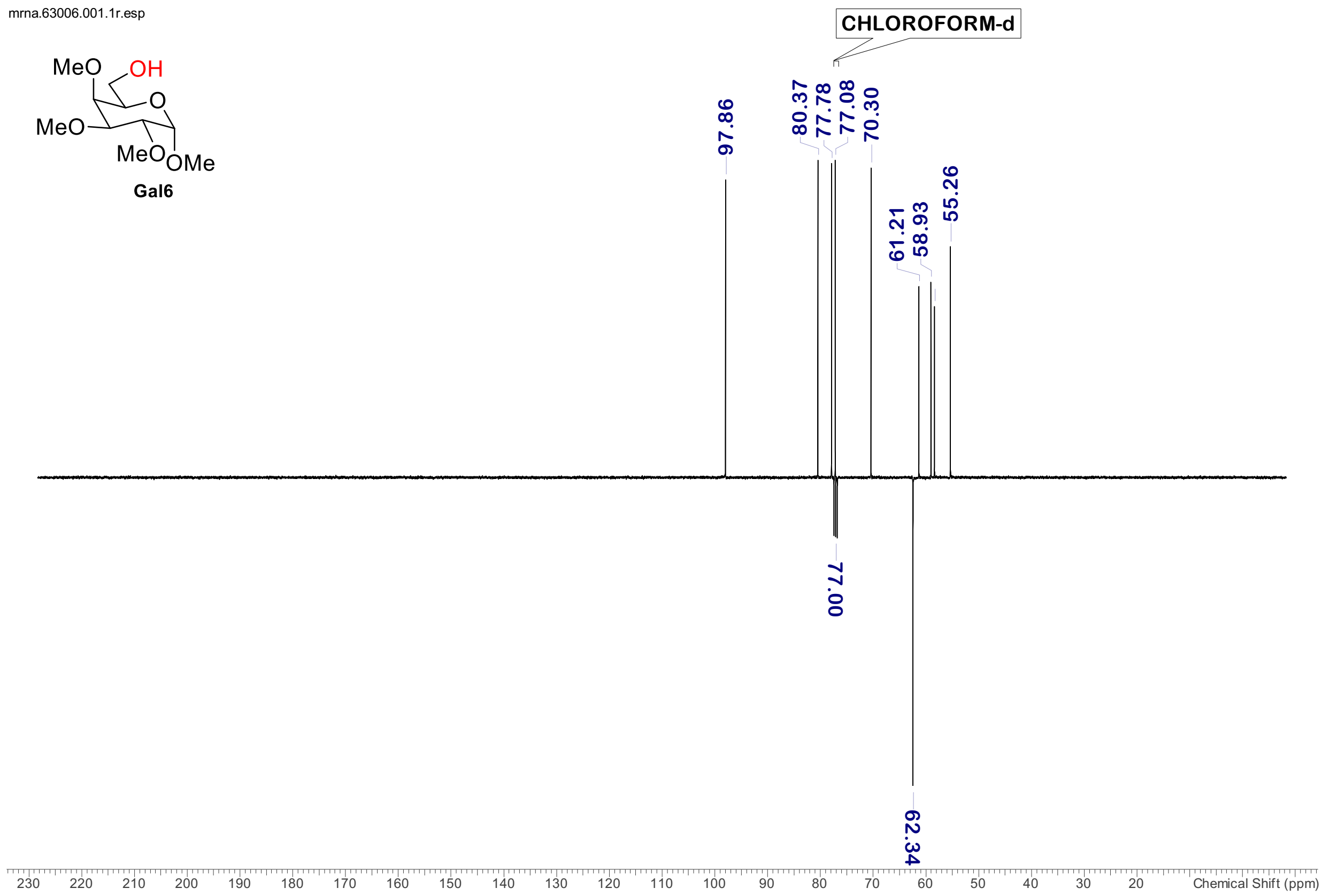

5.14 Methyl 2,3,4-tri-*O*-methyl-α-D-galactopyranoside (Gal6), in CCl_4

^{1}H NMR (600 MHz, CCl_4, Benzene-d_6 as locking solvent, COSY-HSQC-TSE-PSYCHEDELIC)

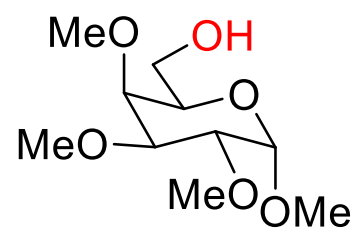

Gal6

Nucleus	δ (ppm)	m	*J* (Hz)
**OH**	1.69	dd	$^3J_{OH-H6}$ = 3.0, $^3J_{OH-H6'}$ = 8.9
H1	4.89	d	$^3J_{H1-H2}$ = 3.5
H2	3.71	dd	$^3J_{H2-H1}$ = 3.5, $^3J_{H2-H3}$ = 9.8
H3	3.64	dd	$^3J_{H3-H2}$ = 10.2, $^3J_{H3-H4}$ = 3.0
H4	3.73	dd	$^3J_{H4-H3}$ = 2.9, $^3J_{H4-H5}$ = 10.1
H5	3.80	ddd	$^3J_{H5-H4}$ = 1.4, $^3J_{H5-H6}$ = 6.5, $^3J_{H5-H6'}$ = 4.8
H6	3.91	ddd	$^3J_{H6-H5}$ = 6.8, $^3J_{H6-H6'}$ = 10.2, $^3J_{H6-OH}$ = 3.0
H6’	3.75	ddd	$^3J_{H6'-H5}$ = 4.6 , $^3J_{H6'-H6}$ = 10.6, $^3J_{H6'-OH}$ = 8.8
OCH_3	370	s	
OCH_3	3.68	s	
OCH_3	3.65	s	
OCH_3	3.57	s	

^{1}H NMR, CCl_4, 600 MHz

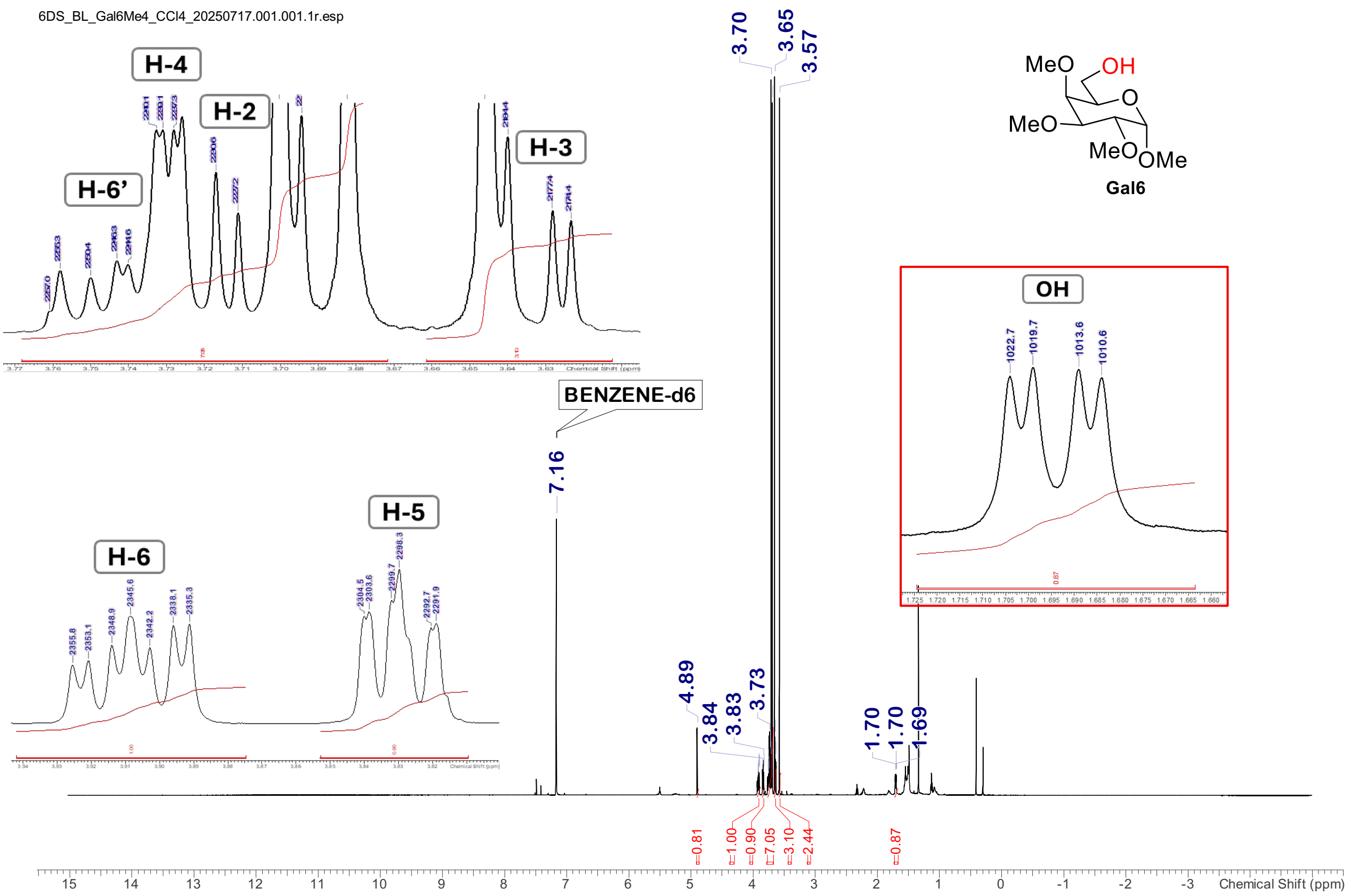

PSYCHEDELIC H-5

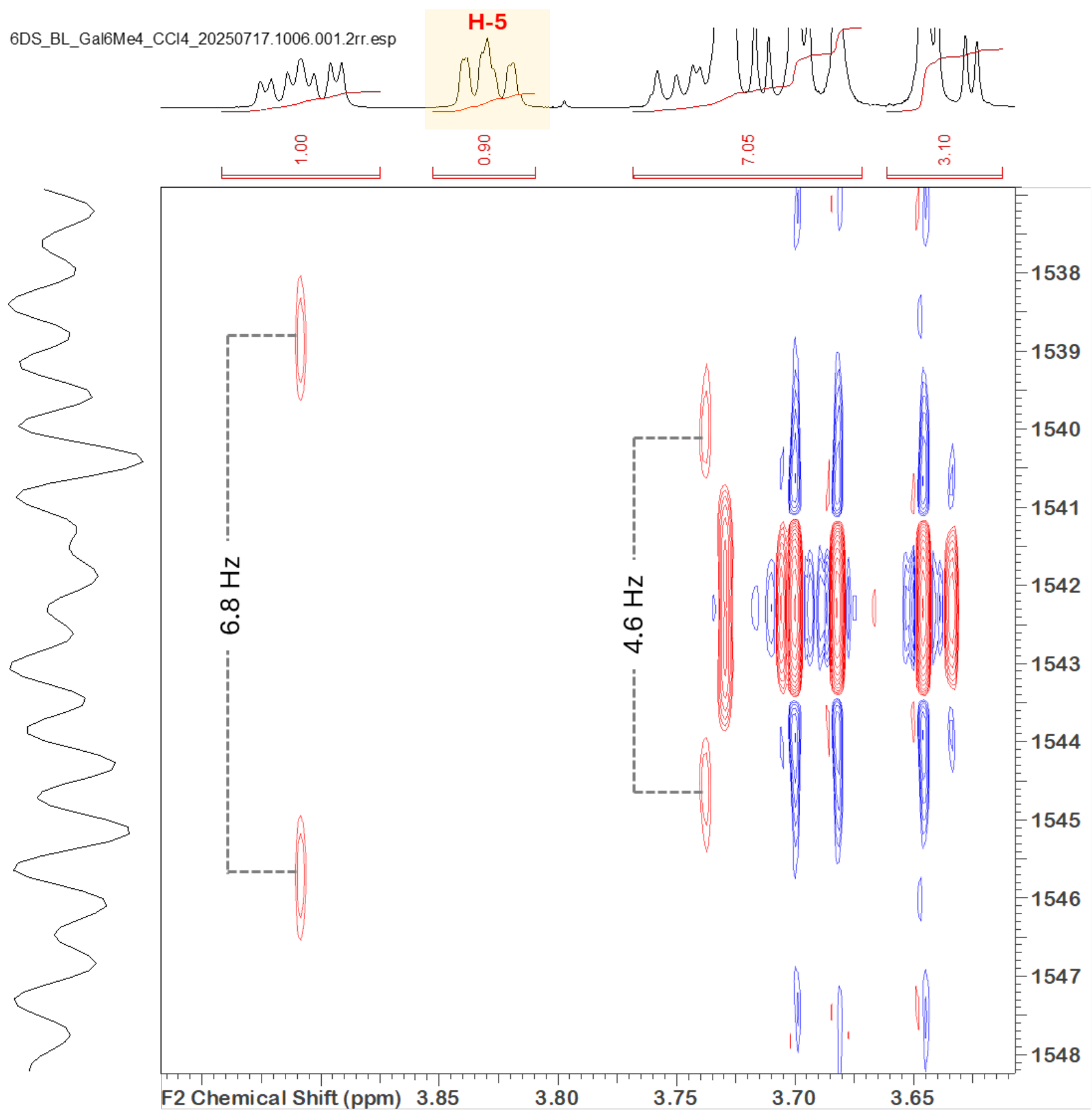

PSYCHEDELIC OH

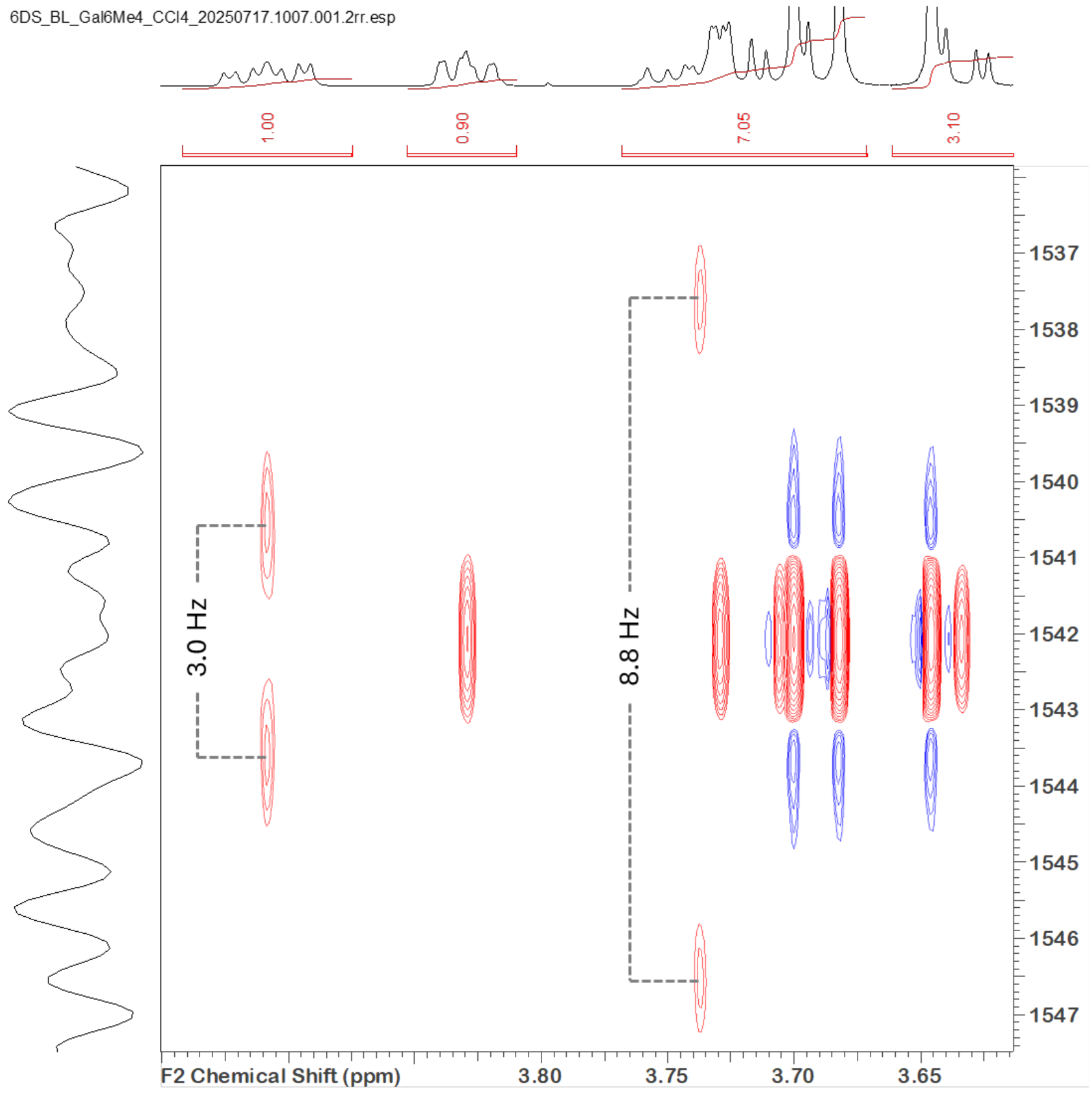

[1] C. Laurence, K. A. Brameld, J. Graton, J. Y. Le Questel, E. Renault, *J. Med. Chem.* **2009**, *52*, 4073-4086.

[2] J. Graton, F. Besseau, A.-M. Brossard, E. Charpentier, A. Deroche, J.-Y. Le Questel, *J. Phys. Chem. A* **2013**, *117*, 13184-13193.

[3] B. Linclau, J. Graton, J.-Y. Le Questel, in *Fluorine in Life Sciences: Pharmaceuticals, Medicinal Diagnostics, and Agrochemicals* (Eds.: G. Haufe, F. R. Leroux), Academic Press, **2019**, pp. 301-324.

[4] Z. Wang, G. Compain, M. Naskar, A. Goupille, L. Mtashobya, I. Murgia, J. Graton, J.-Y. Le Questel, B. Linclau, *ChemistryEurope* **2026**, *4*, e202500203.

[5] J. Graton, Z. Wang, A.-M. Brossard, D. Gonçalves Monteiro, J.-Y. Le Questel, B. Linclau, *Angew. Chem. Int. Ed.* **2012**, *51*, 6176-6180.

[6] J. Bartsch, V. Prey, *Justus Liebigs Ann. Chem.* **1968**, *717*, 198-204.

[7] S. A. Barker, J. S. Brimacombe, A. B. Foster, D. H. Whiffen, G. Zweifel, *Tetrahedron* **1959**, *7*, 10-18.

[8] S. J. Angyal, R. M. Hoskinson, *J. Chem. Soc. (Resumed)* **1962**, 2991-2995.

[9] A. R. H. Cole, G. T. A. Müller, D. W. Thornton, R. L. S. Willix, *J. Chem. Soc. (Resumed)* **1959**, 1218-1222.

[10] A. J. Michell, H. G. Higgins, *Tetrahedron* **1965**, *21*, 1109-1120.

[11] A. J. Michell, H. G. Higgins, *Cellulose* **1999**, *6*, 89-91.

[12] L. P. Kuhn, *J. Am. Chem. Soc.* **1952**, *74*, 2492-2499.

[13] L. P. Kuhn, *J. Am. Chem. Soc.* **1954**, *76*, 4323-4326.

[14] P. R. Muddasani, E. Bozó, B. Bernet, A. Vasella, *Helv. Chim. Acta* **1994**, *77*, 257-290.

[15] H. Amarasekara, S. Dharuman, T. Kato, D. Crich, *J. Org. Chem.* **2018**, *83*, 881-897.

[16] M. J. Frisch, G. W. Trucks, H. B. Schlegel, G. E. Scuseria, M. A. Robb, J. R. Cheeseman, G. Scalmani, V. Barone, G. A. Petersson, H. Nakatsuji, X. Li, M. Caricato, A. V. Marenich, J. Bloino, B. G. Janesko, R. Gomperts, B. Mennucci, H. P. Hratchian, J. V. Ortiz, A. F. Izmaylov, J. L. Sonnenberg, Williams, F. Ding, F. Lipparini, F. Egidi, J. Goings, B. Peng, A. Petrone, T. Henderson, D. Ranasinghe, V. G. Zakrzewski, J. Gao, N. Rega, G. Zheng, W. Liang, M. Hada, M. Ehara, K. Toyota, R. Fukuda, J. Hasegawa, M. Ishida, T. Nakajima, Y. Honda, O. Kitao, H. Nakai, T. Vreven, K. Throssell, J. A. Montgomery Jr., J. E. Peralta, F. Ogliaro, M. J. Bearpark, J. J. Heyd, E. N. Brothers, K. N. Kudin, V. N. Staroverov, T. A. Keith, R. Kobayashi, J. Normand, K. Raghavachari, A. P. Rendell, J. C. Burant, S. S. Iyengar, J. Tomasi, M. Cossi, J. M. Millam, M. Klene, C. Adamo, R. Cammi, J. W. Ochterski, R. L. Martin, K. Morokuma, O. Farkas, J. B. Foresman, D. J. Fox, Wallingford, CT, **2016**.

[17] M. Bursch, J. M. Mewes, A. Hansen, S. Grimme, *Angew. Chem. Int. Ed. Engl.* **2022**, *61*, e202205735.

[18] E. A. Larsson, J. Ulicny, A. Laaksonen, G. Widmalm, *Org. Lett.* **2002**, *4*, 1831-1834.

[19] A. V. Marenich, C. J. Cramer, D. G. Truhlar, *J. Phys. Chem. B* **2009**, *113*, 6378-6396.

[20] P. W. Kenny, *J. Chem. Inf. Mod.* **2009**, *49*, 1234-1244.

[21] R. E. Rosenberg, R. L. Abel, M. D. Drake, D. J. Fox, A. K. Ignatz, D. M. Kwiat, K. M. Schaal, P. R. Virkler, *J. Org. Chem.* **2001**, *66*, 1694-1700.

[22] M. Matwiejuk, J. Thiem, *Chem. Commun.* **2011**, *47*, 8379-8381.

[23] V. Cachatra, A. Almeida, J. Sardinha, S. D. Lucas, A. Gomes, P. D. Vaz, M. H. Florêncio, R. Nunes, D. Vila-Viçosa, M. J. Calhorda, A. P. Rauter, *Org. Lett.* **2015**, *17*, 5622-5625.

[24] R. Kaifu, L. C. Plantefaber, I. J. Goldstein, *Carbohydr. Res.* **1985**, *140*, 37-49.

[25] P. L. Barili, G. Berti, D. Bertozzi, G. Catelani, F. Colonna, T. Corsetti, F. D'Andrea, *Tetrahedron* **1990**, *46*, 5365-5376.

[26] J. C. Lewis, S. Bastian, C. S. Bennett, Y. Fu, Y. Mitsuda, M. M. Chen, W. A. Greenberg, C.-H. Wong, F. H. Arnold, *Proc. Natl. Acad. Sci. U. S. A.* **2009**, *106*, 16550-16555.

[27] M. Matwiejuk, J. Thiem, *Eur. J. Org. Chem.* **2012**, *2012*, 2180-2187.

[28] M. Moume-Pymbock, T. Furukawa, S. Mondal, D. Crich, *J. Am. Chem. Soc.* **2013**, *135*, 14249-14255.

[29] D. Sinnaeve, M. Foroozandeh, M. Nilsson, G. A. Morris, *Angew. Chem. Int. Ed.* **2016**, *55*, 1090-1093.

[30] D. Sinnaeve, J. Ilgen, M. E. Di Pietro, J. J. Primozic, V. Schmidts, C. M. Thiele, B. Luy, *Angew. Chem. Int. Ed.* **2020**, *59*, 5316-5320.

[31] F. X. Cantrelle, E. Boll, D. Sinnaeve, *Anal. Chem.* **2024**, *96*, 7056-7064.
